# Policy implementation and recommended actions to create healthy food environments using the Healthy Food Environment Policy Index (Food-EPI): a comparative analysis in South Asia

**DOI:** 10.1016/j.lansea.2024.100428

**Published:** 2024-06-26

**Authors:** Elisa Pineda, Petya Atanasova, Nalinda Tharanga Wellappuli, Dian Kusuma, Himali Herath, Alexa Blair Segal, Stefanie Vandevijvere, Ranjit Mohan Anjana, Abu Ahmed Shamim, Saira Afzal, Fahmida Akter, Faiza Aziz, Ananya Gupta, Abu Abdullah Hanif, Mehedi Hasan, Renuka Jayatissa, Sujeet Jha, Vinitaa Jha, Prasad Katulanda, Khadija Irfan Khawaja, Balachandran Kumarendran, Menka Loomba, Sara Mahmood, Malay Kanthi Mridha, Rajendra Pradeepa, Garudam Raveendiran Aarthi, Akansha Tyagi, Anuradhani Kasturiratne, Franco Sassi, Marisa Miraldo

**Affiliations:** a Centre for Health Economics and Policy Innovation, Imperial College Business School, London, United Kingdom; b The George Institute for Global Health UK, School of Public Health, Imperial College London, London, United Kingdom; c Section for Nutrition Research, Department of Metabolism, Digestion and Reproduction, Faculty of Medicine, Imperial College London, London, United Kingdom; d Department of Health Services Research and Management, School of Health & Psychological Sciences, City University of London, London, United Kingdom; e Ministry of Health, Colombo, Sri Lanka; f Scientific Institute of Public Health (Sciensano), Brussels, Belgium; g Madras Diabetes Research Foundation, Chennai, India; h Centre for Non-communicable Diseases and Nutrition, BRAC James P Grant School of Public Health, BRAC University, Dhaka, Bangladesh; i King Edward Medical University, Lahore, Pakistan; j Department of Epidemiology and Biostatistics, School of Public Health, Imperial College London, United Kingdom; k Institute of Endocrinology, Diabetes & Metabolism, Max Super Speciality Hospital, New Delhi, India; l Nutrition Department, Medical Research Institute, Ministry of Health, Colombo, Sri Lanka; m Institute of Diabetes, Endocrinology & Metabolism, Max Super Speciality Hospital (A Unit of Devki Devi Foundation), New Delhi, India; n Research & Academics, Clinical Directorate, Max Healthcare, New Delhi, India; o Faculty of Medicine, University of Colombo, Colombo, Sri Lanka; p Institute of Endocrinology & Metabolism, Services Institute of Medical Sciences (SIMS), Lahore, Pakistan; q Faculty of Medicine, University of Jaffna, Kokkuvil, Sri Lanka; r Office of Research, Max Super Speciality Hospital (A Unit of Devki Devi Foundation), New Delhi, India; s Faculty of Medicine, University of Kelaniya, Ragama, Sri Lanka; t Department of Economics and Public Policy, Imperial College Business School, London, United Kingdom

**Keywords:** South Asia, Food policy, Policy monitoring, Food environment, Policy mapping, Public health policy, Food-EPI, Non-communicable diseases

## Abstract

**Background:**

The increasing prevalence of diet-related non-communicable diseases (NCDs) in South Asia is concerning, with type 2 diabetes projected to rise to 68%, compared to the global increase of 44%. Encouraging healthy diets requires stronger policies for healthier food environments.

**Methods:**

This study reviewed and assessed food environment policies in Bangladesh, India, Pakistan, and Sri Lanka from 2020 to 2022 using the Healthy Food Environment Policy Index (Food-EPI) and compared them with global best practices. Seven policy domains and six infrastructure support domains were considered, employing 47 good practice indicators to prevent NCDs. Stakeholders from government and non-governmental sectors in South Asia (n = 148) were invited to assess policy and infrastructure support implementation using the Delphi method.

**Findings:**

Implementation of food environment policies and infrastructure support in these countries was predominantly weak. Labelling, monitoring, and leadership policies received a moderate rating, with a focus on food safety, hygiene, and quality rather than obesity prevention. Key policy gaps prioritized for attention included front-of-pack labelling, healthy food subsidies, unhealthy food taxation, restrictions on unhealthy food promotion, and improvements in school nutrition standards to combat NCDs.

**Interpretation:**

Urgent action is required to expand food policies beyond hygiene and food security measures. Comprehensive strategies targeting NCD prevention are crucial to combat the escalating burden of NCDs in the region.

**Funding:**

This research was funded by the NIHR (16/136/68 and 132960) with aid from the UK Government for global health research. Petya Atanasova also acknowledges funding from the Economic and Social Research Council (ESRC) (ES/P000703/1). The views expressed are those of the authors and not necessarily of the NIHR, the UK government or the ESRC.

**Research in context**

**Evidence before this study**

Non-communicable diseases (NCDs), such as diabetes, cancers, and cardiovascular diseases, caused 69% of all deaths in the South Asia region in 2021, with half (52%) of these deaths being in the 30–69 years age group. The prevalence of NCDs in the region is expected to have an increase, with diabetes alone being projected to increase by 68% by 2045 compared to a 44% projected increase worldwide.

Although, the aetiology of NCDs is multifaceted, it is well documented that unhealthy diet is a leading modifiable risk factor, including diabetes and cardiovascular diseases. Evidence suggests that food environments, food-related policies and supporting infrastructure are key to achieve healthier diets and to reduce the prevalence of NCDs. These have also been found to be instrumental in addressing the socioeconomic gradient associated with nutrition inequalities. Thus, it is imperative for governments to implement preventative policies and actions to mitigate the burden of unhealthy diets. Regular monitoring of policy implementation and benchmarking compared to best practice, over time, or in relation to other countries in the region could stimulate more actions on food environments in the future. However, evidence on the extent of implementation of policies and infrastructure that enable healthy diet in the South Asia region is scant. The Healthy Food Environment Policy Index (Food-EPI) has been proposed by the International Network for Food and Obesity/Non-communicable Diseases Research, Monitoring and Action Support (INFORMAS) to support governments to benchmark and assess their policies, identify, and prioritise policy and infrastructure support actions for the creation of healthy food environments that enable healthy food choices.

**Added value of this study**

We use Food-EPI to systematically map policies and supporting infrastructure (i.e., administration and management of essential operational elements within a country that support policy implementation) that enable healthy nutrition and the prevention of diet related NCDs in Bangladesh, India, Pakistan, and Sri Lanka. In doing so, we assessed the scope of food policy implementation and identified strategic priority actions to create healthier food environments that reduce diet-related NCDs in these countries. Findings allowed us to gain a comprehensive understanding of the gaps in the food environment area, enabling us to identify policy recommendations for the improvement of food environments in the assessed South Asian countries. We conclude that food policy in South Asia focuses on food safety and hygiene as evident by the moderate implementation of policies related to labelling, monitoring, and leadership. All other policies were weakly implemented or non-existent. The countries recognized the need to address the growing levels of overnutrition related NCDs. Finally, we provide a policy agenda focused on creating healthier food environments in South Asia.

**Implications of all the available evidence**

Whilst undernutrition remains a significant public health problem in lower-middle income countries (LMICs), these countries are also increasingly experiencing a shift to overnutrition. Historically, these countries have focused on policies to address food hygiene and undernutrition that are not suitable to prevent the increased availability of energy-dense processed foods associated with overnutrition. Therefore, in line with the Sustainable Development Goals (SDGs), efforts need to be made to equip South Asian countries with policies promoting healthier food environments and restricting the availability, affordability, promotion, and attractiveness of unhealthy foods and beverages.

## Introduction

Non-communicable diseases (NCDs) are the leading cause of morbidity and mortality globally. Compared to other populations, South Asian individuals have an increased risk of Type 2 diabetes and cardiovascular diseases.^1^ The prevalence of diabetes alone in the South Asia region is projected to increase with 68%, from 90 million in 2021 to 151 million by 2045, compared to a 44% projected increase worldwide.^2^

Although the aetiology of NCDs is multifaceted, unhealthy diet has been found to be a leading modifiable risk factor in the prevention of NCDs, including diabetes and cardiovascular diseases.^3^ Evidence suggests that food environments and food-related policies and supporting infrastructure are key to achieve healthier diets and reduce the prevalence of NCDs.^3^ South Asia region is experiencing rapid urbanization and economic growth which have led to a nutritional transition where traditional locally sourced diets dominated by low fat, high fibre foods have been replaced by the consumption of easily accessible, highly marketed ultra-processed foods that are higher in unhealthy fats, sodium and sugar and are associated with increased risks of NCDs.^4^, ^5^, ^6^, ^7^ This transition is driven by various factors, including the economic growth and increased purchasing power making ultra-processed foods more affordable, urbanization and globalization increasing their availability, technological advances and targeted marketing strategies making them more desirable.^6^^,^^7^ To control obesity and NCDs, it is paramount to reduce the risk factors associated with these diseases. Therefore, improving dietary quality is a key policy priority.^8^

Policies, which enable healthy food environments, have been shown to facilitate healthy food choices and help to prevent NCDs.^9^, ^10^, ^11^ The food environment encompasses the multifaceted and interconnected array of factors influencing individuals' access to, choices about, and consumption of food. It includes physical infrastructure, socio-economic conditions, cultural influences, marketing strategies, individual preferences, and everyday incentives, creating a dynamic context that significantly shapes dietary choices and overall nutritional patterns.^12^, ^13^, ^14^ Unhealthy environments foster unhealthy diets by promoting the availability, accessibility and attractiveness of highly palatable, energy-dense but nutrient-poor foods.^14^

The World Health Organization's (WHO's) ‘Tackling NCDs Best Buys’, highlighted the need of a comprehensive global monitoring framework to track trends and to assess progress made in the implementation of national strategies and plans to control and reduce NCDs.^15^ Furthermore, The Lancet Diabetes Commission indicated the importance of creating a healthy food environment in the prevention and control of NCDs, underpinning the need to track trends and assess progress made in the implementation of policies aimed at creating a healthy food environment to reduce NCDs.^16^ One global monitoring tool that does that is the Healthy Food Environment Policy Index (Food-EPI) which was developed as a standardized tool for governments to assess the progress made in the implementation of policies and to prioritize future actions for the creation of healthy food environments to reduce NCDs.^17^

As of 2022, 42 countries have measured the extent of food environment policy implementation using Food-EPI to identify policy gaps and an agenda for change.^17^ However, this remains as a gap in South Asia. Notably, most of the countries in the South Asia region have multisectoral action plans to prevent and control NCDs and have set time bound targets on NCDs risk factors and management, though progress towards the prevention of unhealthy diets has been limited.^18^ In addition, previous studies document the availability of nutritional policies and programs towards achieving the global nutrition targets in South Asia, they did not specifically assess policies and infrastructure on healthy food environments in the region.^19^ Therefore, the aim of this study was to map food policies and supporting infrastructure in Bangladesh, India, Pakistan, and Sri Lanka, benchmark and assess its level of implementation, and identify policy-level priority actions for the primary prevention of diet-related NCDs using the Food-EPI framework.

## Methods

The present study adopted a cross-sectional observational study design. Our data collection and analyses evolved over four steps. First, we systematically reviewed evidence on policies and infrastructure in Bangladesh, India, Pakistan, and Sri Lanka in 2020-21. We used the Healthy Food-EPI framework by the International Network for Food and Obesity/Non-communicable Diseases Research, Monitoring and Action Support (INFORMAS), developed to assess the extent of food policy implementation and identify and prioritise policy and infrastructure support actions to promote healthy diets.^14^^,^^17^^,^^20^ Second, using the Delphi method, we carried out workshops in 2021-22 in each country to evaluate the extent of food policy implementation and the existence of supporting infrastructure which resulted in scorecards for each country. Third, stakeholders identified priority actions for the prevention of diet related NCDs. Fourth, the identified actions were prioritised according to importance and achievability by the stakeholders. Each of these steps is further explained below.

### Policy mapping overview

The study utilized the Preferred Reporting Items for Systematic reviews and Meta-Analyses (PRISMA) framework to review policies and infrastructure support aimed at preventing obesity and diet-related NCDs up to December 2021. The analysis covered policies both under development and terminated, focusing on current implementations during stakeholder rating workshops. The comprehensive approach, detailed in Supplementary Material S3, sought to provide an overarching view of food environment policies across different countries.

### Stakeholder engagement and evaluation

The study prioritized quality over quantity in assembling a diverse panel of up to 50 stakeholders from academia, government, and NGOs in Bangladesh, India, Pakistan, and Sri Lanka. The selected heterogeneous purposive sampling ensured a comprehensive evaluation of food policies through an online rating workshop utilizing the Delphi method. Stakeholders assessed the policies using a five-point Likert scale, focusing on implementation levels against international best practices.

### Priority actions recommendations

Stakeholders formulated recommended actions for governments, ranking them based on importance and achievability. Considerations included impact, equity, feasibility, acceptability, affordability, and efficiency. The combined scores identified actions with the highest priority, aiming to improve food environments and address policy and infrastructure gaps.

### Analysis

The analysis calculated mean ratings and standard deviations for each country's indicators and domains, assessing the proportion of recommendations per country. An inter-rater reliability agreement score was determined using Gwet's AC2 coefficient, measuring consistency among stakeholders' ratings and highlighting areas of high agreement and potential discrepancies.

### Policy mapping

Evidence of policies and infrastructure support focused on the prevention of obesity and diet-related NCDs identified through a policy review conducted using elements of the Preferred Reporting Items for Systematic reviews and Meta-Analyses (PRISMA) framework and checklist (Supplementary Material 1 and 2). All identified policies and infrastructure support actions up until December 2021 were summarized into an evidence document for each country. In the Evidence document we considered policies that were under development or had already terminated to have a general overview of the policies that had been implemented for each Food-EPI indicator in each country. However, during the rating workshop, we asked stakeholders to only rate the implementation level of policies that were currently implemented. The detailed methods on how the policy review was undertaken, including the inclusion and selection criteria can be found in the Supplementary Material 3.

The Food-EPI allowed us to map, assess, and benchmark the implementation of food environment policies and supporting infrastructure compared with international best practices and prioritize actions to fill implementation gaps, explained in detail below.^21^

**Fig. 1:**
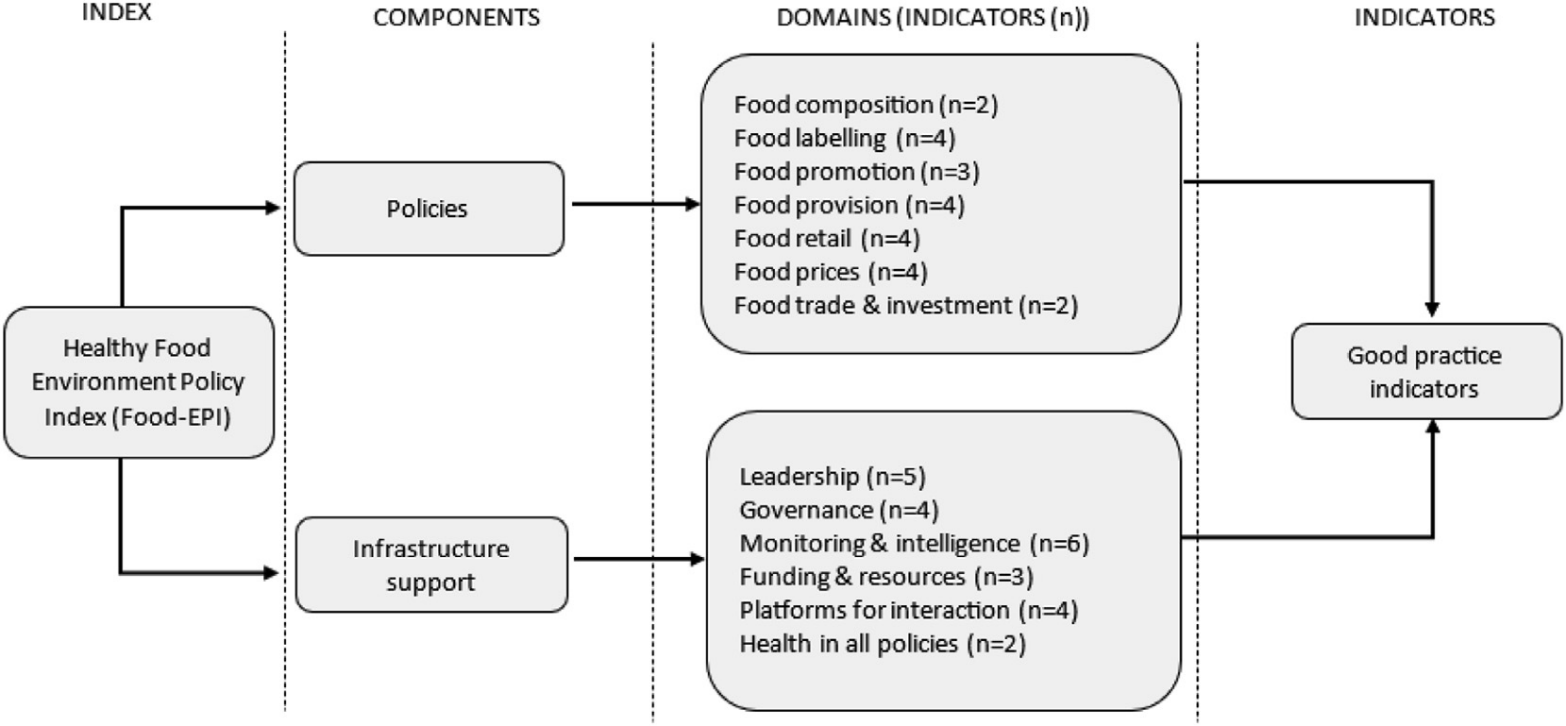
**Healthy food environment policy index (Food-EPI)**.

The Food-EPI tool includes two components: policy and infrastructure support.^20^ The policy component has seven domains: *Food composition, Food labelling, Food promotion, Food provision, Food retail, Food prices, and Food trade and investment*. The infrastructure support component contains six domains: *Leadership, Governance, Monitoring and intelligence, Funding and resources, Platforms for interaction*, and *Health in all policies* (Fig. 1). The tool provides a total of 47 good practice indicators to be assessed (Table 1) and enables a nuanced evaluation of policy implementation, fostering accountability through its dynamic, longitudinal assessment capability. A detailed description of the good practice indicators for each domain can be found in the Supplementary Materials 3 and 4.

**Table 1:** Food-EPI indicator definitions by domain.

Domain	Indicator	Indicator detail
**Policy domains**		
Composition	COMP1	1.Food composition targets/standards have been established for processed foods by the government for the content of the nutrients of concern in certain foods or food groups if they are major contributors to population intakes of these nutrients of concern.
	COMP2	2.Food composition targets/standards have been established for out-of-home meals in restaurants by the government for the content of the nutrient of concern in certain foods or food groups if they are major contributions to population
Labelling	LABEL1	1.Ingredient lists and nutrient declarations in line with Codex recommendations are present on the labels of all packaged foods.
	LABEL2	2.Robust, evidence-based regulatory systems are in place for approving/reviewing claims on foods so that consumers are protected against unsubstantiated and misleading nutrition and health claims.
	LABEL3	3.A single, consistent, interpretive, evidence-informed front-of-pack supplementary nutrition information system, which readily allows consumers to assess a product's healthiness, is applied to all packaged foods.
	LABEL4	4.A consistent, single, simple, clearly visible system of labelling the menu boards of all quick-service restaurants is applied by the government, which allows consumers to interpret the nutrient quality and energy content of foods on sale.
Promotion	PROMO1	1.Effective policies are implemented by the government to restrict exposure and power of promotion of unhealthy foods including to children through broadcast media (e.g., TV, radio).
	PROMO2	2.Effective policies are implemented by the government to restrict exposure and power of promotion of unhealthy foods including to children through non-broadcast media (e.g., Internet, social media, food packaging, sponsorship, outdoor advertising)
	PROMO3	3.Effective policies are implemented by the government to ensure that unhealthy foods are not commercially promoted including to children in settings where children gather (e.g., preschools, schools, sport, and cultural events).
Prices	PRICES1	1.Taxes on healthy foods are minimized to encourage healthy food choices where possible (e.g., low or no sales tax, excise, value-added or import duties on fruit and vegetables).
	PRICES2	2.Taxes on unhealthy foods (e.g., sugar-sweetened beverages, foods high in nutrients of concern) are in place to discourage unhealthy food choices where possible, and these taxes are reinvested to improve population health.
	PRICES3	3.The intent of existing subsidies on foods, including infrastructure funding support (e.g., research and development, supporting markets or transport systems), is to favour healthy rather than unhealthy foods.
	PRICES4	4.The government ensures that food-related income support programs are for healthy foods.
Provision	PROV1	1.The government ensures that there are clear, consistent policies (including nutrition standards) implemented in schools and early childhood education services for food service activities (canteens, food at events, fundraising, promotions, vending machines) to provide/promote healthy food choices.
	PROV2	2.The government ensures that there are clear, consistent policies in other public sector settings for food service activities to provide/promote healthy food choices.
	PROV3	3.The government ensures that there are good support and training systems to help schools and other public sector organizations and their caterers meet the healthy food service policies and guidelines.
	PROV4	4.Government actively encourages and supports private companies to provide and promote healthy foods and meals in their workplaces.
Retail	RETAIL1	1.Zoning laws and policies are robust enough and are being used, where needed, by local governments to place limits on the density or placement of quick-serve restaurants or other outlets selling mainly unhealthy foods in communities.
	RETAIL2	2.Zoning laws and policies are robust enough and are being used, where needed, by local governments to encourage the availability of outlets selling fresh fruit and vegetables.
	RETAIL3	3.The Government ensures existing support systems are in place to encourage food stores to promote the in-store availability of healthy foods and to limit the in-store availability of unhealthy foods.
	RETAIL4	4.The government ensures existing support systems are in place to encourage food service outlets (e.g., restaurants) to increase the promotion and availability of healthy foods and to decrease the promotion and availability of unhealthy foods.
Trade	TRADE1	1.The Government undertakes risk impact assessments before and during the negotiation of trade and investment agreements, to identify, evaluate and minimize the direct and indirect negative impacts of such agreements on population nutrition and health.
	TRADE2	2.The government adopts measures to manage investment and protect their regulatory capacity with respect to public health nutrition.
**Infrastructure and support domains**		
Leadership	LEAD1	1.There is strong, visible, political support (at the Head of Government/Cabinet-level) for improving food environments, population nutrition, diet-related NCDs and their related inequalities.
	LEAD2	2.Clear population intake targets have been established by the government for the nutrients of concern (e.g., fat, salt, sugar) to meet WHO and national recommended dietary intake levels.
	LEAD3	3.Clear, interpretive, evidence-informed food-based dietary guidelines have been established and implemented.
	LEAD4	4.There is a comprehensive, transparent, up-to-date implementation plan linked to national needs and priorities, to improve food environments, reduce the intake of the nutrients of concern to meet WHO and national recommended dietary intake levels, and reduce diet-related NCDs.
	LEAD5	5.Government priorities have been established to reduce inequalities or protect vulnerable populations in relation to diet, nutrition, obesity, and NCDs.
Governance	GOV1	1.There are robust procedures to restrict commercial influences on the development of policies related to food environments where they have conflicts of interest with improving population nutrition.
	GOV2	2.Policies and procedures are implemented for using evidence in the development of food policies.
	GOV3	3.Policies and procedures are implemented for ensuring transparency in the development of food policies
	GOV4	4.The government ensures access to comprehensive nutrition information and key documents (e.g., budget documents, annual performance reviews, and health indicators) for the public.
Monitoring	MONITOR1	1.Monitoring systems, implemented by the government, are in place to regularly monitor food environments (especially for food composition for nutrients of concern, food promotion to children, and nutritional quality of food in schools and other public sector settings), against codes/guidelines/standards/targets.
	MONITOR2	2.There is regular monitoring of adult and childhood nutrition status and population intakes against specified intake targets or recommended daily intake levels.
	MONITOR3	3.There is regular monitoring of adult and childhood overweight and obesity prevalence using anthropometric measurements
	MONITOR4	4.There is regular monitoring of the prevalence of NCD risk factors and occurrence rates (e.g., prevalence, incidence, mortality) for the main diet-related NCDs.
	MONITOR5	5.There is sufficient evaluation of major programs and policies to assess effectiveness and contribution to achieving the goals of the nutrition and health plans.
	MONITOR6	6.Progress towards reducing health inequalities or health impacts in vulnerable populations and societal and economic determinants of health are regularly monitored.
Funding	FUND1	1.The ‘Population Nutrition Promotion’ budget, as a proportion of total health spending and/or in relation to the diet-related NCD burden is sufficient to reduce diet-related NCDs.
	FUND2	2.Government-funded research is targeted for improving food environments, reducing obesity, NCDs, and their related inequalities.
	FUND3	3.There is a statutory health promotion agency in place that includes an objective to improve population nutrition, with a secure funding stream.
Platforms	PLATFORM1	1.There are robust coordination mechanisms across departments and levels of government (national and local) to ensure policy coherence, alignment, and integration of food, obesity and diet-related NCD prevention policies across governments
	PLATFORM2	2.There are formal platforms between the government and the commercial food sector to implement healthy food policies.
	PLATFORM3	3.There are formal platforms for regular interactions between government and civil society on food policies and other strategies to improve population nutrition.
	PLATFORM4	4.The government leads a broad, coherent, effective, integrated, and sustainable systems-based approach with local organizations to improve the healthiness of food environments at a national level.
Health in all policies	HIAP1	1.There are processes in place to ensure that population nutrition, health outcomes, and reducing health inequalities or health impacts in vulnerable populations are considered and prioritized in the development of all government policies relating to food.
	HIAP2	2.There are processes (e.g., health impact assessments) to assess and consider health impacts during the development of other non-food policies.

Source: INFORMAS, 2017.^21^

The health promoting food environment policies and infrastructure support were assessed against international best practice examples (i.e., comprehensive examples of policy implementation worldwide chosen based on their strength and comprehensiveness).^22^ The compiled policy evidence was validated by government officials from the public health ministry from each country to ensure all relevant policy documents had been included. All policy evidence documents from participating countries received suggestions of policy additions after the revision. These suggestions focused on initiatives that were in place but were not currently implemented policies.

### Evaluation workshops—extent of food policy implementation

We employed a heterogeneous purposive sampling method to invite up to 50 stakeholders from academia, government, and non-governmental organizations (NGOs) in Bangladesh, India, Pakistan, and Sri Lanka, following similar research.^23^^,^^24^ This approach sought to balance expert viewpoints diversity with manageable panel size, aiming to minimize information bias and produce contextually relevant findings.

We collected information about the stakeholder's name, type of actor, expertise, their organization, role, and level. Stakeholders who met the inclusion criteria and had no conflicts of interest were invited to attend an online rating workshop in which the policy evidence document was explained and provided.

Stakeholder inclusion criteria encompassed having expertise in public health, nutrition, and/or food policy, living in the country where the policy assessment was being undertaken, or being a relevant actor in the academic, government/government agencies, and/or non-profit sector. Exclusion criteria encompassed food industry stakeholders and stakeholders with no expertise in nutrition, NCD prevention and/or food policy.^20^

We conducted the online rating workshop using the Delphi method (i.e., an interactive process which aims to develop an expert-based judgment about an epistemic question through a structured group communication processes to evaluate an unknown issue by stakeholders)^25^^,^^26^ to elicit stakeholders’ consensus on the evaluation of the food policies. Workshops were undertaken online due to the COVID-19 pandemic restrictions on travel and social distancing in 2020–2022.

In the rating workshop, stakeholders were first instructed to individually assess the implementation level of each policy and infrastructure support indicator (in comparison to the international best-practice policy examples from the compiled policy evidence document) using a five-point Likert scale (Supplementary Material 5). To facilitate evaluation, a presentation on the relevant indicator, its definition, best practice examples and the relevant identified country policies were provided. During the rating workshop, we asked stakeholders to only rate the implementation level of policies that were currently implemented. Precise details of this are available within the policy evidence document for each country (Supplementary Materials 6–9). The Likert scale used by stakeholders to evaluate the level of policy implementation for each of the 47 Food-EPI indicators based on a Likert scale of 1–5 in which ‘1’ represented the policy did not exist (non-existent), ‘2’ indicated the implementation of the policy was weak, ‘3’ the implementation of the policy was moderate, ‘4’ the implementation of the policy was strong, and ‘5’ the implementation of the policy was very strong. There was also an option for ‘I do not know/cannot rate’ included. Disaggregated by country, the policy rating for each of the 47 Food-EPI indicators was averaged and presented to the stakeholder panel for discussion. This group discussion was guided by the workshop moderators to identify policy gaps and potential government recommended actions within each country.

### Priority actions recommendations

Stakeholders in each participating country were asked to formulate recommended actions for the government based on the policy and infrastructure support domains to improve food environments.

Stakeholders received instructions for how to rank the recommended actions based on importance and achievability. This was undertaken individually by each of the stakeholders. To rank actions according to their importance, stakeholders were asked to consider the need (i.e., size of the implementation gap), impact (i.e., the effectiveness of the action on improving food environments and diets), equity (i.e., progressive/regressive effects on reducing food/diet related health inequalities), other positive and negative effects of the recommended action. For achievability stakeholders were asked to consider; relative feasibility (i.e., how easy, or hard the action is to be placed); acceptability (i.e., the level of support from key stakeholders including government, the public, public health, and industry); affordability (i.e., the cost of implementing the action); and efficiency (i.e., the cost-effectiveness of the recommended action). To identify the recommended actions with the highest priority, the sum of the scores (rankings of all stakeholders) was calculated for each action. The total score for each action was calculated considering the scores on both importance and achievability.

### Analyses

Using the gathered data from the rating workshops, we calculated the indicator and domain rating mean and standard deviation for each country. We then used data from the priority actions recommendations workshop to calculate the proportion of recommendations per country for each for the Food-EPI indicators and domains. In addition, to identify whether the recommendations were targeting policy or infrastructure implementation gaps, for each Food-EPI indicator, we identified the extent of implementation for each recommended action. Lastly, we identified recommended actions with the highest level of importance and achievability for the policy and infrastructure support domains.

An inter-rater response rate (IRR) was applied to evaluate the differences in response rates among stakeholders when rating the various policy and infrastructure support domains and indicators. This assessment was conducted through a weighted analysis using Gwet's AC2 coefficient (AgreeStat 2015.6.2). A high IRR score (>0.5) indicates high reliability or agreement on among raters.

### Research ethics approval

This study was granted ethics approval by the ethics committees in all participating countries (Supplementary Material 2). Local stakeholder involvement and collaboration was carried out in South Asia for the design, data collection and dissemination plan of the research. Data were systematically collected by academics from, Bangladesh, India, Pakistan, and Sri Lanka.

### Role of the funding source

This research was commissioned by National Institute for Health Research (NIHR) Global Health Research Units and Groups using UK aid from the UK Government. The funder had no role on the study design, collection, analysis, or interpretation of the data, in the writing of the report; nor in the decision to submit the paper for publication.

## Results

### Characteristics of stakeholders and response rates across countries

**Fig. 2:**
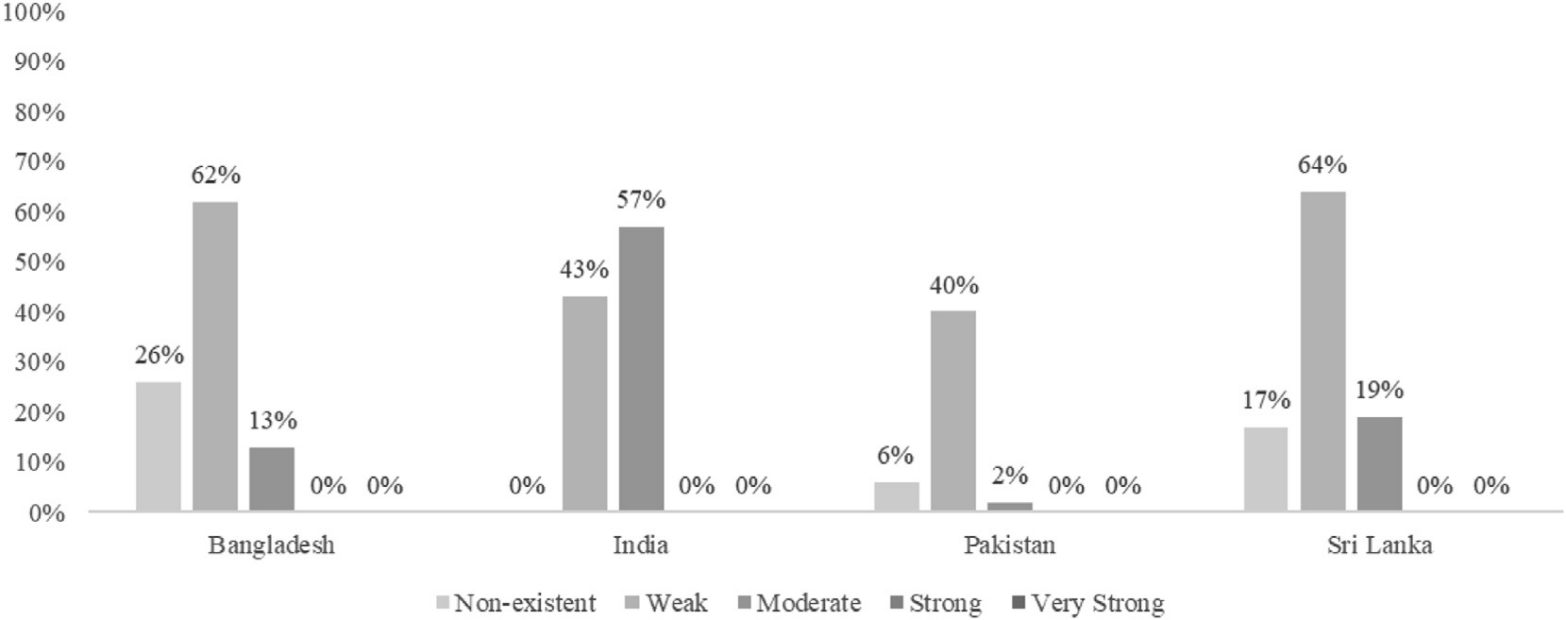
**Stakeholder rating results by country compared against international best practice**. The numbers represent the percentage of indicators ranked according to level of implementation by country. For example, 62% of all indicators in Bangladesh were weakly implemented.

The invitation was sent to 148 stakeholders across the four countries. Of those stakeholders, 104 (72%) took part, with Pakistan having the highest response rate at 94% and the lowest at 50% in India (Table 2, Fig. 2). According to their organisation, participants were classified into the following categories: academia (n = 43), NGOs and other organisations (n = 15), and government (n = 46).

**Table 2:** Response rates by country.

Country	Year	Stakeholders invited (N)	Response rate N (%)	Academia N (%)	NGOs & other organisations N (%)	Government and intergovernmental stakeholders N (%)
Bangladesh	2022	34	19 (56%)	9 (47%)	3 (16%)	7 (37%)
India	2022	30	15 (50%)	5 (33%)	7 (47%)	3 (20%)
Pakistan	2022	34	29 (85%)	15 (52%)	0 (0%)	14 (48%)
Sri Lanka	2021	50	38 (76%)	14 (37%)	5 (13%)	19 (50%)

Note: N, number of respondents.

Most of the academic participants consisted of researchers at universities, with occasional participants from think tanks. Participants from the NGOs included mainly individuals from major health and nutrition organisations, private nutrition consultancies, research foundations, and an occasional expert from an independent charity. The government representatives included individuals from government ministries (such as for the health or nutrition sector) or connected government institutes.

The inter-rater reliability was assessed separately by country using Gwet's AC2. The overall inter-rater reliability was 0.75 (95% CI 0.75, 0.75) for Bangladesh, 0.62 (95% CI 0.62, 0.62) for India, 0.72 (0.72, 0.72) for Pakistan, 0.66 (95% CI 0.66, 0.66) for Sri Lanka, all of which are considered moderate to excellent agreement. The confidence interval (CI) for these scores, being narrow and consistent, indicated precision in the reliability estimates.

Tables 3 and 4 present results of the benchmarking ratings across the Food-EPI domains for each of the countries (i.e., food composition, food labelling, regulation of food marketing, food prices, food provision in public institutions and on worksites, food retail, international trade and investment, political leadership and official dietary guidelines, governance, monitoring and surveillance, funding, platforms for interaction between government, academia, civil society and the food industry and inter-sectoral approaches). Across the four countries, none of the indicators garnered a ‘strong’ or ‘very strong’ rating. Whilst this doesn't explicitly imply the absence of policies or supporting infrastructure, it does highlight an opportunity for enhancement in the current strategies aimed at preventing non-communicable diseases. The moderate or weak ratings suggest a potential misalignment of these policies with international best practices.

**Table 3:** Overview of benchmarked indicators by domain and country.

A) Average implementation of benchmarked indicators by domain and country.				
	Bangladesh	India	Pakistan	Sri Lanka
Policies domain				
Food composition	Weak	Weak	Weak	Weak
Food labelling	Weak	Medium	Weak	Weak
Food promotion/marketing	Weak	Weak	Weak	Weak
Food prices	Weak	Medium	Weak	Weak
Food provision	Weak	Weak	Weak	Weak
Food retail	Non-existent	Weak	Non-existent	Non-existent
Trade	Weak	Weak	Weak	Weak
Infrastructure support domain				
Leadership	Medium	Medium	Weak	Weak
Governance	Weak	Medium	Weak	Weak
Monitoring	Weak	Medium	Weak	Weak
Funding	Weak	Medium	Non-existent	Weak
Platforms	Weak	Weak	Weak	Weak
Health in all policies	Weak	Weak	Weak	Weak

B) Mean and standard deviation of benchmarked indicators by domain and country.					
Domain	Indicator	Bangladesh	India	Pakistan	Sri Lanka
**A) Policy domains**					
Composition	COMP1	1.79 (0.89)	2.93 (1.27)	1.86 (1.21)	2.19 (0.98)
	COMP2	1.43 (0.76)	2.00 (0.68)	1.75 (1.07)	1.22 (0.75)
	*COMP (mean)*	1.84	1.61	2.46	1.71
Labelling	LABEL1	1.92 (1.12)	3.47 (1.25)	3.15 (0.97)	3.18 (1.21)
	LABEL2	1.54 (1.13)	2.87 (1.36)	2.12 (1.01)	2.52 (1.35)
	LABEL3	1.62 (1.12)	2.13 (1.36)	2.15 (0.92)	2.71 (1.34)
	LABEL4	1.38 (1.19)	2.00 (1.07)	1.92 (1.04)	1.29 (0.99)
	*LABEL (mean)*	1.62	2.62	2.34	2.42
Promotion	PROMO1	1.88 (1.11)	2.27 (0.70)	1.92 (0.83)	1.8 (1.02)
	PROMO2	1.65 (1.06)	2.13 (1.06)	1.71 (1.04)	1.54 (0.95)
	PROMO3	1.76 (1.15)	2.27 (0.88)	1.96 (1)	1.89 (1.08)
	*PROM (mean)*	1.76	2.22	1.86	1.74
Prices	PRICES1	1.47 (0.8)	2.80 (1.01)	1.63 (0.71)	1.44 (0.99)
	PRICES2	1.35 (0.79)	2.67 (1.23)	1.75 (0.9)	1.97 (0.94)
	PRICES3	1.88 (0.6)	2.33 (1.05)	1.71 (0.86)	1.74 (1.09)
	PRICES4	2.24 (0.9)	2.00 (1.31)	1.79 (1.02)	2.09 (1.31)
	*PRICES (mean)*	1.74	2.45	1.72	1.81
Provision	PROV1	1.76 (0.66)	2.93 (0.88)	2.04 (1.06)	2.61 (0.9)
	PROV2	1.59 (0.71)	2.60 (0.83)	1.96 (1.02)	1.97 (1.01)
	PROV3	1.59 (0.71)	2.27 (0.88)	2.08 (1.04)	1.75 (1.11)
	PROV4	1.41 (0.51)	2.27 (0.88)	1.52 (0.77)	1.49 (0.82)
	*PROV (mean)*	1.59	2.52	1.90	1.95
Retail	RETAIL1	1.07 (0.26)	2.00 (0.88)	1.38 (0.88)	1.26 (0.74)
	RETAIL2	1 (0)	2.14 (1.17)	1.5 (0.98)	1.29 (0.8)
	RETAIL3	1.2 (0.41)	1.5 (0.76)	1.46 (1.02)	1.36 (0.9)
	RETAIL4	1.27 (0.46)	2.36 (1.22)	1.52 (0.85)	1.28 (0.74)
	*RETAIL (mean)*	1.13	2.00	1.46	1.30
Trade	TRADE1	1.43 (1.16)	1.80 (1.01)	1.42 (0.90)	1.82 (0.75)
	TRADE2	1.64 (0.84)	2.13 (1.13)	1.77 (0.91)	2.25 (1.04)
	*TRADE (mean)*	1.54	1.97	1.60	2.03
**B) Infrastructure and support domains**					
Leadership	LEAD1	2.93 (1.03)	3.13 (1.3)	2.05 (1.12)	1.83 (1.07)
	LEAD2	2.67 (0.9)	3.13 (1.13)	2.1 (1)	2.08 (0.94)
	LEAD3	2.47 (0.83)	2.93 (1.39)	2.19 (1.17)	2.74 (1.09)
	LEAD4	2.27 (0.96)	2.47 (1.46)	1.43 (0.98)	1.89 (1.01)
	LEAD5	2.67 (1.11)	2.93 (1.22)	1.95 (1.07)	2.09 (0.93)
	*LEAD (mean)*	2.60	2.92	1.94	2.13
Governance	GOVERN1	1.47 (0.83)	2.67 (1.11)	1.48 (0.85)	1.65 (0.8)
	GOVERN2	2.53 (0.83)	3.07 (0.88)	1.96 (0.86)	2.18 (1.04)
	GOVERN3	2.2 (0.68)	2.93 (0.88)	2.04 (1.12)	1.97 (1.02)
	GOVERN4	2.47 (0.92)	3.2 (1.42)	1.79 (1.22)	2.56 (1.05)
	*GOVERN (mean)*	2.17	2.97	1.82	2.09
Monitoring	MONITOR1	1.75 (0.93)	3.13 (0.74)	1.96 (0.98)	2.03 (0.98)
	MONITOR2	2.69 (0.87)	2.80 (1.01)	1.92 (0.91)	2.44 (0.86)
	MONITOR3	2.69 (1.14)	3.20 (1.21)	1.48 (0.65)	2.85 (0.93)
	MONITOR4	2.31 (0.87)	3.47 (1.06)	1.64 (1.04)	3.03 (1)
	MONITOR5	2.38 (1.02)	2.87 (1.30)	1.72 (0.98)	2.15 (1.16)
	MONITOR6	2.25 (0.68)	2.73 (0.96)	1.44 (0.82)	1.91 (0.91)
	*MONITOR (mean)*	2.34	3.03	1.69	2.40
Funding	FUND1	1.31 (0.48)	2.57 (1.02)	1.12 (0.88)	1.61 (0.76)
	FUND2	1.69 (0.63)	2.79 (0.97)	1.46 (0.88)	1.71 (0.68)
	FUND3	2.31 (1.25)	2.71 (1.38)	1.54 (1.18)	2.65 (1.18)
	*FUND (mean)*	1.77	2.69	1.37	1.99
Platforms	PLATFORM1	1.92 (0.76)	2.86 (0.95)	1.52 (1.12)	2.12 (0.93)
	PLATFORM2	1.85 (0.69)	2.07 (1.14)	1.70 (1.11)	1.68 (0.81)
	PLATFORM3	2.23 (0.73)	2.21 (1.12)	1.39 (1.03)	1.88 (0.94)
	PLATFORM4	1.77 (0.73)	2.79 (0.8)	1.39 (0.84)	1.64 (0.82)
	*PLATFORM (mean)*	1.94	2.48	1.50	1.83
Health in all policies	HIAP1	2.17 (1.19)	3.00 (0.76)	1.67 (0.87)	2.16 (0.92)
	HIAP2	1.75 (0.75)	1.67 (1.50)	1.50 (0.88)	1.6 (0.77)
	*HIAP (mean)*	1.96	2.33	1.58	1.88

**Table 4:** Most highly recommended policy actions in terms of importance and achievability by stakeholders in Bangladesh, India, Pakistan, and Sri Lanka.

Indicator	Recommended actions	Importance	Achievability
**Bangladesh**			
Labelling (LABEL3)	To introduce front-of-pack labelling regulations to monitor and strengthen consumer's food choices.	Very high	Very high
Prices (PRICES2)	To impose tax/restrictions on production/import/manufacturing of unhealthy foods and beverages.	Very high	Very high
Promotion (PROMO2)	To ban lucrative food packaging, especially packaging and marketing which target children and adolescents.	Very high	Very high
Promotion (PROMO3)	To implement health promotion in schools and adolescent clubs, creating more demand for healthy food.	Very high	Very high
**India**			
Labelling (LABEL3)	Front-of-pack labelling needs to be implemented.	Very high	Very high
Provision (PROV3)	To implement school-based awareness programmes in which nutrition is taught as a skill set ∗Ability to differentiate between healthy and unhealthy foods ∗Ability to read and interpret labels for food choice ∗Importance of diet diversity	Very high	Very high
Retail (RETAIL1)	The sale of unhealthy foods around schools needs to be regulated.	Very high	High
**Pakistan**			
Composition (COMP1)	Food manufacturing companies should be restricted to manufacture products below prescribed standards.	Very high	High
Labelling (LABEL1)	Adoption of the Codex Alimentarius for setting up food safety and security standards, with a stringent mechanism to track progress.	Very high	Very high
Labelling (LABEL4)	Display of nutritional content at all food areas (restaurants, hotels, canteens, etc.)	Very high	Very high
Prices (PRICES1)	Affordable and accessible healthy food choices for everyone in Pakistan at every level. Cost of healthy foods, including natural foods such as nuts, fruits, vegetables, and herbs, should be low as possible for every individual of society.	Very high	High
Promotion (PROMO3)	Reduce commercial pressures (e.g., marketing of unhealthy foods and beverages) particularly strategies which target children and other populations to consume food products high in fat, salt, and sugar.	High	Very high
Promotion (PROMO2)	Online and cable campaigns should be conducted to regulate the promotion of the unhealthy foods.	Very high	Very high
Provision (PROV1/PROV2/PROV4)	To provide midday healthy meals free of cost in government schools and enable healthy food provision in private settings (e.g., private schools and workplaces)	Very high	High
Provision (PROV3)	To provide nutrition education training at educational settings (e.g., school, college, university) and at community level.	Very high	High
Retail (RETAIL3)	Unhealthy foods rich in sugars, fat, and salt should be slowly removed from the market.	Very high	Very high
**Sri Lanka**			
Composition (COMP1)	To identify food groups and regulations to establish food composition targets for sugar, salt, and saturated fat.	Very high	High
Labelling (LABEL1)	To strengthen the implementation of existing regulation regarding ingredient lists and nutrient declarations for them to be in line with the Codex recommendations on the labels of all packaged foods.	Very high	High
Labelling (LABEL2)	To strengthen the implementation of existing regulation regarding the approval and revision of claims on foods so that consumers are protected against unsubstantiated and misleading nutrition and health claims.	Very high	High
Prices (PRICES1)	To reduce the tax on fruits and vegetables.	Very high	High
Prices (PRICES1)	To consider inflation and effective tax rates on foods high in fat, sugar and salt and sugar-sweetened beverages.	Very high	High
Prices (PRICES4)	To streamline existing programmes.	Very high	High
Promotion (PROMO1)	To strengthen the implementation of existing unhealthy food marketing regulations and speed up the legal mechanisms.	Very high	High
Retail (RETAIL3)	To implement support systems to encourage supermarkets to promote the in-store availability of healthy foods.	Very high	High

### Bangladesh

For 62% of the indicators, the degree of implementation in Bangladesh relative to international best practices was rated as weak, 13% as moderate, and 26% were non-existent (Fig. 2). All indicators in the policy domains were rated with weak implementation, with the highest average ratings observed for policies related to *prices* as the government provides food related income support for the poorest and *labelling* as there are policies for all packaged foods to be labelled according to Codex recommendations (Table 3 and Supplementary Material 6). *Food retail* policies were rated as non-existent; stakeholders could not identify zoning laws and policies in place to increase (decrease) the availability of healthy (unhealthy) foods nor to promote (restrict) the availability of healthy (unhealthy) food or beverage products at the retail level (Table 3 and Supplementary Material 6).

All policies in the infrastructure support domain were rated with weak implementation, except for *leadership* and *monitoring* which were rated with medium implementation (Table 3). In terms of *leadership*, the data suggests that the government has developed strategies and put initiatives in place to ensure food safety and nutrient goals to improve the nutrition of the population as evident by the formulation of the Food Safety Regulations 2017 (Supplementary Material 6). For *monitoring*, the evidence indicates that there is regular monitoring of adult and child nutritional status and obesity prevalence (Supplementary Material 6).

### India

Only India was reported to have food policies and infrastructure support in place for all Food-EPI domains as 43% of the indicators were rated as weak and 57% as moderate (Fig. 2). In the policy domain, the indicators rated with moderate implementation are related to *labelling* as all packaged foods are labelled in line with Codex recommendations to have all ingredients listed on the package; *composition* as the government introduced regulations on the content of salt, sugar and fat in food products; *prices* as fruit and vegetables are tax-free and there is a “Fat Tax” on unhealthy foods; and *provision* as the promotion of foods high in fat, sugar and salt is prohibited in schools (Supplementary Material 7).

In the infrastructure support domain, almost all indicators (83%) were rated moderately, with the highest average ratings observed for *monitoring* as there is regular monitoring of the prevalence of NCDs risk factors and occurrence rates (Table 3 and Supplementary Material 8). Only some policies related to *health in all policies* and *platforms* were rated as weak as there are no processes to assess the health impacts during the development of other non-food related policies, and there are no formal platforms between the government and commercial sector and civil society on food policies for improving population health (Table 3 and Supplementary Material 7).

### Pakistan

In Pakistan, only 2% of the indicators were rated as moderate, whilst 75% of the indicators were rated as weak and 23% were non-existent (Fig. 2). All policies related to *food retail* and *funding* were rated as non-existent because there are no zoning laws and policies to restrict/promote selling unhealthy/healthy foods and there is no support system in place to encourage food outlets to decrease/increase availability of unhealthy/healthy foods in-stores, and insufficient funding was being invested in the population to prevent diet related NCDs (e.g., no government funded research targeted for improving food environments and no statutory health promotion agency in place) (Table 3 and Supplementary Material 8). Only one policy related to *labelling* was rated with moderate implementation as all packaged foods are labelled according to the Codex recommendations to have all ingredients listed on the package (Table 3 and Supplementary Material 8).

### Sri Lanka

19% of the indicators were rated as moderate, 64% of the indicators were rated as weak and 17% as non-existent. Like other countries, *food retail* policies were non-existent there are no zoning laws and policies to restrict/promote selling unhealthy/healthy foods and there is no support system in place to encourage food outlets to decrease/increase availability of unhealthy/healthy foods in-stores (Table 3 and Supplementary Material 9).

In the policy domain indicators related to *labelling* and *provision* were rated with moderate implementation whilst all other policies were rated as weak (Table 3). In terms of *labelling*, there is evidence that packaged foods are labelled in line with Codex recommendations to have all ingredients listed on the package, and there is a colour coded front-of-pack labelling requirement based on the sugar, salt, and fat contents, as well as there is an established regulatory framework to review claims on food label (Supplementary Material 10). For provision, the government introduced school canteen policies indicating healthy and unhealthy foods (Supplementary Material 9).

In the infrastructure support domain, the indicators rated with moderate implementation were *monitoring*, *leadership* and *funding* as the government regularly monitors the prevalence of NCD risk factors and occurrence rates among adults and children; the government has established dietary guidelines providing information on healthy meals and recommended portion sizes; and there is a statutory health promotion agency in place (Table 3 and Supplementary Material 9).

### Proposed and prioritized actions

**Fig. 3:**
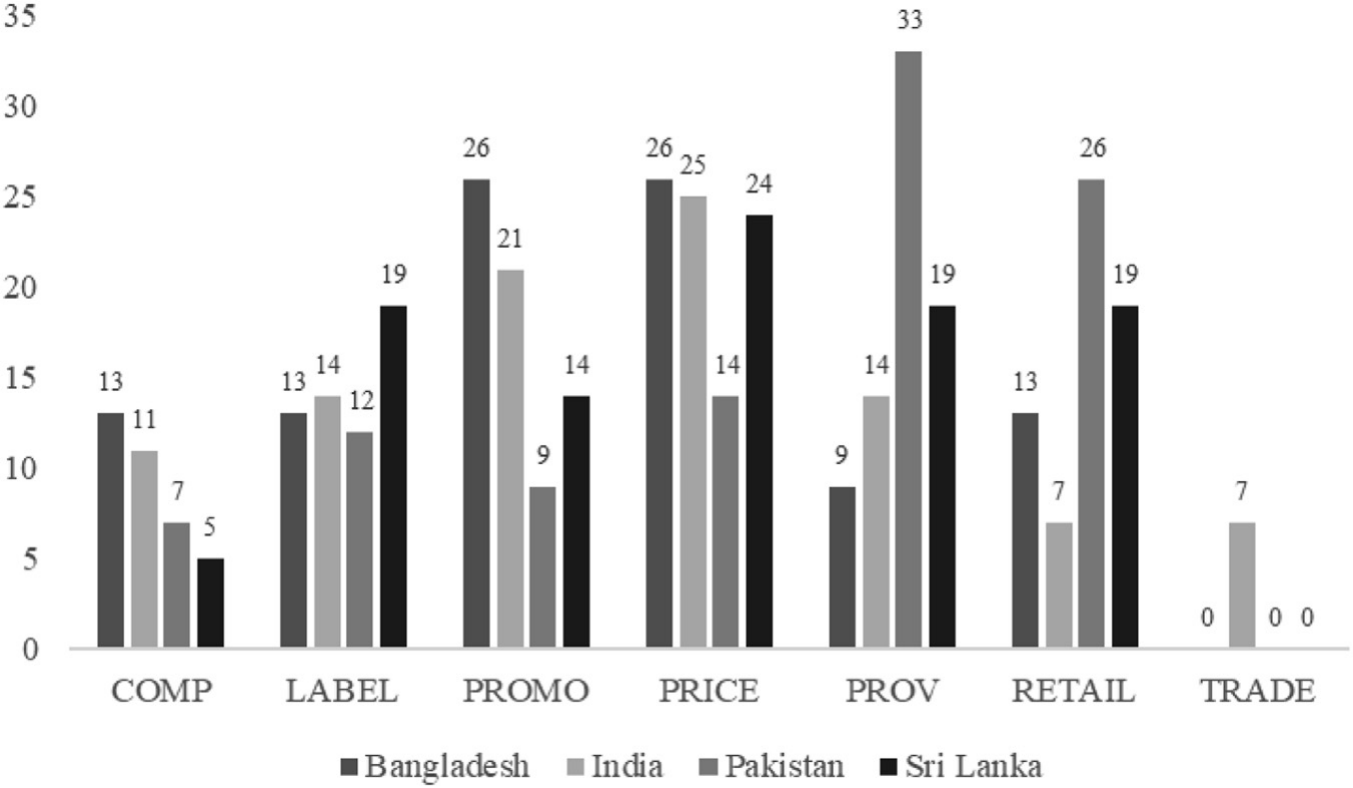
**Recommended policy actions as a proportion of the total number of actions prioritised in each country.** COMP stands for the Food Composition domain; LABEL stands for the Food Labelling domain; PROMO stands for the Food Promotion domain; PRICE stands for the Food Prices domain; PROV stands for the Food Provision domain; RETAIL stands for the Food Retail domain; and TRADE stands for the Food Trade & Investment domain. Details about each domain can be found in Table 1.

**Fig. 4:**
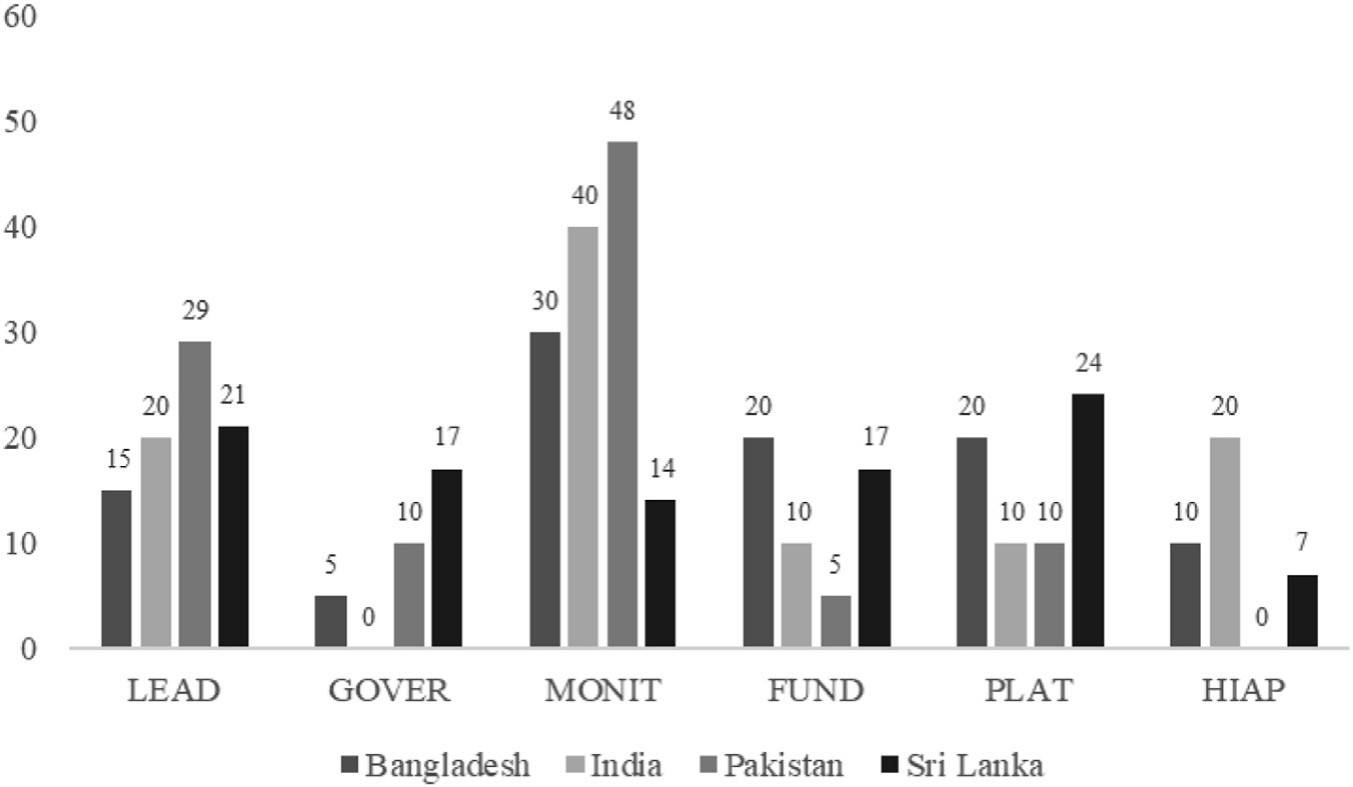
**Recommended infrastructure support actions as a proportion of the total number of actions prioritised in each country**. LEAD stands for the Leadership domain; GOVER stands for the Governance domain; MONIT stands for the Monitoring & Intelligence domain; FUND stands for the Funding & Resources domain; PLAT stands for the Platforms for Interaction domain; and HIAP stands for the Health in all policies domain. Details for each domain can be found in Table 1.

A complete list of prioritized policy action recommendations can be found in Table 4 and prioritized infrastructure support action recommendations in Table 5. Graphs depicting the assigned scores for recommendation is in Fig. 3 for policy actions and Fig. 4 for infrastructure actions. In what follows we discuss the top recommended policies in terms of level of importance and achievability–complete information on these recommendations, can be found in Supplementary Material 10.

**Table 5:** Most highly recommended infrastructure support actions in terms of importance and achievability by stakeholders in Bangladesh, India, Pakistan, and Sri Lanka.

Indicator	Recommended actions	Importance	Achievability
**Bangladesh**			
Monitoring (MONIT1)	Establish effective monitoring and regulation systems.	Very high	Very high
Monitoring (MONIT5)	More investment in monitoring NCDs prevalence and of food policy implementation to benchmark and identify effective approaches to improve food environments in Bangladesh.	Very high	Very high
Platforms (PLATF1)	Need to strengthen the interorganizational collaboration implementing the existing policies.	Very high	Very high
Platforms (PLATF2)	Enforcement of existing policies, monitoring of implementation, and improved coordination among various stakeholders including the private sector.	Very high	Very high
**India**			
Leadership (LEAD2)	Awareness creation needs to be intensified among all stakeholders to differentiate between healthy and unhealthy foods.	Very high	High
**Pakistan**			
Governance (GOVERN1)	Governance and accountability mechanisms are required to sustain the interventions and ensure Pakistan is on track for the global commitments. Mapping of the stakeholders to ensure alignment of the National and Provincial priorities in lieu of 18th amendments.	Very high	Very high
Health in all policies	Nutrition sensitive interventions must be made compulsory whilst designing any policy or programme in sectors other than health.	Very high	High
Leadership (LEAD3)	Adoption of the WHO Eastern Mediterranean Regional Framework for actions to minimize the burden of NCDs through health interventions and the promotion of healthy diets.	Very high	Very high
Leadership (LEAD3)	The Pakistan National Dietary Guidelines need to be analysed to establish the RDA criteria for Pakistan, required for administering nutritional labels and food processing firms and regulatory entities.	Very high	Very high
Leadership (LEAD4)	To create awareness in the community regarding obesity.	Very high	Very high
Leadership (LEAD4)	To implement robust preventive campaigns and programmes for public awareness about malnutrition and alternatives for healthy food.	Very high	High
Leadership (LEAD4)	Awareness of nutrition and healthy life choices is important to combat NCDs.	Very high	High
Leadership (LEAD4)	Nutrition specific legislation and strategic framework is required to engage multistakeholder response on the double burden of disease in Pakistan to reduce NCDs.	Very high	Very high
Monitoring (MONIT1)	To monitor the continuity of the initiatives and policies for the prevention of NCDs.	High	Very high
Monitoring (MONIT2)	To enable the use of telemedicine and obesity prevention services to create awareness on the prevention of obesity and to have a transdisciplinary approach in the management and follow-up of patients regarding nutritional advice.	Very high	Very high
Monitoring (MONIT4)	To regularly monitor the nutritional status of the population particularly vulnerable populations with the aim to prevent non-communicable diseases.	Very high	Very high
**Sri Lanka**			
Leadership (LEAD1)	A re-establishment of national level nutrition leadership is required.	Very high	High
Leadership (LEAD3)	To strengthen the implementation of food-based dietary guidelines.	Very high	High
Leadership (LEAD4)	To revisit existing NCD prevention plans and identify gaps.	Very high	High
Governance (GOVER2)	Establish a clear framework for using evidence in systematic manner for the development of food policies.	Very high	High
Monitoring (MONIT2)	Monitoring mechanisms to be strengthened/established.	Very high	High
Monitoring (MONIT4)	Continue national surveys (such as STEPS), establish efficient disease registries (ideally computerised), improve speed of death registration data releases, and analyse deaths data.	Very high	High
Platforms for interaction (PLATF1)	To strengthen existing committees and councils.	Very high	High
Platforms for interaction (PLATF1)	To strengthen the Nutrition Steering Committee, chaired by the Secretary of Health, and the Technical Advisory Committees, chaired by the Director General.	Very high	High
Platforms for interaction (PLATF1)	To appoint a permanent authority focused on the prevention of NCDs which coordinates different stakeholders.	Very high	High
Platforms for interaction (PLATF1)	For the government to establish a taskforce or coordinating body to be chaired by a president to facilitate coordination across different ministries.	Very high	High
Platforms for interaction (PLATF2)	To have a coordinating body which enables formal platforms between the government and the commercial food sector to implement healthy food policies.	Very high	High
Platforms for interaction (PLATF3)	To have a coordinating body which enables formal platforms for the regular interaction between government and civil society on food policies and other strategies to improve population nutrition.	Very high	High
Platforms for interaction (PLATF4)	To have a coordinating body which enables the government to lead a broad, coherent, effective, integrated, and sustainable systems-based approach with local organizations to improve the healthiness of food environments and a national level.	Very high	High

#### Policy actions

Stakeholders identified a total of 118 recommended actions for policy. Pakistan had the highest number of policy recommendations (n = 44), followed by India (n = 30), Bangladesh (n = 23) and Sri Lanka (n = 21) (Supplementary Material 10).

Most recommendations (76%) were rated with high or very high importance, 18% were rated as medium–high importance, 4% as medium importance, and only 2% as low importance. In terms of achievability, only 36% of the policy recommendations were rated as high or very high achievability, 21% were rated as medium–high achievability, 22% as medium achievability, and 21% as very low, low, and low-medium achievability.

The very highly recommended actions in terms of both importance and achievability recognized by all countries were related to *prices*, *promotion, retail, and labelling*. Specifically, *food price* actions were discussed as fiscal policies, in the way of subsidies for healthy foods and taxation for unhealthy food and beverage (Table 4). Actions related to *food promotion* were discussed in the terms of banning/restricting unhealthy food advertisements especially among children and adolescents such as through posters or flyers within schools (Table 4). This also included policies aimed at regulating unhealthy food items sponsored by food industry, such as banning of toys associated with the acquisition of unhealthy foods. This further extends to retail recommended actions, which were discussed in terms of implementing support systems to encourage supermarkets to promote in-store availability of healthy foods, and to regulate the sale of unhealthy foods around schools. In terms of *food labelling*, all four countries prioritized introducing front of pack labelling to strengthen consumer choice (e.g., traffic light system). In addition, Pakistan and India recommended with very high importance actions related to provision, namely by raising the awareness of NCDs among teachers, students, and parents through school-based awareness programs, and establishing food programmes that provide food for the population at low cost or free (Table 4).

The recommendations rated with medium and low importance were related to menu labelling in quick-service restaurants (indicated by Bangladesh and Sri Lanka); retail policies such as encouraging the opening of food chains using more organic ingredients, the establishment of separate government body to monitor and develop protocols and guidelines for food retailers to promote healthy foods and limit availability of unhealthy (indicated by Sri Lanka, India, and Pakistan), and composition policies such as ensuring healthy food provided by catering companies (indicated by Pakistan).

Whilst all countries ranked each of the prioritised policy actions similarly in importance, there was some variance in perceived achievability of each country to reach those actions (Supplementary Material 10). For example, the four countries indicated the need for zoning laws (RETAIL1) for unhealthy food around schools as very high importance, but it was rated as very low achievability by Pakistan, low-medium by Bangladesh, medium by Sri Lanka, and high by India (Table 4).

For the four countries, the following will be more challenging in terms of achievability: monitoring the healthiness of processed foods, imposing high tax on unhealthy foods and fiscal incentives, banning the promotion of unhealthy foods in the print, mass, and social media, actions towards motivation of private sector producers and small shops (Supplementary Material 10).

#### Infrastructure support actions

A total of 82 *infrastructure support* recommendations across the four countries. A detailed description of these recommendations is available in Supplementary Material 10. For infrastructure support actions, Sri Lanka had the highest number of recommendations (n = 31) followed by Pakistan (n = 21), Bangladesh (n = 20) and India (n = 10) (Supplementary Material 10).

Ninety four percent of the recommended actions within the infrastructure support domain were ranked with high or very high importance, and 6% were ranked as medium importance (Supplementary Material 10). However, only 52% of the recommended actions were rated as high or very high in achievability, 34% were rated as medium achievability, and 14% as low achievability.

The most highly recommended actions in terms of both importance and achievability by all countries are related to *monitoring*, *platforms*, and *leaderships* (Table 5). In terms of *monitoring*, recommendations focused on improving/strengthening the monitoring and surveillance mechanisms to regularly monitor the health status of the population and food environments, as well as implementing regular assessments on the impact of regulations that may control the unhealthy food environments such as taxation or limiting promotion of unhealthy foods in schools/academic institution (Fig. 4 and Supplementary Material 10). In terms of *platform*s, the four countries highlighted the need to strengthen coordination among various stakeholders and interorganizational collaboration for enforcing existing policies and the need to appoint/establish an authority to coordinate among stakeholders. The other area of focus was *leadership*—specifically the prioritisation of government initiatives on the prevention of diet related NCDs, and coordination across departments and levels of government, industry, and civil society (Table 5 and Supplementary Material 10).

Less likely to be recommended were actions related to *governance,* with only Pakistan and Sri Lanka indicating the need for a clear framework to use evidence in systematic manner for the development of food policies, and accountability mechanisms to sustain interventions and Global Commitments (Supplementary Material 10).

Further, the actions rated as medium importance were related to health in all policies, such as agricultural policies to be made nutrition sensitive and inclusive of climate change factors (indicated by India) and the inclusion of nutrition related indicators in all social protection programs (indicated by Bangladesh).

In terms of achievability, countries found the following challenging: the implementation and enforcement of policies, the monitoring of industries if they are following guidelines, and the allocation of adequate resources (human and monetary) to improve Food-EPI programs (Supplementary Material 10).

## Discussion

Utilising the validated Food-EPI tool, our study mapped food policies and infrastructure support in Bangladesh, India, Pakistan, and Sri Lanka, to identify strategic priority actions for creating healthier food environments to mitigate diet related NCDs. Our findings, indicate policies and programmes in the participating South Asian countries emphasized hygiene and food safety, including the enforcement of hygiene and adulteration regulations over interventions aimed at NCD prevention. Results show low level of policy and support infrastructure implementation for the prevention of diet related NCDs, notwithstanding regular NCD surveillance across these countries. Additionally, we observed a lack of policies and supporting infrastructure to discourage (encourage) unhealthy (healthy) food availability and promotion.

Our findings align with those from other low- and middle-income countries (LMICs).^27^, ^28^, ^29^ For instance, in Malaysia,^27^ more than half of the Food-EPI indicators showed weak implementation, a trend consistent with our observations. Predominantly, indicators addressing policies which incentivise healthier food retailers and promote the availability of healthier food options were weakly implemented in Malaysia^27^ and Thailand^28^ which is consistent with this study. Similarly, the infrastructure support indicators related to routine surveillance checks and the establishment of food safety regulations were rated the highest, mirroring trends in Thailand,^28^ Malaysia,^27^ and Ghana.^29^

Our findings also align with the NCD Progress Monitor 2022,^18^ which highlights the need for regular NCD monitoring and governmental initiatives to ensure nutrient goals. A discrepancy was noted in India regarding the implementation of marketing restrictions on unhealthy food products to children, which was reported as fully implemented by WHO but rated as weak in our study. This difference may arise from the narrower definition used by the Food-EPI tool which asks specifically about media and school marketing restrictions of unhealthy foods whereas in the WHO report it is related to a set of 12 guidelines on food marketing.^18^

In light of these gaps, stakeholders recommended several priority actions, including the introduction of front-of-pack labelling, the establishment of regulatory frameworks for food promotion, and setting food provision standards in schools. These actions align with recommendations from Malaysia and Thailand, emphasizing the importance of subsidies on fruits and vegetables, food taxation, and comprehensive surveillance systems.^27^^,^^28^ This recommendation aligns with the Food Agriculture Organization (FAO) who in their report titled *The state of food security and nutrition in the world**^30^* highlights healthy food subsidies as the best way forward to provide the largest improvement in the affordability of healthier diets. In addition, stakeholders highlighted the importance of high fat, sugar and salt food taxation, composition, and surveillance systems which also overlap with recommendations from the WHO, which encourages governments to promote a package of actions comprising the taxation of unhealthy foods and beverages in combination with healthy food subsidies, the regulation of food marketing targeted towards children, the existence of clear and accurate nutrition labels, the reduction of salt, sugars and saturated fat in processed food and beverages, the availability of healthy foods in schools and other public places, improving surveillance and strengthening national food systems.^31^ Food procurement at the school level was highlighted as a key priority for healthier diets.^31^^,^^32^ School food programmes are opportunities to shift and stimulate food processing and food retailing practices towards healthier food supply, greater demand for healthy foods and making healthy food production more financially viable facilitating healthier dietary behaviours.^31^^,^^32^ Therefore, as observed in other countries,^33^ food provided through school food programmes could have an impact on healthier nutritional status in children and contribute to the development of healthy food preferences which can transform into healthier populations.

Finally, all participating countries recommended a health in all policies approach for targeting obesogenic food environments as being key to halt the rise in diabetes and obesity prevalence in these countries. Stakeholders suggested governments to move from the realm of traditional public health measures, which only target personal behaviour and have been proven to be ineffective, to a health in all policies approach encompassing comprehensive policies targeting food environments including regulations on the availability, affordability, and access to unhealthy food. This approach is aligned with the newest recommendations from the WHO^34^ as a recognition of the difficulties countries globally face in halting the rising levels of obesity and NCDs.

The potential for the reapplication of the Food-EPI tool to measure progress over time in South Asia holds significant value for evaluating the effectiveness of implemented policies and tracking advancements in creating healthier food environments. Utilizing this tool in subsequent assessments could provide a longitudinal perspective on policy changes and their impact on preventing diet-related non-communicable diseases. However, undertaking long-term monitoring presents several challenges that necessitate careful consideration. Financial resources emerge as a critical obstacle, as sustaining the assessment process over an extended period requires continuous funding. The financial challenge is particularly pronounced in resource-constrained settings, where competing health priorities may limit the allocation of funds to comprehensive policy evaluations. To ensure the sustainability of the assessment process, strategic planning and collaboration with international organizations, donor agencies, and local governments become imperative. Establishing partnerships and securing dedicated funding streams will be crucial for overcoming financial barriers and maintaining the continuity of the Food-EPI assessments, thereby enabling a robust and consistent monitoring mechanism for assessing policy impact on creating healthier food environments in South Asia.

Importantly, our study does not assess challenges around development and implementation of policies and associated support infrastructure that can explain the gaps identified in these analyses. Future research should focus on the views of a broader set of stakeholders including those of various sectors in government particularly focusing on representatives from the financial sector within government, such as economic advisors, budget analysts, and treasury officials. This approach would provide a more comprehensive understanding of the economic and political trade-offs that may impede the progress and execution of these policies.

There are several caveats to our analyses. Adjustments in workshop procedures were required to adhere to pandemic mitigation strategies. These modifications potentially introduced minor dissimilarities in the execution of the study across different locations. In addition, challenges related to stakeholder engagement were notable, particularly related to the availability and time commitment required from participants. The reduced attendance of policymakers and NGO representatives in some countries, further compounded by the constraints of the COVID-19 pandemic and political events, implied a shift to online workshops to maintain stakeholder engagement.

Regarding panel composition within each country, Bangladesh and India featured a higher number of non-government stakeholders compared to government stakeholders whilst Sri Lanka and Pakistan had a good balance between the two types of stakeholders. This variability in representation could have influenced implementation scores, introduced variability into the consensus building and, potentially, skewed recommendations towards the perspectives of a specific stakeholder group. Our findings suggest policymakers tended to rate the effectiveness of policies differently than their non-government counterparts. Whilst small sample sizes precluded an assessment on whether these differences were statistically meaningful, we can't rule out a potential bias linked to policymakers' roles and expectations, possibly influencing their perceptions of policy success. For example, both Sri Lanka and Pakistan proposed actions for Governance. It's possible that the presence of a larger number of intergovernmental stakeholders (e.g., WHO and UNICEF) in these countries, compared to India and Bangladesh, influenced these recommendations. Additionally, the variation in panel composition appears to have impacted other policy recommendations. For example, whilst all countries supported the idea of developing zoning laws around schools, Indian stakeholders, who had a higher NGO representation, deemed these laws highly achievable. Conversely, in Sri Lanka, the implementation of similar zoning laws was considered moderately achievable, which may reflect the substantial involvement of both NGOs and intergovernmental organizations in the stakeholder mix. This contrasts with Pakistan, where stakeholders, in the absence of a similarly robust representation, perceived the achievability of zoning laws as very low, a sentiment echoed to a lesser extent by Bangladeshi stakeholders, who rated it as low to medium. Another example includes the notable volume of recommendations aimed at enhancing infrastructure support in Sri Lanka and Pakistan, which may be linked to a greater presence of government stakeholders relative to those in Bangladesh and India. Overall, these discrepancies underscore a divergence in viewpoints on policy effectiveness and priorities between those within government and external actors. The observed discrepancies between government and non-government stakeholders' perceptions hint at potential underlying variations in their priorities, resource allocation, and perceived impacts of policies.

Whilst we can't rule out country differences in results are not driven by panel composition heterogeneity, the consistency across stakeholder assessments, demonstrated by a high IRR, mitigated to some extent this caveat. Furthermore, the study's recommendations were derived from extensive consensus-building among stakeholders, with various strategies employed to ensure a comprehensive exchange of views, including facilitated discussions and breakout rooms, thus minimizing biases potentially arising from unequal representation. These collaborative efforts culminated in actionable recommendations that reflected a wide spectrum of stakeholder insights, emphasizing the importance of inclusive, participatory processes in policy evaluation and development.

In addition, biases in stakeholder ratings were another potential limitation, possibly affected by recall period, selective recall, or social desirability or the subjectivity into the assessment of policy implementation. To enhance the validity and reliability of our measurements, we adopted a multi-faceted approach. We employed a validated survey instrument with precise questions to reduce recall bias. Efforts were also made to establish trust with stakeholders, fostering an environment for honest feedback and reducing social desirability bias. Recognizing that the ranking of actions could be influenced by subjective judgments, we facilitated a guided discussion among a diverse group of stakeholders during the workshop. This strategy aimed to incorporate a wide range of viewpoints and to clarify the criteria for action prioritization, ensuring a balanced and transparent decision-making process.

Furthermore, the Likert scale's design inherently relied on individual interpretations of policy strength, making it susceptible to subjective judgments. Stakeholders' diverse perspectives and expertise levels may have led to varying interpretations of the same policy indicators, underscoring the need for careful interpretation of the results. The assessment by different national stakeholder panels also raised concerns about the comparability of results, with some panels potentially being more critical than others. Additionally, the study's findings and policy implications are specific to the four South Asian countries examined and may not be generalizable to other regions.

Another limitation of our study is the complex and multifaceted nature of NCDs aetiology. Whilst food environments and policies are crucial for improving diets, factors like genetic predispositions and socio-cultural influences also significantly impact NCDs and may necessitate interventions beyond the scope of our analyses. Consequently, our study did not comprehensively evaluate the applicability and implementation of global best practices in diverse cultural and economic contexts. This underscores the importance of future research aimed at developing more tailored and context-specific strategies.

Lastly, the timing of data collection until December 2021 and the potential for policy changes may involve limitations. However, whilst policy environments can evolve, the slow nature of policy development and implementation suggests the study's findings remain relevant.

Despite its caveats, the study boasts several strengths. The Food-EPI tool provides a robust framework for evaluating upstream policies and infrastructure support influencing food environments and dietary choices.^20^ The consultation process with experts offered valuable insights into policy actions and gaps, contributing to the identification of feasible and impactful policy actions for South Asia. The engagement of a diverse array of stakeholders, spanning diverse backgrounds (gender, education, and expertise) offers a multifaceted view of food policy and infrastructure support evaluation across Bangladesh, India, Pakistan, and Sri Lanka.

Being the first application of the Food-EPI tool in the participating countries, the study findings offer a baseline for future assessments to measure progress. Additionally, the creation of an evidence document for each country serves as a valuable resource for both government and non-government sectors to review policy gaps and formulate strategic change in policy landscapes. Our findings underline the critical need for targeted policies to combat obesity and diet-related NCDs, extending beyond the current focus on food safety and hygiene. It suggests prioritizing the enhancement of food labelling, the introduction of fiscal policies to promote healthy eating, the implementation of stricter regulations on food promotions, and the improvement of nutritional standards in schools. Effective policy implementation requires cross-sectoral collaboration, adaptations to fit local cultural contexts, and active engagement with stakeholders to ensure broad acceptance and success. For policymakers, addressing these areas with comprehensive strategies and ensuring the necessary support and resources are allocated is essential for the significant improvement of public health outcomes in the region.

## Contributors

Elisa Pineda: Stakeholder workshop design and execution, data curation, formal analysis, investigation, methodology, project administration, validation, visualization, writing–original draft, writing–review & editing.

Petya Atanasova: Analysis, writing–review & editing.

Nalinda Tharanga Wellappuli: Project administration, analysis, writing–review & editing.

Dian Kusuma: Conceptualization, project administration, investigation, analysis, writing–review & editing.

Himali Herath: Project administration, investigation, analysis, writing–review & editing.

Alexa Blair Segal: Writing–review & editing.

Stefanie Vandevijvere: Stakeholder workshop design, methodology, validation, visualization, writing–review & editing.

Garudam Raveendiran Aarthi: Data curation, investigation, writing–review & editing.

Saira Afzal: Data curation, investigation, writing–review & editing.

Abu Ahmed Shamim: Data curation, investigation, writing–review & editing.

Fahmida Akter: Data curation, investigation, writing–review & editing.

Ranjit Mohan Anjana: Data curation, investigation, writing–review & editing.

Faiza Aziz: Data curation, investigation, writing–review & editing.

Ananya Gupta: Data curation, investigation, writing–review & editing.

Abu Abdullah Hanif: Data curation, investigation, writing–review & editing.

Mehedi Hasan: Data curation, investigation, writing–review & editing.

Renuka Jayatissa: Data curation, investigation, writing–review & editing.

Sujeet Jha: Data curation, investigation, writing–review & editing.

Vinitaa Jha: Data curation, investigation, writing–review & editing.

Prasad Katulanda: Data curation, investigation, writing–review & editing.

Khadija Irfan Khawaja: Data curation, investigation, writing–review & editing.

Balachandran Kumarendran: Data curation, investigation, writing–review & editing.

Menka Loomba: Data curation, investigation, writing–review & editing.

Sara Mahmood: Data curation, investigation, writing–review & editing.

Malay Kanthi Mridha: Data curation, investigation, writing–review & editing.

Rajendra Pradeepa: Data curation, investigation, writing–review & editing.

Akansha Tyagi: Data curation, investigation, writing–review & editing.

Anuradhani Kasturiratne: Data curation, project administration, investigation, writing–review & editing.

Franco Sassi: Conceptualization, funding acquisition.

Marisa Miraldo: Conceptualization, funding acquisition, methodology, investigation, formal analysis, project administrator, supervision, validation, visualization, writing–review & editing.

## Data sharing statement

Data collected for this study, including pseudo anonymised participant data and a data dictionary is available in the Supplementary Material.

## Declaration of interests

We declare that we have no conflicts of interest.
