# Supplementary material for: Policy implementation and recommended actions to create healthy food environments using the Healthy Food Environment Policy Index (Food-EPI): a comparative analysis in South Asia
Source: Lancet Reg Health Southeast Asia. 2024 Jun 26;26:100428. doi: 10.1016/j.lansea.2024.100428 (PMC11260855; doi:10.1016/j.lansea.2024.100428)
Supplement: Supplementary Material [file mmc1.pdf]

Supplementary Material

Policy implementation and recommended actions to create healthy food environments using the Healthy Food Environment Policy Index (Food-EPI): a pooled level analysis in South Asia

Contents

Supplementary material 1 – PRISMA flow diagram for the policy review 2

Supplementary Material 2 – Methods 3

Supplementary Material 3 – Food-EPI Indicators and domains – Policies 9

Supplementary material 4 – Food-EPI Indicators and domains – Infrastructure support 11

Supplementary material 5 - Questions for rating workshop 13

Supplementary material 6 – Policy evidence document- Food environment policy index (Food-EPI) – Bangladesh 14

Supplementary material 7 - Policy evidence document- Food environment policy index (Food-EPI) – India 21

Supplementary material 8 – Policy evidence document – Food environment policy index (Food-EPI) – Pakistan 30

Supplementary material 9 - Policy evidence document- Food environment policy index (Food-EPI) – Sri Lanka 36

Supplementary material 10 – Recommended actions by stakeholders 44

References 55

Supplementary material 1 – PRISMA flow diagram for the policy review

Figure S1: PRISMA flow diagram for the policy review in Sri Lanka

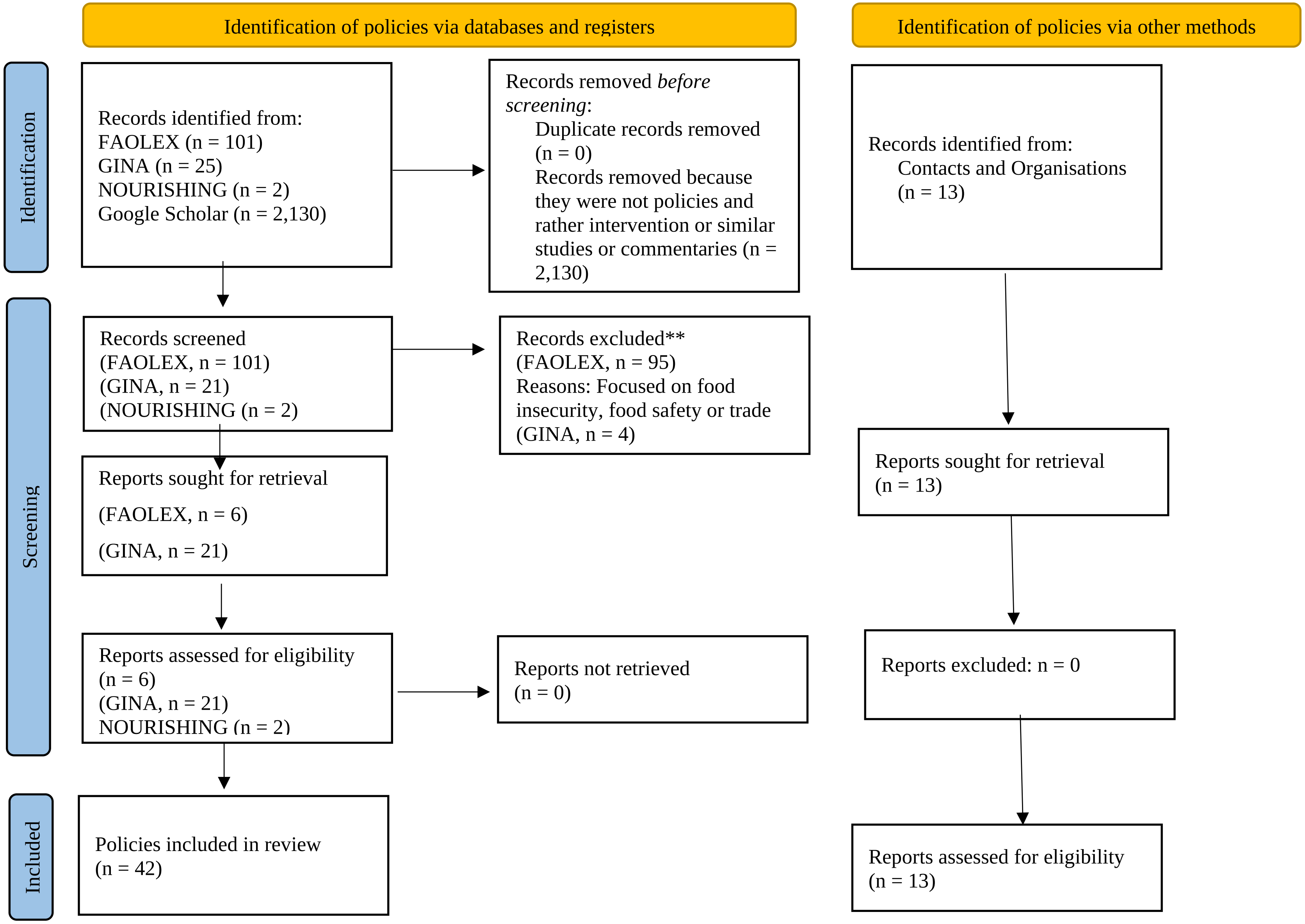

Supplementary Material 2 – **Methods**

First, we mapped and reviewed the status of the implementation of food policies in the participating South Asian countries up until December 2021 (Policy mapping review). Policy mapping, as indicated by Raboy and Padovani[1], was carried out through the collection and organization of data concerning related policies for the prevention of obesity and non-communicable diseases using the Healthy Food Environment Policy Index (Food-EPI) framework. Sources that were searched to collect the policies and infrastructure support evidence included direct government contacts and government websites, the NOURISHING database, and The Global database on the Implementation of Nutrition Action (GINA).[2] The identified policies were selected based on their ability to enable healthy food environments for the prevention of obesity and diet-related Non-Communicable Diseases (NCDs). Second, we carried out a rating assessment of the strength in terms of level of implementation of policies and ranked their importance and feasibility stakeholder workshops (Policy rating assessment). Third, and identification of government priority actions was generated by stakeholders (Priority action recommendations). The output of this process was a report card, showing the evaluation results and recommended actions for each of the analysed countries.

Food-EPI framework rational and description

To assess the implementation of food policies we used the Healthy Food Environment Policy Index (Food-EPI) international validated tool developed by the International Network for Food and Obesity/NCDs Research, Monitoring and Action Support (INFORMAS).[3] This Food-EPI enables the identification and assessment of the extent of implementation of recommended food environment policies by governments compared with international best practices and prioritizes actions to fill implementation gaps.[3]

The Food-EPI tool encompasses two components 1) policies and 2) infrastructure support with seven domains each. We focused on assessing policies targeting the Food EPI domains of Food composition, Food labelling, Food promotion, Food prices, Food provision, Food retail, Leadership, Governance, Monitoring and intelligence, Funding and resources, Platforms for interaction, Workforce development, and Health in all policies. A detailed explanation of the indicators of each domain can be found in Table S1 and Table S2.

The selection of the Food-EPI as our monitoring framework is underpinned by its unique attributes and strategic significance.[4] Unlike alternative frameworks, the Food-EPI is purposefully designed to assess the extent of implementation of food environment policies. This focus on evaluating governmental progress in fostering healthy food environments and implementing obesity and NCD prevention policies is distinctive and pivotal.[5]

The Food-EPI serves as a comprehensive tool, providing a systematic approach to monitor, benchmark, and support government actions in creating healthier food environments.[4] Its development process involved a review of policy documents and consultation with international experts, ensuring its relevance and applicability across diverse contexts. By encompassing various domains associated with good practice indicators and benchmarks, the Food-EPI allows for a nuanced evaluation of policy implementation.[5]

The tool's dynamic nature sets it apart, enabling repeated assessments over time. This feature supports longitudinal analysis, offering insights into changes, improvements, or challenges in food policy implementation. The Food-EPI's ability to track and assess progress fosters accountability, encourages continuous improvement, and aligns with global health strategies such as the World Health Organization's Global Action Plan for the Prevention and Control of NCDs.[6]

The Food-EPI has proven to be an influential tool in shaping food policy decisions across various countries by providing a framework to evaluate and improve food environments.[7] In Malaysia, a 2018 report utilized the Food-EPI to benchmark existing policies and set future directions, showcasing its value in policy evaluation and planning[8] Similarly, a New Zealand study highlighted its utility in tracking policy implementation and identifying actionable areas within food environments from 2014 to 2017.[9] Thailand's assessment in 2017 demonstrated the Food-EPI's versatility by evaluating policy implementation levels involving both state and non-state actors.[10] Australia's project team in 2017 and 2020 leveraged the Food-EPI to develop a scorecard and priority recommendations, targeting obesity and advocating for healthier food environments.[11] Additionally, a 2017 Canadian report underscored the Food-EPI's role in summarizing national efforts and guiding policy priorities towards healthier food environments.[12] These examples underline the Food-EPI's critical role in informing and guiding policy decisions aimed at improving public health nutrition through enhanced food environments, by benchmarking against best practices and identifying priority areas for action.

Policy identification framework for policy mapping

To identify relevant policies, as guidance, we used a systematic literature review method using elements of the Preferred Reporting Items for Systematic reviews and Meta-Analyses (PRISMA) framework and checklist. The research question which guided the policy review was: *What food policies for the prevention of obesity and non-communicable disease have been implemented in [Bangladesh, India, Pakistan Sri Lanka] up to the year 2021?* Food policies were specifically based on the 47 practice indicators from the Food-EPI domains indicated above which encompassed: food composition; food labelling; food promotion; Food prices; Food provision Food retail; Health in all policies (Figure S2). Therefore, the aim of this policy review was to identify specific food related policies in Sri Lanka that have implemented for the prevention of obesity and non-communicable diseases.

Policy context analysis

The South Asian research teams reviewed the indicators of the INFORMAS Food EPI tool.[13] The indicators were selected based on the relevance of the assessed South Asian countries and the remit of our research aim regarding the identification of relevant policies and interventions that are in place that can inform the design of population-based interventions. Revision of indicators was undertaken by a local team of researchers and an independent researcher conducted a reliability check on all indicators. Ethical approval for the project was obtained in each of the participating countries.

Policy mapping review

Inclusion and exclusion criteria

For each of the 47 good practice indicators, evidence for the existence and degree of implementation of policies was collected by a team of researchers, through a systematic policy search. The search strategy was based on the population, intervention, comparison, and outcome (PICO) framework. In terms of population, policies focusing on any type of population (e.g., adults, children) were included. For intervention, only policies, legislations or established programmes focusing on nutrition, health, food, or the food environment were included whilst scientific interventions and proposals were excluded. Control was irrelevant for this search. Regarding the outcome, selected policies targeted overnutrition, obesity or any form of non-communicable disease (e.g., type 2 diabetes, cardiovascular disease). Policies that focused on food security, undernutrition, food safety were excluded.

Two researchers were involved in the collection of data in each country and independently assessed each of the policies considering the PICO inclusion and exclusion criteria. Upon completion of the data collection, data was extracted and shared with a third independent researcher, in accordance to the Cochrane Handbook for Systematic Reviews.[14]

Figure S2: Components and domains of the Healthy Food Environment Policy Index (Food-EPI)

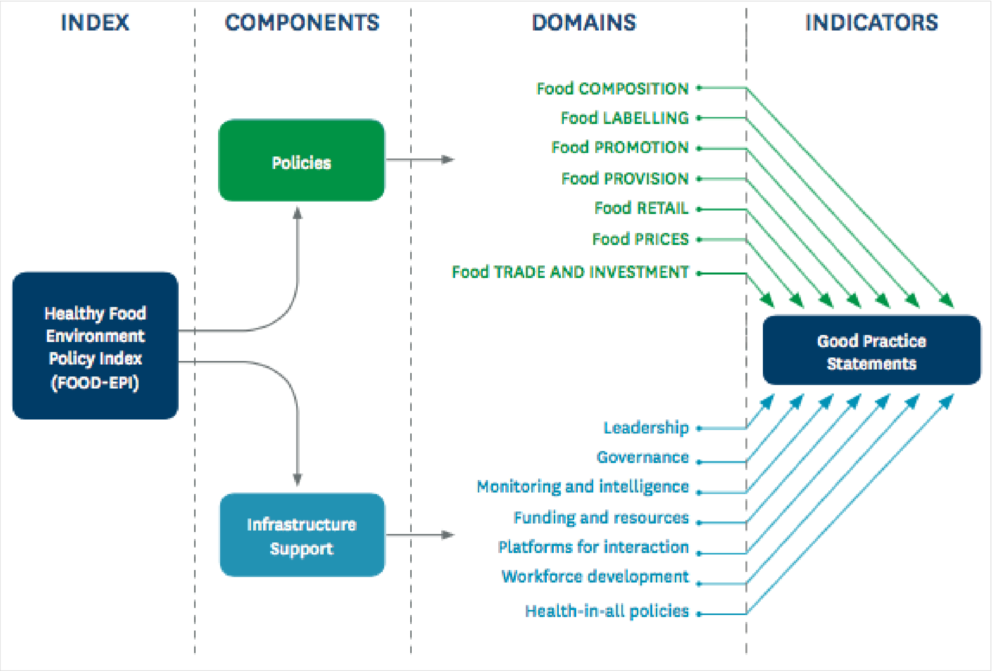

Source: INFORMAS, 2017

Data sources

Policy searches were undertaken via the Global database on the Implementation of Nutrition Action (GINA), the World Cancer Research Fund International NOURISHING framework, FAOLEX, the Google Scholar database, and national government websites. Databases were searched from the beginning of existence up to December 2020.

Search strategy

The search strategy was designed in consultation with a specialist in systematic reviews from Imperial College London.

Search terms were developed from previous literature and the key terms (indicators) provided in the Food-EPI tool[3] for the topics of 1) the food environment, 2) obesity and 3) Policy In addition, the snowballing method was used to include relevant policy documents, plans, strategies and briefs, policies as recommended by policy experts in the region (Figure S1). Policies that did not comply with a focus on obesity or NCD and focused instead on food safety or food security or the document was not a policy, regulation or legislation were the identified reasons of exclusion. Figure S1 presents the search strategy for Sri Lanka.

Data collection and extraction framework and coding

In accordance with the Cochrane handbook to undertake systematic reviews, using a priory theory and a predetermined framework we extracted data from selected policies.[14] The Food-EPI tool[13] was used as a code frame due to the inclusion of a hierarchical set of relevant and previously validated themes indicated in Tables S1 and S2 and the Policy evidence document. The coding framework was previously developed and validated by the INFORMAS network.[13]

In accordance with the framework, for each of the identified policies, the following elements were extracted: the policy title, its evidence of implementation, the content of the policy intervention, the type of population that the policy was intended for, the date and place of the policy implementation and how the policy is being implemented and the source when the policy was obtained.

Two reviewers collected the data from each policy who worked in coordination and a third reviewer revised the collection of the policies and the extraction of the data from each policy. Upon a disagreement this was discussed and agreed upon the reviewers.

Collection of evidence and development of a policy evidence document

All identified policies were summarized into an evidence document. This document was compiled in 2019-2020 and summarized policy actions implemented in Bangladesh, India, Pakistan, and Sri Lanka regarding the food environment up until December 2020. The policy evidence document also included international best practice examples of each policy, and infrastructure support domain to have a comparison standard for each domain. International best practice policy examples were selected.[13] ‘Best practice exemplars’ or benchmarks were the tools through which health promoting environments were created and assessed. They are comprehensive examples of policy implementation worldwide and were chosen based on their strength (e.g. external validated measures such as using independent nutrient profiling criteria), and comprehensiveness (e.g. including a broad range of age groups, food groups, physical activity measures, media, settings, or regions), and evaluation of the impact of public and private sector policies on food environments is needed to strengthen accountability systems to reduce NCDs.

The validation of policy evidence involved a systematic process wherein a document containing identified policies was shared with government officials via email. Officials were requested to meticulously review the document and provide feedback regarding any potential errors, omissions, or inaccuracies in the included policies. This collaborative approach aimed to ensure the accuracy and completeness of the policy evidence by leveraging the expertise of government officials directly involved in public health policy. The engagement with government authorities through verbal communication and written correspondence facilitated a thorough review, allowing for the identification and rectification of any discrepancies. This method served as a robust mechanism to validate the accuracy and relevance of the policies included in the study, enhancing the overall reliability of the policy evidence used for the assessment of food environment policies in South Asia.

In the Policy Evidence document we considered policies that were under development or had already terminated to have a general overview of the policies that had been implemented for each Food-EPI indicator in each country. However, during the rating workshop, we asked stakeholders to only rate the implementation level of policies that were currently implemented.

Policy rating assessment

Stakeholder engagement

Workshops were undertaken online due to COVID-19 pandemic restrictions on travel and social distancing. As undertaken by similar studies,[15] a heterogeneous purposive sample method was used to recruit experts from the key sectors of academia, government, and non-profit organizations.

Stakeholder inclusion criteria encompassed having an expertise in public health, nutrition, and/or public health policy, living in the country where the policy assessment was being undertaken, and being a relevant actor in the academic, government/government agencies, and/or non-profit sector. It was necessary that stakeholders had a scientific understanding of nutrition and food related policies and/or obesity and NCD outcomes to understand and be able to evaluate the food policies and generate according to government recommendations. Representation of various sectors was important to ensure different perspectives and account for information bias when evaluating the food polices.

In terms of exclusion criteria, stakeholders from the food industry were excluded which was justified by the inherent conflict of interest that may arise, given the industry's primary focus on profit motives and potential misalignment with public health objectives. In engaging stakeholders without conflicts of interest, the study aims to prioritize unbiased input and maintain the integrity of the policy assessment process. Therefore, this exclusion ensures a more impartial evaluation.

The transition to online workshops due to the COVID-19 pandemic brought about significant changes in stakeholder interactions, consensus-building, and data collection dynamics. The virtual format altered communication modes and potentially impacted participant engagement and the richness of discussions. Achieving consensus in the absence of in-person cues posed challenges, necessitating careful consideration of the nuances introduced by the online setting. Despite these challenges, efforts were made to enhance online engagement through clear instructions and interactive tools. The discussion in the revised manuscript delves into these nuances, providing a concise yet thorough exploration of how the shift to online workshops may have influenced the study's outcomes and ensuring transparency in addressing the implications of the virtual format on data quality.

The utilization of a purposive sample method for stakeholder selection introduces the potential for bias, particularly when certain perspectives or sectors are either overrepresented or underrepresented. In the case of the South Asian workshops, the underrepresentation of NGOs and policymakers, coupled with the overrepresentation of academics and researchers, may result in a skewed sample that may not fully capture the diverse viewpoints crucial for comprehensive policy assessment. This bias could impact the study findings, as the identified priorities and recommended actions might predominantly reflect the perspectives of academics rather than those actively engaged in policy formulation and implementation. Given that the study's findings emphasize the need to update and improve policies to address rising levels of obesity and non-communicable diseases, the underrepresentation of key stakeholders may limit the study's ability to provide well-rounded and actionable recommendations that account for the practical challenges and considerations faced by policymakers and NGOs in implementing effective strategies to combat these health issues.

Whilst the rating considering seven policy domains and seven infrastructure support domains facilitates a systematic and standardized evaluation of food environments, the approach may have limitations by potentially overlooking critical aspects tied to cultural contexts, socioeconomic factors, and regional variations. This acknowledgment emphasizes the need for future research to explore these additional dimensions to enhance our understanding of the complexities within food environments.

Actor mapping was based on the Actor and Policy mapping tool from the NewClimate Institute.[16] This tool allowed the systematic mapping of relevant stakeholders and policies the sectors of government, academia and NGOs in Bangladesh, India, Pakistan, and Sri Lanka. The collected information allowed a visualisation of the interactions between institutions and different stakeholders. The stakeholder analysis was carried out using the Actor and Policy framework from NewClimate, which included the collection of the expert's name, type of actor, expertise, their organization, description of role and level and area of influence, description of position and core interests and contact details were collected. Stakeholders that met criteria and had no conflict of interests were invited to a rating workshop as the national expert group.

Experts were invited to attend an online rating workshop via email in which the policy evidence document was explained and provided. The rating workshop was based on the Delphi method to approach an expert consensus on 1) the evaluation of the food policies, 2) relevant recommendations for government and 3) to determine their importance and feasibility of recommended actions.

Delphi Method

The Delphi method was employed in three rounds to facilitate stakeholder engagement in the assessment of food policies and infrastructure support. In the first round, stakeholders, including representatives from government, academia, and non-profit sectors, individually ranked the implementation level of each policy and infrastructure support indicator using a Likert scale. The second round involved the generation of recommended actions by stakeholders, who then ranked these actions based on their perceived importance and feasibility. Criteria for achieving consensus were established through a round table of discussions and voting on the ranking of each indicator. In instances where consensus was not initially reached, iterations were conducted to allow stakeholders to reconsider their positions and engage in further discussions. This structured and iterative Delphi process provided a systematic framework for stakeholder involvement, ensuring a comprehensive exploration of diverse perspectives and enhancing the understanding of the stakeholder engagement process in prioritizing policy actions.

The use of the Delphi method in stakeholder assessment, whilst potentially introducing subjectivity and bias, has been carefully addressed in our study. We acknowledge the diversity of stakeholder opinions and the limitations of consensus-based methods. However, our approach, incorporating a diverse group of stakeholders and employing a heterogeneous purposive sampling method, aims to mitigate these concerns. The Delphi method's iterative and anonymous nature encourages the expression of individual perspectives, preventing dominant voices from overshadowing minority viewpoints. This comprehensive strategy enhances the robustness of our stakeholder assessment within both government and non-governmental sectors, ensuring a balanced and inclusive representation of diverse perspectives.

Rating workshop

In the rating workshop, experts were first instructed to assess the implementation level of each policy and infrastructure support indicators using a five-point Likert scale and considering the provided international best-practice policy examples from the compiled policy evidence document. Each of the survey questions may be found in the Questions for rating workshop section. To facilitate evaluation, a presentation on the relevant indicator, its definition, a best practice example and the relevant identified country policies was provided. The expert panel rated the level of implementation according to the compiled policy evidence document, their expertise and professional perspective of existing food environment related policies as '1' (indicating non-existence) to '5' (representing very strong implementation), A 'cannot rate' option was also included.

Secondly, the concerted evaluation results were presented to the expert panel and a discussion was guided to identify gaps and potential government recommended actions. Experts in each participating country were asked to formulate recommended actions for the government based on the policy and infrastructure support domains that, in turn, would improve food environments in Bangladesh, India, Pakistan, and Sri Lanka.

The Likert scale employed for stakeholder ratings may introduce a subjective element to the assessment of policy implementation. Despite efforts to provide relevant information, the scale's design inherently relies on individual interpretations of policy strength, making it susceptible to subjective judgments. This subjective nature arises from diverse perspectives and varying levels of expertise among stakeholders, potentially leading to differing interpretations of the same policy indicators. Whilst the scale offers a structured framework for evaluation, the inherent subjectivity emphasizes the need for cautious interpretation of the results, recognizing that individual perceptions of policy strength may differ, influencing the overall assessment of the policies aimed at creating healthier food environments in South Asia.

Priority action recommendations

Action identification, prioritization, and achievability

The third stage of the workshop concerned the prioritisation of the identified recommended actions to create healthy food environments in Bangladesh, India, Pakistan, and Sri Lanka. Actors were asked to prioritise the recommended actions by ranking the policy and infrastructure support actions on relative importance, and feasibility. Experts received instructions for how to rank the actions based on importance and feasibility. The importance and feasibility criteria they used when ranking the actions can be found in Table S1. Experts ranked the policy actions two times on a provided scale: first on importance, second on feasibility. In addition, experts ranked recommend actions in terms of feasibility considering the scale of feasible in the short term (1-2 years), the medium term (2-5 years) or long term (5-10 years). To identify the recommended actions with the highest priority, we calculated the sum of the scores (rankings of all experts) for each action. The scores for importance and achievability were first calculated separately. Second, the total score for each action was calculated

considering the scores on both importance and feasibility. The latter was used to determine the final ranking of policy and infrastructure support actions. The lower the sum score, the higher the action was ranked by the experts.

Data analysis

An agreement score across the different types of stakeholders was calculated considering the mean score on each indicator and used to determine the level of policy implementation with respect to the 43 policy and infrastructure support indicators. To test response rate differences among stakeholders when rating the various policy and infrastructure support domains, the interrater response rate (IRR) was assessed through a weighted analysis, using Gwet's AC_2 coefficient.

Supplementary Material 3 – Food-EPI Indicators and domains – Policies

Table S1: Food-EPI Indicators and domains – Policies

Domains	Proposed good practice	Indicators
Food composition	There are government systems implemented to ensure that, where practicable, processed foods minimise the energy density and the nutrients of concern (salt, fat, saturated fat, trans fat, added sugar).	Food composition targets/standards have been established for processed foods by the government for the content of the nutrients of concern in certain foods or food groups if they are major contributors to population intakes of these nutrients of concern (trans fats and added sugars in processed foods, salt in bread, saturated fat in commercial frying fats).
		Food composition targets/standards have been established for out-of- home meals in food service outlets by the government for the content of the nutrients of concern in certain foods or food groups if they are major contributors to population intakes of these nutrients of concern (trans fats, added sugars, salt and saturated fat).
Food labelling	There is a regulatory system implemented by the government for consumer-oriented labelling on food and food menu boards in restaurants to enable consumers to easily make informed food choices and to prevent misleading claims.	Ingredient lists and nutrient declarations in line with Codex recommendations are present on the labels of all packaged foods.
		Robust, evidence-based regulatory systems are in place for approving/reviewing claims on foods, so that consumers are protected against unsubstantiated and misleading nutrition and health claims.
		A single, consistent, interpretive, evidence-informed front-of-pack supplementary nutrition information system, which readily allows consumers to assess a product's healthiness, is applied to all packaged foods.
		A consistent, single, simple, clearly visible system of labelling the menu boards of all quick service restaurants (i.e., fast food chains) is applied by the government, which allows consumers to interpret the nutrient quality and energy content of foods and meals on sale.
Food promotion	There is a comprehensive policy implemented by the government to increase the impact (exposure and power) of promotion of healthy food to reduce that of promotion of unhealthy foods including to children (<16years) across all media.	Effective policies are implemented by the government to restrict exposure and power of promotion of unhealthy foods including to children through broadcast media (TV, radio).
		Effective policies are implemented by the government to restrict exposure and power of promotion of unhealthy foods including to children through non-broadcast media (e.g., Internet, social media, food packaging, sponsorship, outdoor advertising including around schools).
		Effective policies are implemented by the government to ensure that unhealthy foods are not commercially promoted including to children in settings where children gather (e.g., preschools, schools, sport and cultural events).
Food prices	Pricing policies (e.g., taxes, subsidies, and incentives) are aligned with health outcomes by helping to make the healthy eating choices the easier, cheaper choices.	Taxes on healthy foods are minimised to encourage healthy food choices where possible (e.g., low or no sales tax, excise, value-added or import duties on fruit and vegetables).
		Taxes on unhealthy foods (e.g., sugar-sweetened beverages, foods high in nutrients of concern) and cigarettes are in place to discourage unhealthy food choices where possible, and these taxes are reinvested to improve population health.
		The intent of existing subsidies on foods, including infrastructure funding support (e.g., research and development, supporting markets or transport systems), is to favour healthy rather than unhealthy foods.
		The government ensures that food-related income support programs are for healthy foods.

Domains	Proposed good practice	Indicators
Food provision	The government ensures that there are healthy policies implemented in government-funded settings to ensure that provision encourages healthy food choices and discourages unhealthy food choices, and the government actively supports private companies to implement similar policies.	The government ensures that there are clear, consistent policies (including nutrition standards) implemented in schools and early childhood education services for food service activities (canteens, food at events, fundraising, promotions, vending machines etc.) to provide/promote healthy food choices.<br><br>The government ensures that there are clear, consistent policies in other public sector settings for food service activities (canteens, food at events, fundraising, promotions, vending machines, public procurement standards etc.) to provide/promote healthy food choices.<br><br>The government ensures that there are good support and training systems to help schools and other public sector organisations and their caterers meet the healthy food service policies and guidelines.<br><br>Government actively encourages and supports private companies to provide and promote healthy foods/meals.
Food retail	The government implements policies and programs to support the availability of healthy foods and limit the availability of unhealthy foods and cigarettes in communities (outlet density and locations) and in-store (product placement).	Zoning laws and policies are robust enough and are being used, where needed, by local governments to place limits on the density or placement of quick serve restaurants or other outlets selling mainly unhealthy foods in communities.<br><br>Zoning laws and policies are robust enough and are being used, where needed, by local governments to encourage the availability of outlets selling fresh fruit and vegetables.<br><br>Government ensures existing support systems are in place to encourage food stores to promote the in-store availability of healthy foods and to limit the in-store availability of unhealthy foods.<br><br>The government ensures existing support systems are in place to encourage food/cigarette service outlets to increase the promotion and availability of healthy foods and to decrease the promotion and availability of unhealthy foods.
Food trade and investment	The government ensures policy coherence and alignment, and that population health impacts are explicitly considered in the development of government policies (e.g., educational, and agricultural sectors).	Government has established evidence-informed recommended food-based dietary intake guidelines to meet WHO recommendation.<br><br>Government has regulation or act for using evidence in the development of food policies.

Supplementary material 4 – Food-EPI Indicators and domains – Infrastructure support

Table S2: Food-EPI Indicators and domains – Infrastructure support

Domains	Proposed good practice	Indicators (Good practice statements)
Leadership	The political leadership ensures that there is strong support for the vision, planning, communication, implementation and evaluation of policies and actions to create healthy food environments, improve population nutrition, and reduce diet-related inequalities.	There is strong, visible, political support (at the Head of State / Cabinet-level) for improving food environments, population nutrition, diet-related NCDs and their related inequalities.<br><br>Clear population intake targets have been established by the government for the nutrients of concern to meet WHO and national recommended dietary intake levels.<br><br>Clear, interpretive, evidence-informed food-based dietary guidelines have been established and implemented.<br><br>There is a comprehensive, transparent, up-to-date implementation plan (including priority policy and program strategies, social marketing for public awareness and threat of legislation for voluntary approaches) linked to national needs and priorities, to improve food environments, reduce the intake of the nutrients of concern to meet WHO and national recommended dietary intake levels, and reduce diet-related NCDs.<br><br>Government priorities have been established to reduce inequalities or protect vulnerable populations in relation to diet, nutrition, obesity and NCDs.
Governance	Governments have structures in place to ensure transparency and accountability and encourage broad community participation and inclusion when formulating and implementing policies and actions to create healthy food environments, improve population nutrition, and reduce diet-related inequalities.	There are robust procedures to restrict commercial influences on the development of policies related to food environments where they have conflicts of interest with improving population nutrition.<br><br>Policies and procedures are implemented for using evidence in the development of food policies.<br><br>Policies and procedures are implemented for ensuring transparency in the development of food policies.<br><br>The government ensures access to comprehensive nutrition information and key documents (e.g. budget documents, annual performance reviews and health indicators) for the public.
Monitoring and Intelligence	The government's monitoring and intelligence systems (surveillance, evaluation, research and reporting) are comprehensive and regular enough to assess the status of food environments, population nutrition and diet-related NCDs and their inequalities, and to measure progress on achieving the goals of nutrition and health plans.	Monitoring systems, implemented by the government, are in place to regularly monitor food environments (especially for food composition for nutrients of concern, food promotion to children, and nutritional quality of food in schools and other public sector settings), against codes/guidelines/standards/targets.<br><br>There is regular monitoring of adult and childhood nutrition status and population intakes against specified intake targets or recommended daily intake levels.<br><br>There is regular monitoring of adult and childhood overweight and obesity prevalence using anthropometric measurements.<br><br>There is regular monitoring of the prevalence of NCD risk factors and occurrence rates (e.g., prevalence, incidence, mortality) for the main diet-related NCDs.

Domains	Proposed good practice	Indicators (Good practice statements)
		There is sufficient evaluation of major programs and policies to assess effectiveness and contribution to achieving the goals of the nutrition and health plans.<br><br>Progress towards reducing health inequalities or health impacts in vulnerable populations and societal and economic determinants of health are regularly monitored.
Funding and resources	Sufficient funding is invested in 'Population Nutrition Promotion'(estimated from the investments in population promotion of healthy eating and healthy food environments for the prevention of obesity and diet-related NCDs, excluding all one-on-one promotion (primary care, antenatal services, maternal and child nursing services etc.), food safety, micronutrient deficiencies (e.g. folate fortification) and undernutrition) to create healthy food environments, improved population nutrition, reductions in obesity, diet-related NCDs and their related inequalities.	The 'Population Nutrition Promotion' budget, as a proportion of total health spending and/or in relation to the diet-related NCD burden is sufficient to reduce diet-related NCDs.<br><br>Government funded research is targeted for improving food environments, reducing obesity, NCDs and their related inequalities.<br><br>There is a statutory health promotion agency in place that includes an objective to improve population nutrition, with a secure funding stream.
Platforms for interaction	There are coordination platforms and opportunities for synergies across government departments, levels of government, and other sectors (NGOs, private sector, and academia) such that policies and actions in food and nutrition are coherent, efficient and effective in improving food environments, population nutrition, diet-related NCDs and their related inequalities.	There are robust coordination mechanisms across departments and levels of government (national and local) to ensure policy coherence, alignment, and integration of food, obesity and diet-related NCD prevention policies across governments.<br><br>There are formal platforms between government and the commercial food sector to implement healthy food policies.<br><br>There are formal platforms for regular interactions between government and civil society on food policies and other strategies to improve population nutrition.<br><br>The government leads a broad, coherent, effective, integrated, and sustainable systems-based approach with local organisations to improve the healthiness of food environments at a national level.
Health in all policies	Processes are in place to ensure policy coherence and alignment, and that population health impacts are explicitly considered in the development of government policies.	There are processes in place to ensure that population nutrition, health outcomes and reducing health inequalities or health impacts in vulnerable populations are considered and prioritised in the development of all government policies relating to food.<br><br>There are processes (e.g. health impact assessments) to assess and consider health impacts during the development of other non-food policies.

Supplementary material 5 - Questions for rating workshop

Questions for rating workshop

This survey aims to evaluate the level of implementation of Food-EPI indicators in [*South Asian Country*].

Please enter your rating (1-6) for each of the good practice statements on the degree of implementation of policies or infrastructure support towards good/best practice in the appropriate box

1 = Not implemented or < 20% implemented, (Policy is not implemented/Does not exist or very low level of implementation)

2 = 'Weak', 20-40% implemented (Policy is implemented but no/low level of enforcement)

3 = 'Moderate', 40-60% implemented (Medium level of Implementation and enforcement)

4 = 'Strong', 60-80% implemented (Highly implemented and enforced)

5 = 'Very strong', 80-100% implemented (Very highly implemented and enforced)

6 = I do not know / I cannot rate

If you cannot rate any good practice statement, please select the 'I do not know/ I cannot rate' option (Option 6)

Specific comments relating to the statement/s can be added in the section beside the statement.

General comments relating to the specific policy or infrastructure support domains can be written at the end of this survey.

We used a Likert scale of 1 to 5 in which '1' represented the policy did not exist (non-existent), '2' indicated the implementation of the policy was weak, '3' the implementation of the policy was moderate, '4' the implementation of the policy was strong, and '5' the implementation of the policy was very strong. When evaluating the implementation of policies aimed at enhancing food environments, a rating of '1' could specifically indicate that the policy is largely unimplemented or has negligible presence. In the context of, for example, promoting healthy food choices in schools, a '1' might suggest a minimal or non-existent effort in implementing such policies. On the other hand, a '2' could signify a preliminary stage of implementation, where there is room for substantial improvement. An illustrative example could be insufficient promotion of nutritional information in food establishments. Progressing to '3' could imply a commendable effort with positive strides, yet notable gaps, such as having moderate accessibility to healthy food options but lacking in comprehensive marketing restrictions for unhealthy products. Moving further to '4' might denote a well-established and effective implementation, for instance, if there is a strong regulation in place for clear nutritional labelling and restrictions on unhealthy food marketing. Finally, a '5' could signify near perfect or optimal execution, such as achieving 80-100% implementation of policies that comprehensively address aspects like school nutrition standards, food promotion restrictions, and public awareness campaigns.

Supplementary material 6 – Policy evidence document- Food environment policy index (Food-EPI) – Bangladesh

Policy domains

1 FOOD COMPOSITION

COMP1:

Government introduced legislations to ban sale, marketing, and manufacturing of industrially produced foods and foodstuffs containing more than 2% of trans-fat in 100 grams total fat of a saturated food product.[17] Beyond that country refers to regulations around food safety and hygiene.[18] Additionally, standards related to some food additives and aids are advertised.[19]

COMP2:

There are no specific food composition targets/standards for out of home meals for the content of the nutrient of concern. Country refers to regulations around food hygiene and safety.[18]

2 FOOD LABELLING

LABEL1:

The standards for labelling of pre-packaged foods was published in year 2008 by the Bangladesh Standards and Testing Institution.[20] This also further strengthened with the legislation provided through the food safety act.[18] Furthermore, Multisectoral Action Plan for Prevention and Control of Non-Communicable diseases also promote nutritional labelling, according to but not limited to international standards, in particular the Codex Alimentarius, for all pre-packaged foods including those for which nutrition or health claims are made.[21]

LABEL2:

Apart from labelling standards, food safety labelling regulations also prevents false, misguided information advertised with the food labelling, which can be charged.[22] Under the Food Safety Act, food courts are defined with their powers and jurisdiction.[18] Any person, including food purchaser, consumer, food receiver or user, may lodge a complaint in writing to the chairman or any person authorized by him in this behalf or an inspector, in respect of any anti-food safety practice under this Act. However, capacity of the regulatory bodies to monitor and implement the provisions of the provisions of the food safety act.

LABEL3:

There are food labelling standards in Bangladesh, however these require only listing of food composition, so labels convey only nutritional information without giving any evaluation of the healthiness of the product. The Multisectoral Action Plan for Prevention and Control of Noncommunicable Diseases recommended "promotion of nutritional labelling, according to but not limited to international standards, in particular the Codex Alimentarius, for all pre-packaged foods including those for which nutrition or health claims are made".[21] However, introduction of user friendly and interpretative food labelling is yet to be introduced and enforced at national scale.

LABEL4:

There is no government guidance for visible systems of menu labelling in restaurants and labelling is generally not practiced at the restaurants.

3 FOOD/ PROMOTION

PROMO1:

Currently there is no specific policies targeted to restrict exposure and power of promotion of unhealthy foods through broadcast media, but Pure Food Ordinance (2005) prevents publishing of false or misleading information in advertisement.[23] Multisectoral action plan for NCD prevention of Bangladesh recommends supporting consumer protection groups in Bangladesh to advocate and discourage marketing of unhealthy foods and non-alcoholic beverages to children and ban advertising, promotion and sponsorship of unhealthy diet.[21]

PROMO2:

There are no effective policies specifically designed to restrict exposure and promotion of unhealthy foods through non-broadcast media. However, the Pure Food Ordinance limit false labels and false advertisements.[23] The Multisectoral NCD action plan recommends implementation of actions to support consumer protection groups in Bangladesh to advocate and discourage marketing of unhealthy foods and non-alcoholic beverages to children and ban of advertising, promotion and sponsorship of unhealthy diet.[21]

PROMO3:

Currently no specific policies or regulations to ensure unhealthy foods are not commercially promoted to children in settings where children gather. Bangladesh Breast Milk Substitute Act (2013) prohibited promotion of commercially prepared foods for children under five years; although enforcement of the act is poor and promotion of unhealthy foods for children are going on unabated. However government of Bangladesh planning to implement 3 year operational plan for health promotion which aims to promote healthy diet, support consumer protection groups in Bangladesh to advocate and discourage marketing of unhealthy foods and non-alcoholic beverages to children and discourage sale of processed foods high in harmful fats, sugars and salt in schools and workplace catering facilities.[21]

PROMO4:

No evidence of availability of related policy.

4 FOOD PRICES

PRICES1:

No evidence of availability of related policy.

PRICES2:

Currently no specific taxes on unhealthy foods and beverages. Government of Bangladesh planning review proposals to implement taxes on SSBs[21].

PRICES3:

Government's open market system (OMS) sold rice and other commodities at almost 30 percent less than the market rate, with each household entitled to 5kg of rice and 3kg of atta (wheat flour). The government sells rice, a staple, directly to the poor whenever there is a shortage of supply and prices go beyond their purchasing capacity at lower price or distribute free of cost.[24] In addition diet charts prepare on the basis of local menus at low costs, yet attaining balanced nutrition to increase availability through local production of low-cost items for balanced nutrition.[25]

PRICES4:

Income Support Program for the Poorest (ISPP) Project also known as the “Jawtno” Programme, provides cash benefits program for the poorest mothers and pregnant women in exchange for their participation in activities to improve their children’s nutrition and cognitive development. ISPP is built upon the success of the pilot Shombhob, which found that beneficiary households experienced an increase in consumption of nutritious foods and children’s health outcomes when cash transfers were linked to the use of growth monitoring and nutritional counselling services.[26] Additionally direct distribution to households for emergency relief by the Ministry of Food and Disaster Management, in order to increase access to food by poor households.[25]

5 FOOD PROVISION

PROV1:

There are no specific policies/laws/recommendations to regulate food service activities across education institutions. However, government planned to provide hot-cooked meal ‘mid-day meal’ to all primary school children in addition to the micronutrient fortified biscuits. This aims to ensure standard nutritious meal provided to school children.

PROV2:

No evidence of availability of related policy.

PROV3:

There are no targeted support training systems for public sector organizations and schools for their caterers for meet healthy food guidelines. However, Nutrition education programmes in National Food Policy 2006 highlights for need support systems including, ensure well planned and efficient training in nutrition for all rural workers, local leaders, school teachers, imams, boy scouts and girl guides, strengthening nutrition education in appropriate academic institutions and in the training of agricultural extension workers, teach all people about the proper preparation and feeding of weaning foods made from locally available foods.[25,27]

PROV4:

No evidence of availability of related policy.

6 FOOD RETAIL

RETAIL1:

No evidence of availability of related policy.

RETAIL2:

No evidence of availability of related policy.

RETAIL3:

Country did not identify any relevant actions/policies in this indicator. But the only available are to regulate the food hygiene and safety and standards. (The Pure Food Ordinance, 1959 as amended by the Bangladesh Pure Food (Amendment) and the Bangladesh Standards and Testing Institution Ordinance, 1985 as amended in 2003 Act).

RETAIL4:

Apart from the standards for food safety,[23,19] there are no specific policies to increase the promotion and availability of healthy foods and to decrease the promotion and availability of unhealthy foods.

7 FOOD TRADE AND INVESTMENT

TRADE1

No evidence of availability of related policy.

TRADE2

No evidence of availability of related policy.

Infrastructure support domains

8 LEADERSHIP

LEAD1:

There is evidence that policymakers are committed to ensure food security and improve the nutrition and health of the population as evident in formulation the nutrition, food and health policies and related plan of actions. Policymakers are also committed to ensure safe foods for the population groups as evident by formulation of the Food Safety (Labelling) Regulations 2017. The food act and the National Nutrition Policy also provides directives from the leadership in implementing the regulations and laws.[18,27]

LEAD2:

Dietary guideline for Bangladesh suggested some nutrient goals for ensuring health and nutrition of the population of Bangladesh.[28]

LEAD3:

Bangladesh developed a dietary guideline in 2014. However, this need to be updated and further improvement is required to make it more user friendly.[28]

LEAD4:

No evidence of availability of related policy.

LEAD5:

Government has developed strategies and guidelines to improve food security, nutrition, health and protect the food security of the vulnerable groups. But strategies to address the inequality in addressing NCDs are lacking.

9 GOVERNANCE

GOVER1:

No evidence of availability of related policy.

GOVER2:

Policies are generally formulated through a process of consultations with key stakeholders.[29]

GOVER3:

Clear strategies to ensure transparency need to be developed further.[29,30]

GOVER4:

Budgetary allocations of government are available at the website of concerned ministries. Detail description on the nutrition information and the key documents are also available through the publications of the relevant ministries.

10 MONITORING AND INTELLIGENCE

MONIT1:

No evidence of availability of related policy.

MONIT2:

Food security and nutrition related information is collected through various surveys (Demographic and Health Survey, Food Security and Nutrition Survey, STEP Survey, Household Income and Expenditure Survey). However, none of them cover all age groups and are conducted on a regular interval. Bangladesh National Nutrition Council recently developed a guideline and recommended to conduct such studies on a regular interval.[31]

MONIT3:

Food security and nutrition related information is collected through various surveys (Demographic and Health Survey, Food Security and Nutrition Survey, STEP Survey, Household Income and Expenditure Survey). However, none of them cover all age groups and are conducted on a regular interval. Bangladesh National Nutrition Council recently developed a guideline and recommended to conduct such studies on a regular interval.[31]

MONIT4:

Food security and nutrition related information is collected through various surveys (Demographic and Health Survey, Food Security and Nutrition Survey, STEP Survey, Household Income and Expenditure Survey). However, none of them cover all age groups and are conducted on Regular interval. Bangladesh National Nutrition Council recently developed a guideline and recommended to conduct such studies on a regular interval.[31]

MONIT5:

No evidence of availability of related policy.

MONIT6:

No evidence of availability of related policy.

11 FUNDING AND RESOURCES

FUND1:

Bangladesh National Nutrition Council monitor the budgetary allocation of different ministries and some information is available through its website.

FUND2:

No evidence of availability of related policy.

FUND3:

Several organizations (Bangladesh National Nutrition Council, Institute of Public Health Nutrition) from public sector are assigned to promote health and nutrition of the public health.

12 PLATFORMS FOR INTERACTION

PLATF1:

Bangladesh National Nutrition Council is assigned to do the job, but its capacity needs to be strengthened.

PLATF2:

No evidence of availability of related policy.

PLATF3:

With support from Global Alliance for Improved Nutation (GAIN) The Civil Society Alliance for Scaling up Nutrition (SUN) play some role. Enough information in not available.

PLATF4:

No evidence of availability of related policy.

13 HEALTH IN ALL POLICIES

HIAP1:

No evidence of availability of related policy.

HIAP2:

No evidence of availability of related policy.

Supplementary material 7 - Policy evidence document- Food environment policy index (Food-EPI) – India

Policy domains

1 FOOD COMPOSITION

COMP1:

In 2015, Food Safety and Standards Authority of India (FSSAI) passed an order, F.No.5/13/CERC/Salt/FSSAI/2015, 'Regulating Salt, Sugar & Fat in Indian Food' and constituted the Expert Group on Salt, Sugar & Fat in Food Products in India. The expert group published a comprehensive report containing all requisite information on these foods that served as a guideline document for stakeholders including industry, FSSAI and consumers in rationalizing the consumption of fat, sugar and salt through processed food products.[32] Reinforcement of the nutrient specific guideline was done which suggested that out of total energy intake per day the trans fatty acids must be <1 %, sugar intake in daily diet must be < 10% and added salt must be 5-6g/day as recommended by WHO and ICMR.[33]

COMP2:

There are no specific food composition targets/standards for out of home meals in the restaurants by the government. However, FSSAI has created the Eat Right Campus Movement to promote a healthy eating environment in places where most people spend most of their time, such as workplaces, colleges, and universities. This includes everything from in-house canteens to catering and meal delivery services, as well as restaurants, cafes, and street food vendors[34]. The 'Orange Book for Eat Right Campus' was created as a resource book that summarizes these best practices and demonstrates how to put them into reality in a straightforward and illustrative manner.[35]

2 FOOD LABELLING

LABEL1:

According to Food Safety and Standards (Packaging and Labelling) Regulation, 2011, F. No. 2-15015/30/2010, it is necessary to label nutritional information per 100g or 100mL per serving of the product that must be mentioned on the label containing the energy value in kcal, the amounts of protein, carbohydrate (with specification of sugar quantity) and fat in gram (g) or millilitre (mL). If a claim is made regarding the amount or type of fatty acid or the amount of cholesterol, it is mandatory to specify the type of fatty acid and its amount with units.[36] In 2020 the regulations were updated by FSSAI by inserting the provision stating that cholesterol content to be given only for products containing fats of animal origin and where total fat content is more than 0.5%.

LABEL2:

According to Food Safety and Standards (Packaging and Labelling) Regulation, 2011, F. No. 2-15015/30/2010, if a claim is made regarding the amount or type of fatty acid or the amount of cholesterol, it is mandatory to specify the type of fatty acid and its amount with units. A health claim of 'trans-fat free' in a processed food item can be made only in cases where the trans-fat is less than 0.2g per serving of food and the claim 'saturated fat free' can be made in cases where the saturated fat does not exceed 0.1g per 100g or 100mL of food.[36]

LABEL3

As mentioned by FSSAI in Draft Food Safety and Standards (Labelling and Display) Regulations, 2018, F. No 1-94/FSSAI/SP (Labelling)/2014 (Pt-2), the food items considered as "High Fat, Sugar and Salt" [HFSS] must be depicted with red blocks within the specified thresholds. Also, in those premises where HFSS food with red labels is being served, it is mandatory to display this message on boards to encourage people to eat healthy food.[36]

LABEL4:

The standards for 'Display of information in Food Service Establishments' are specified in Sub-Regulation 2.4.6 of the Food Safety and Standards (Packaging and Labelling) First Amendment Regulations, 2020, dated August 21, 2020. Menu Cards/ Menu Boards/Include Boards/Booklets must display the calorific value of the food product/dish, allergen information, and the vegetarian/non-vegetarian food logo. Furthermore, where the menu/dish/product is displayed for information or ordering, the handouts/booklets/website/digital application/advertising material (both digital and printed) must include complete nutritional information and other declarations as specified in the laws.[37] The 'Eat Right Movement' initiated by FSSAI, in July 2018 has one major ongoing initiative named, "The Safe and Nutritious Food Initiative", which represents the commitment made by the food service industry to introduce menu labelling and to promote significantly healthier food options.[34]

3 FOOD PROMOTION

PROMO1:

The regulation on the Food Safety and Standards (Advertising and Claims), 2018 describes the general principles for claims and advertisements; criteria for nutrition claims (including nutrient content or nutrient comparative claims), non-addition claims (including non-addition of sugars and sodium salts), health claims (reduction of disease risk), claims related to dietary guidelines or healthy diets, and conditional claims; claims that are specifically prohibited; and procedures for approval of claims and redressal of non-compliances under these regulations.[38]

PROMO2:

The regulation on the Food Safety and Standards (Advertising and Claims), 2018 also covers the non-broadcast media.[38]

PROMO3:

"Guidelines for Promoting Wholesome and Nutritious Food and Restricting/ Limiting the Availability of Foods High in Fat, Sugar and Salt (HFSS Foods) among School Children" proposed that HFSS foods should not be advertised in front of children in any form.[39] According to "The Eat Right Movement" one of the main goals was to regulate the positioning of outlets promoting unhealthy food items at places where children gather.[34]

4 FOOD PRICES

PRICES1:

According to Ministry of Finance, Department of Revenue, Central Board of Indirect Taxes and Customs, Goods and Services Tax fresh and/or refrigerated fruits and vegetables are now tax-free under the GST, which is in accordance with previous VAT rules in various states. As a result, fresh, unprocessed items purchased directly from a farmer, dealer, or merchant are exempted from GST.[40]

PRICES2:

In 2016, as part of the budget, the government of Kerala, Ministry of Finance and Coir levied a tax rate of 14.5% as "Fat Tax" on burgers, pizzas, tacos, donuts, sandwiches, burger-patties, pasta, bread fillings and other cooked food items sold by branded restaurants. This action was taken to control the increasing prevalence of obesity amongst the population.[41] In July 2017 Government of India introduced sin tax on sugary carbonated drinks to tax of 40%. These drinks are put under the highest tax bracket of the Goods and Services Tax (GST) regime in India, at 28%.[42]

PRICES3:

The National Food Security Act (NFSA), 2013 was passed by the parliament of India on10th September with the objective to provide good quality food at affordable prices. The Act provides coverage of up to 75% of the rural population and up to 50% of the urban population to receive subsidized food grains under Targeted Public Distribution System (TPDS), thus covering about two-thirds of the population. Eligible people received 5 kg of food grains per person per month at subsidized prices of Rs. 3/2/1 per kg for rice/wheat/coarse grains. The existing Antyodaya Anna Yojana (AAY) households, which constitute the poorest of the poor, will continue to receive 35kg of food grains per household per month.[43] However, this policy is for white rice which is considered unhealthy.

PRICES4:

The food related direct income support programs not available. However low-income families are supported through the World Food Programme was set up in India in 1963 to ensure that good quality food reaches those who need it the most. The programme focuses on transitioning from food distribution to technical assistance and improving the efficiency, accountability, and transparency of India’s own subsidized food distribution system. It has its own subsidized food distribution system and brings supplies of wheat, rice, sugar and kerosene oil to around 800 million poor people.[44]

5 FOOD PROVISION

PROV1:

Guidelines for Promoting Wholesome and Nutritious Food and Restricting/ Limiting the Availability of Foods High in Fat, Sugar, and Salt (HFSS) among School Children, F.No.1/School Guidelines/IEC/FSSAI-2015-16 was proposed by FSSAI in 2015. According to these guidelines, promotion of HFSS foods must be prohibited in schools and the availability of most common HFSS foods in schools within 50m must be restricted. The school canteen policy should consider and introduce the concept of colour coding to categorize the foods i.e., foods that should be eaten most in green category, foods that should be eaten sparingly in yellow category and most common HFSS foods in red category. This colour coding system should be adopted by all kinds of school. [33] In 2016, Down to Earth article reported that CBSE has taken initiative and generated a circular in reference to these guidelines and asked 17,000 CBSE affiliated schools to implement these guidelines in school canteens.[45]

PROV2:

No evidence of availability of related policy. However, an innovative initiative was taken in South India by a private organization named as Snaxsmart. It is based on the concept of providing healthy snacks through the vending machine to people working in corporate offices.[46]

PROV3:

FSSAI provides training and certification under the Food, Safety, Training and Certification also called FoSTaC. FSSAI recommends that all licensed food businesses must have at least one trained and certified 'Food Safety Supervisor' under FoSTaC for every 25 food handlers in each premise. They provide training courses to people working in food related areas. This programme is designed according to the laws and guidelines.[47]

'Serve safe' is another initiative by FSSAI to enable food businesses, food handlers and consumers to ensure that the food which is served in hotels, restaurants and canteens is of high quality, safe and hygienic. Three key components are-training of food safety supervisors based on a standard curriculum, installation of 'Food Safety Display Boards' as a feedback mechanism for improved interface with consumers and 'hygiene + rating' to raise the bar continuously. Going beyond hygiene, 'hygiene + rating' is to sensitize food businesses towards human health and sustainability.[48]

PROV4:

A nation-wide campaign named 'Safe and Nutritious Food @ workplace' was initiated by FSSAI in 2017 to promote healthy eating, audits regarding licensing and registration with FSSAI of vendors and checks upon the Food Safety Management System for in-house and external food catering services.[49]

6 FOOD RETAIL:

RETAIL1:

Food Safety and Standards (Safe food and balanced diets for children in school) Regulations restrict the availability of foods high in saturated fat, trans fat, added sugar or sodium in or within a fifty-metre of schools. For the first time, there will be a law to regulate availability of junk foods in and around schools.[39]

RETAIL2:

No evidence of availability of related policy.

RETAIL3:

No evidence of availability of related policy.

RETAIL4

FSSAI initiated a campaign in July 2018, wherein the key stakeholders like food processing industries pledged to reformulate packaged foods and restaurants promised to provide healthier food options and introduce menu-labelling, food retailers including e-commerce players agreed to promote healthier food options and responsible retail practices to reduce the percentage of salt, sugar, and fat content in food. The edible oil industry and bakeries committed to phase out trans-fats by 2022 fat. The food services sector as part of the 'Eat Right Movement', food businesses and food service sector were pushed to promote healthier food options in different ways in addition to an initiative to turn India into a 'Trans-fats-free India' by 2022.[34]

7 FOOD TRADE AND INVESTMENT

TRADE1:

No evidence of availability of related policy.

TRADE2:

No evidence of availability of related policy.

Infrastructure support domains

8 LEADERSHIP

LEAD1:

Concerned about the rise in lifestyle diseases, the Food Safety and Standards Authority of India (FSSAI) created the Eat Right India initiative to empower Indians by enhancing their health and well-being. It is based on two basic pillars: Eat Healthy and Eat Right; it is a collaborative effort including key stakeholders to make both demand and supply-side adjustments.[34]

LEAD2:

There are Food based Dietary Guidelines for Indians by National Institute of Nutrition, ICMR, Hyderabad. These dietary guidelines place a strong emphasis on health promotion and disease prevention, sickness, affecting people of all ages, with a specific focus on the most vulnerable. Infants, children, and adolescents, as well as pregnant and lactating women, make up a large portion of the population. It explains the importance of right nutritional behaviour and dietary choices which are needed to achieve dietary goals among diverse Indian population.[50]

LEAD3:

There are Food based Dietary Guidelines for Indians by National Institute of Nutrition, ICMR, Hyderabad. These dietary guidelines place a strong emphasis on health promotion and disease prevention, sickness, affecting people of all ages, with a specific focus on the most vulnerable. Infants, children, and adolescents, as well as pregnant and lactating women, make up a large portion of the population. It explains the importance of right nutritional behaviour and dietary choices which are needed to achieve dietary goals among diverse Indian population.[50]

LEAD4:

No evidence of availability of related policy.

LEAD5:

No evidence of availability of related policy.

9 GOVERNANCE

GOVER1:

No evidence of availability of related policy.

GOVER2:

India adopted National Nutrition Policy, by Department of Women and Child Health, Government of India in 1993. It clearly explains the need for nutrition policy, nutrition status of India, existing policy instruments to combat malnutrition, administration and monitoring, along with intervention programmes to combat malnutrition.[51]

GOVER3:

The Food Safety and Standards Authority of India (FSSAI) is a statutory organisation under the Government of India's Ministry of Health and Family Welfare. FSSAI is in charge of preserving and promoting public health by regulating and supervising food safety and is responsible for implementation of all the food policy and regulations to be followed according to global benchmarked food standard and practices in the country.[52]

GOVER4:

No evidence of availability of related policy.

10 MONITORING AND INTELLIGENCE

MONIT1:

There is no clear evidence exists for regular, periodic monitoring systems implemented by the government for food environment. But The National Nutrition Institute, ICMR, Government of India, conducts research on community nutrition and dietetics, as well as conducting ongoing and special surveys for the National Nutrition Monitoring Bureau (NNMB) which focuses on planning and executing diet and nutrition surveys, reviewing ongoing nutrition programmes, and conducting studies on socio-cultural elements of nutrition, the field division works closely with state governments, the federal government, and international agencies.[53]

MONIT2:

The National Nutrition Institute, ICMR, Government of India, conducts research on community nutrition and dietetics, as well as conducting ongoing and special surveys for the National Nutrition Monitoring Bureau (NNMB) which focuses on planning and executing diet and nutrition surveys, reviewing ongoing nutrition programmes, and conducting studies on socio-cultural elements of nutrition, the field division works closely with state governments, the federal government, and international agencies.[53]

MONIT3:

National Family Health Survey provides estimates on key indicators related to population, family planning, child and maternal health, nutrition, adult health, and domestic violence, Through the survey obesity is also estimated as an indicator between men and women aged 15 to 49 across all states to find out the prevalence of obesity in our country.[54]

MONIT4:

In 2017, Ministry of Health and Family Welfare has launched an action plan for the prevention and control of NCD’s, namely National Programme for Prevention and Control of Cancer, Diabetes, Cardiovascular diseases, and Stroke (NPCDCS) in India with an operations guideline for surveillance, community-based interventions and promotion on healthy diet and lifestyle modifications to prevent and control t diabetes, cardiovascular diseases, obesity, stroke, common cancers and other NCDs. The prevention aspect of the programme includes strategies on obesity and diabetes, sodium intake, alcohol intake, high blood pressure and lack of physical activity.[55]

MONIT5:

No evidence of availability of related policy.

MONIT6:

No evidence of availability of related policy.

11 FUNDING AND RESOURCES

FUND1:

There is no documentary evidence to comment on adequacy or inadequacy. But to converge nutrition-related initiatives and strengthen outcomes, the Government of India (GoI) restructured the Integrated Child Development Services (ICDS), POSHAN (Prime Minister's Overarching Scheme for Holistic Nourishment) Abhiyaan, the Scheme for Adolescent Girls, and the National Creche Scheme into Saksham Anganwadi and POSHAN 2.0 in

Financial Year (FY) 2021-22. For FY 2022-23 Budget Estimates (BEs), allocations for Saksham Anganwadiand POSHAN 2.0 stood at 20,263 crores. This is 1 per cent higher than FY 2021-22 Revised Estimates (REs), which stood at 20,000 crore.[56]

FUND2:

The Department of Health Research, under Ministry of Health and Family Welfare's mission is to bring modern health technologies to people through research and innovations in diagnosis, treatment methods, and vaccines for prevention; to translate these innovations into products and processes; and to introduce them into the public health system in collaboration with concerned organisations.[57]

FUND3:

In India, Ministry of Health and Family Welfare under Government of India is responsible for the overall health development and promotion across the country. Directorate General of Health Services (DGHS) is attached office of the Department of Health & Family Welfare and has subordinate offices spread all over the country. The DGHS provides technical assistance on all medical and public health issues, as well as participating in the delivery of various health services.[58]

12 PLATFORMS FOR INTERACTIONS

PLATF1:

In 2017, the Ministry of Health and Family Welfare, Government of India came up with a National Multisectoral Action Plan to Prevent and Control Common NCDs (2017-2022). This plan was made in collaboration with the different stakeholders from all over the country such as Union Ministers, State Governments, Private Sector, Civil Society, Professional Bodies, Academia and International Partners.[55]

PLATF2:

The Food Safety and Standards Authority of India (FSSAI) is intended to ensure the availability of safe and wholesome food for the people of India, according to the preamble of the Food Safety and Standards Act, 2006. As a result, through the 'Eat Right India' programme, the FSSAI has started on a large-scale effort to overhaul the country's food system to assure safe, healthy, and sustainable food for all Indians. The phrase 'Sahi Bhojan. Behtar Jeevan' thus serves as the movement's cornerstone. Eat Right India uses a balanced strategy of regulation, capacity building, collaboration, and empowerment to ensure that our food is healthy for both people and the environment. It also relies on the combined efforts of all stakeholders, including the government, food companies, civil society organizations, experts and professionals, development agencies, and the general public.[34]

PLATF3:

No evidence of availability of related policy.

PLATF4:

The Food Safety and Standards Authority of India (FSSAI) is intended to ensure the availability of safe and wholesome food for the people of India, according to the preamble of the Food Safety and Standards Act, 2006. As a result, through the 'Eat Right India' programme, the FSSAI has started on a large-scale effort to overhaul the country's food system to assure safe, healthy, and sustainable food for all Indians. The phrase 'Sahi Bhojan. Behtar Jeevan' thus serves as the movement's cornerstone. Eat Right India uses a balanced strategy of regulation, capacity building, collaboration, and empowerment to ensure that our food is healthy for both people and the environment. It also relies on the combined efforts of all stakeholders, including the government, food companies, civil society organizations, experts and professionals, development agencies, and the general public.[34]

13 HEALTH AND HEALTHY BEHAVIOR IN ALL POLICIES

HIAP1:

No evidence of availability of related policy.

HIAP2:

No evidence of availability of related policy.

Supplementary material 8 – Policy evidence document – Food environment policy index (Food-EPI) – Pakistan

Policy domains

1 FOOD COMPOSITION

COMP1:

No evidence of availability of related policy.

COMP2:

No evidence of availability of related policy.

2 FOOD LABELLING

LABEL1:

On February 19, 2019, Pakistan's Ministry of Commerce (MOC) issued a statutory regulatory order (i.e., SRO) concerning labelling of food products. The SRO requires the following label information: 1) a minimum 66% shelf life at the time of clearance of goods, 2) labelling of nutritional values and usage instructions in Urdu and English language and 3) the accreditation authority of the country that validated the Halal Certificate or Halal Certificate issuing authority needs to be a member of either IHAF or SMIIC. These rules are in effect on February 19, 2019.[59]

LABEL2

The Punjab food Authority has been working as a functional entity in Punjab Province across all 36 districts since July 2012. Specialized wings "Resource & Licensing", "Technical" and "Operations" which are functional and working to enforce the food hygiene and quality standards as described in the Punjab Food Authority Act 2011 and the Pure Food Rules 2011 is carried out through qualified teams of Food Safety Officers (FSOs') and Assistant Food Safety Officers (AFSOs'). Food Safety teams ensure the food safety. Punjab Food Authority have state of the art labs working through out the clock which carry out the food testing for food hygiene and food adulteration under the supervision of the qualified staff. [60]

LABEL3:

No evidence of availability of related policy.

LABEL4:

No evidence of availability of related policy.

3 FOOD PROMOTION

PROMO1:

No evidence of availability of related policy.

PROMO2:

No evidence of availability of related policy.

PROMO3:

No evidence of availability of related policy.

4 FOOD PRICES

PRICES1:

Government of Pakistan's proposed to exempt vitamin premixes and food grade minerals and micronutrients from customs duties and sales tax in its 2016-2017 Finance Bill.[61] In 2013, the Federal Board of Revenue has issued the list of products which are exempted General Sales Tax (GST). The list includes vegetables; meat; milk and yogurt; butter; eggs; poultry; red chili; lentils, bread, and nan; fruits; ginger; turmeric; ice; medicines; sugarcane and salt.

PRICES2:

The government of Pakistan imposed sin tax and sugar-sweetened beverages as a much-needed step towards protecting society's right to a healthy life explained by the federal health minister at a conference in Islamabad, in December 2018.[62]

PRICES3:

No evidence of availability of related policy.

PRICES4:

Prime Minister Imran Khan recently announced a Rs 120 billion subsidy under the umbrella of the Ehsas programme. The details that have been shared so far suggest that the subsidy would be given on three food items: sugar, pulses, and flour. The subsidy would be provided to around 20 million families who would be identified through their poverty score card based on the poverty survey conducted back in 2010-2011. The distribution would happen through around 700,000 karyana merchants across the country.[63]

5 FOOD/ PROVISION

PROV1:

The Provincial Food Authorities has imposed a ban on certain food items including carbonated drinks and snacks/chips at canteens across schools and colleges of the Pakistan.[64] Teams of the Food Safety and Halal Food Authority of Balochistan, Khyber Pakhtunkhwa, Punjab, and Sindh visit various schools in the provincial cities to ensure the ban on the sale of prohibited items.[65] The provincial food regulatory body also carried out a special drive to ensure the implementation of the ban on the sale of carbonated drinks in the educational institutions of Punjab in 2018. As many as 846 schools were checked among 18 canteens of the different schools sealed in a daylong operation in the various cities of Punjab.[66]

PROV2:

No evidence of availability of related policy.

PROV3:

Punjab Food Authority (PFA) has formulated complete guidelines for healthy foods and drinks to be sold at the canteens of schools and colleges across the province, dividing food into three categories i.e. Green, Yellow and Red. The green category food will always be on the menu and will be available every day as main choices. It will be presented in attractive and interesting way besides being promoted as tasty and good value choice.[67]

PROV4:

No evidence of availability of related policy.

6 FOOD RETAIL

RETAIL1:

No evidence of availability of related policy.

RETAIL2:

No evidence of availability of related policy.

RETAIL3:

No evidence of availability of related policy.

RETAIL4:

No evidence of availability of related policy.

7 TRADE

TRADE1:

No evidence of availability of related policy.

TRADE2:

No evidence of availability of related policy.

Infrastructure support domains

8 LEADERSHIP

LEAD 1:

No evidence of availability of related policy.

LEAD 2:

No evidence of availability of related policy.

LEAD 3:

No evidence of availability of related policy.

LEAD 4:

No evidence of availability of related policy.

LEAD 5:

Pakistan - No evidence of availability of related policy.

9 GOVERNANCE

GOVER1:

No evidence of availability of related policy.

GOVER2:

The MNFSR plays an overarching role, including monitoring, reporting, and addressing high-level policy issues. The MNFSR will have oversight of policy implementation through an Implementation Committee. The Committee will be chaired by the Federal Secretary of the MNFSR and include the secretaries of relevant ministries, commissions, and programmes at the federal and provincial levels and representatives from academia, the private sector, and civil society organizations. The Committee will review the progress of overall actions at the federal level, including the formulation and enforcement of legislation and regulations, and report regularly to the MNFSR and the prime minister. The MNFSR will also create councils or commissions to monitor and report on specific activities and programmes. One of these will be the National Food Security Council, which will address food and nutritional security issues and comprises of concerned government agencies at the federal and provincial levels, as well as NGOs/CSOs and the private sector.

GOVER3:

No evidence of availability of related policy.

GOVER4:

No evidence of availability of related policy.

10 NONITORING AND INTELLIGENCE

MONIT1:

No evidence of availability of related policy.

MONIT2:

The DHS survey was conducted under the aegis of the Ministry of National Health Services, Regulations and Coordination, and implemented by the National Institute of Population Studies (NIPS). The National Health Survey of Pakistan (NHSP) was conceived to provide accurate health statistics to the Government of Pakistan for proper planning and policy development in the health sector.

MONIT3:

The DHS survey was conducted under the aegis of the Ministry of National Health Services, Regulations and Coordination, and implemented by the National Institute of Population Studies (NIPS). The National Health Survey of Pakistan (NHSP) was conceived to provide accurate health statistics to the Government of Pakistan for proper planning and policy development in the health sector.

MONIT4:

No evidence of availability of related policy.

MONIT5:

No evidence of availability of related policy.

MONIT6:

Achieving food security and nutrition for its population is a high priority for the Government of Pakistan. Several important policy initiatives have been taken in this direction, which include the concept development of the National Zero Hunger Program, the food security assessment survey, the recent commitment of the Government for Sustainable Development Goals, particularly to the SDG-1 and 2 about poverty and Zero Hunger Challenges. To document all these initiatives and future strategies in light of the SDGs, MNSFSR prepared a comprehensive National Food Security Policy.

11 FUNDING AND RESOURCES

FUND1:

No evidence of availability of related policy.

FUND2:

No evidence of availability of related policy

FUND3:

No evidence of availability of related policy

12 PLATFORMS FOR INTERACTION

PLATF1:

The institutional setup for agriculture and food security has undergone significant changes after the devolution that took place following the adoption of 18th Amendment, with the provinces taking over responsibility for agriculture and rural development. The creation of the MNFSR in 2011, which replaced the devolved ministry of Food, Agriculture and Livestock, clearly indicated that there was a need for a new national policy direction, particularly to address key challenges such as technology gaps, food insecurity, and poor nutritional levels The MNFSR will have oversight of policy implementation through an Implementation Committee. The Committee will be chaired by the Federal Secretary of the MNFSR and include the secretaries of relevant ministries, commissions, and programmes at the federal and provincial levels and representatives from academia, the private sector, and civil society organizations. Developing close partnership with the Provincial Governments, entrepreneurs, research scientists, investors, exporters, importers, academia, progressive farmers and civil society for achieving Food Security and reducing health Inequalities.[68]

PLATF2:

No evidence of availability of related policy

PLATF3:

No evidence of availability of related policy.

PLATF4:

No evidence of availability of related policy.

13 HEALTH IN ALL POLICIES

HIAP1:

Achieving food security and nutrition for its population is a high priority for the Government of Pakistan. A number of important policy initiatives have been taken in this direction, which include the concept development of the National Zero Hunger Program, the food security assessment survey, the recent commitment of the Government for Sustainable Development Goals, particularly to the SDG-1 and 2 about poverty and Zero Hunger Challenges. To document all these initiatives and future strategies in light of the SDGs, MNSFSR prepared a comprehensive National Food Security Policy.[68]

HIAP2:

No evidence of availability of related policy

Supplementary material 9 - Policy evidence document- Food environment policy index (Food-EPI) – Sri Lanka

Policy Domain

1 FOOD COMPOSITION

COMP1:

There are no food composition target regulations specified by the Ministry of Health (MoH) of Sri Lanka yet. However, regulations for salt, sugar and fats prepared by the MoH and forwarded to the Cabinet of Ministers. [69] The target of this intervention is to achieve a 30% reduction in mean population intake of salt /sodium by 2025. The National multisectoral action plan for prevention and control of NCDs 2016-2020, planned to conduct advocacy meeting with food producers, processors, retailers to lobby to change the composition of food lobby food industry to manufacture healthy food options motivate food industry to reformulate processed food establish a mechanism to ensure voluntary reduction of salt, sugar and fat establish a mechanism to ensure mandatory reduction of salt, sugar and fat.[70]

COMP2:

There are no food composition targets established for out-of- home meals in food service outlets by the government. National multisectoral action plan for prevention and control of NCDs 2016-2020 has identified development of a policy to limit use of trans fat and use of PHVO (partially hydrogenated vegetable oil) in all processed food and restaurants as a key activity.[70]

2 FOOD LABELLING

LABEL1:

As per the Food (Labelling and Advertising) Regulations 2005 producers and retailers are required by law to provide a complete list of ingredients used in the food by their common names in descending order of their proportions on pre-packaged food products (with exceptions of eggs), even in the absence of a nutrition or health claim.[71] The rules define which nutrients must be listed and on what basis (e.g., per 100g or per serving).

LABEL2:

Food act in Sri Lanka provides regulatory framework for reviewing the claims on foods. No food label shall contain words indicating that it is recommended by a medical practitioner or Association or professional unless prior approval is granted by the Chief Food Authority (Director General of Health Services) Regulations explicitly describes requirements for nutrient content and nutrient function claims. Nutrient content claims may be used on foods only if they are in compliance with conditions specified.[71]

LABEL3:

The container/package of Carbonated beverages, Ready to serve beverages other than milk-based products, Fruit Nectar, Fruit Juice have a colour coded label based on sugar level.[72] The container or the package which contains such food shall have a label which shall adhere to the following (a) a numerical description of the sugar content, (b) a description of the relative sugar level as low sugar, medium sugar or high sugar, (c) a colour code displayed as red, amber or green. The description of the sugar level shall be indicated as high sugar (red colour - >11g per 100ml of drink), medium sugar (amber colour – 2-11g per 100ml of drink) or low sugar (green colour - <2g per 100ml of drink) in all three languages (Sinhalese, Tamil, English).It is mandatory to have traffic light front of pack labelling of solid and semi-solid food packages. The container or the package which contains such food shall have a label which shall adhere to the following;[73] (a) a numerical description of the sugar, salt, fat content per 100g, (b) a description of the relative sugar level as low sugar, medium sugar or high sugar, (c) a colour code logo displayed as red, amber or green.

LABEL4:

No evidence of availability of related policy.

3 FOOD PROMOTION

PROMO1:

Currently, there are no specific government regulations in place to restrict unhealthy food promotion to children through TV, radio, internet, social media, packaging, product placement, magazines, outdoor advertising, sponsorship and point-of-purchase. However, Nutrient profile model for Sri Lanka was published by the Ministry of Health with the prime purpose of regulating the marketing of foods and non-alcoholic beverages to children to reduce the exposure and the power of marketing of food high in fat, salt and sugar.[74] It provides policymakers a platform to regulate/control the marketing of foods and beverages to children and empower consumers to make informed food choices. Regulatory mechanisms will include implementation of restrictions in food advertising to children through media and other means and prohibition of marketing of food and beverages that are identified as "not acceptable" by this model.

The following activities are proposed in the "National multisectoral action plan for prevention and control of NCDs" that are planned to be implemented during the period 2016-2020.[75] Create a mechanism for monitoring of food and beverages advertisements and complains develop national policies on marketing of food and non-alcoholic beverages to children set up a committee to review food advertisement before airing.

PROMO2:

No evidence of availability of related policy

PROMO3:

School canteen policy and school canteen guidelines, targeted to promote healthy eating habits among school children and prevent exposure to unhealthy food through the establishment of a healthy canteen in the school[76]. Foods that are considered as "healthy" and "unhealthy" are listed out in the circular and there are instructions to promote the availability of healthy options and ban or limit the availability of unhealthy options in the school canteen. It is suggested that every school should have a healthy school canteen that is supervised by the school health promotion club. It is expected that the Public Health Inspector of the area should visit the school canteen once per term to observe whether the school canteen adhere to the guidelines. Use of nutrient profile model provides a method of differentiating between foods and beverages that are more likely to be a part of a healthy diet from those that are less likely, by providing thresholds for nutrients of interest regarding overweight / obesity, i.e. fat, sugar and sodium, and energy. It merely provides an objective way of classifying food products as acceptable or not acceptable especially for children.

4 FOOD PRICES

PRICES1:

No evidence of availability of related policy.

PRICES2:

The Sugar Sweetened Beverages (SSB) tax introduced in year 2019. It taxed all water-based flavoured drinks carbonated and non-carbonated, sport, energy, and electrolyte drinks, ready-to-drink form coffee, coffee substitutes, tea, herbal infusions in or calculated as ready to drink form, cereal, grain and tree nut-based beverages produced from the extracts of cereals, beans, pulses and tree nuts beverages.[77] Products where non-sugar sweeteners have been added would also be exempt from the SSB tax. National Multisectoral actin plan for the

prevention and control of NCDs 2016-2020 includes establishment of policies on taxes to discourage consumption of unhealthy food high in fat, sugar and salt as a proposed activity.[75]

PRICES3:

No evidence of availability of related policy.

PRICES4:

Nutrition bag ("Poshana malla" nutrition stamp) for pregnant women who are beneficiaries of government financial assistance programme "Samurdhi" and pregnant women with low BMI in the low income group even if they are not eligible for "Samurdhi", program eligible to receive a Nutrition Relief Card/stamp worth of Rs.500.00 for 20 months from pregnancy to 1 year after childbirth (8 during pregnancy and 12 during lactation).[78] A Nutrition Package includes the white / red rice, Samba rice / boiled rice – 5kg, grams / kawpee / green gram / dhal / – 500g, egg / dried sprats / dried fish (08 eggs or dried fish equal to it). The eligible pregnant women receive a Nutrition Relief Card/stamp worth of Rs.500.00 for 20 months from pregnancy to 1 year after childbirth. (8 during pregnancy and 12 during lactation). A Nutrition Package includes the following items for each stamp per month can be purchased from Cooperative Shop / franchised Sales Outlet. Additionally, every pregnant mother registered with Medical Officer of Health Office are provided this nutrition allowance in the form of a nutrition pack worth Rs.20,000.[79] Nutritious food worth Rs. 2,000 per month are provided under this programme to pregnant mothers during the last 6 months of pregnancy and for the first 4 months after the delivery of the child. They are given vouchers that can be redeemed at the outlets of Cooperative Wholesale Establishment or SATHOSA. Only the food printed on the overleaf of the voucher can be purchased and it should include at least 5 varieties representing different food groups.

5 FOOD PROVISION

PROV1:

The school canteen policy is to promote healthy eating habits among school children and prevent exposure to unhealthy food through the establishment of a healthy canteen in the school. Foods that are considered as "healthy" and "unhealthy" are listed out in the circular and there are instructions to promote the availability of healthy options and ban or limit the availability of unhealthy options in the school canteen.[76] It is suggested that every school should have a healthy school canteen that is supervised by the school health promotion club. It is expected that the Public Health Inspector of the area should visit the school canteen once per term to observe whether the school canteen adhere to the guidelines.

Ministry of Education launched circular covers the "School Nutrition Programmes" which are implemented directly by the Ministry of Education namely (1) The Government programme to provide meals to the school children (2) " Food for Education Programme" jointly implemented by the Ministry of National Policies and Economic Affairs and World Food Programme (3) Programme to provide a glass of fresh milk/ milk packets as an additional nutritional supplement[80]. The objectives are to minimize the nutritional problems among school children, improve daily attendance of the students, promote better dietary habits and healthy lifestyle. The meals should be provided according to the menus approved by the Ministry of Education. However, the menus can be changed based on the recommendations of School Health Promotion Committee considering the regional requirements.

PROV2:

The guidelines for a healthy canteen in public sector work places has been implemented as a method of determining the acceptability of different food items based on nutrition and health factors.[81] The colour coding system aims to help consumers to identify healthy foods and foods to be limited. Foods are divided green, amber, and red categories depending on the contents, quantity, and the method of preparation of each food item. Food items that are on sale should be displayed under a background of colour of green, amber or red. A guide to categorize food items is given with the lists of food items under each category. A poster is also produced to be displayed at the canteens for consumer awareness.

PROV3:

The implementation of the school canteen policy includes training of school authorities and food handlers on healthy and unhealthy food options and preparation.[76] Annual programs conducted by field public health staff and school health promotion clubs are empowered to conduct health promotion in schools related healthy food options and diets. The guideline for the canteens of the workplaces provides information related to the healthier options together with example menus. However, the regular training and support is not clear.

PROV4:

No evidence of availability of related policy.

6 FOOD RETAIL

RETAIL1:

There are no zoning laws regarding placement of quick serve restaurants or other outlets selling mainly unhealthy foods yet. However, a decision had been taken by the Cabinet of Ministers to ban sale of unhealthy food within 100m from schools by the Minister of Health, Nutrition, and Indigenous Medicine, had been approved by the Cabinet of Ministers. But it is still in the legal draftsman's office and regulation is still not implemented.

RETAIL2:

No evidence of availability of related policy.

RETAIL3:

No evidence of availability of related policy.

RETAIL4:

No evidence of availability of related policy.

Infrastructure support domains

8 LEADERSHIP

LEAD1:

Historically, governments have taken leadership in improving food environments, population nutrition, diet related NCDs and their inequalities. Presidential Secretariat led Multisectoral Nutrition Committee having regular meetings to take decisions at national level. In addition, at Parliament level Food Advisory Committee established to ensure safe food environment. National level actions include - traffic light system for food labels, developing urban walking and exercise pathways, availability of multisectoral action plans for NCDs.

LEAD2:

In year 2018, Ministry of Health published National Salt Reduction Strategy 2018-2022 for Sri Lanka. Aim of this strategy is to reduce the estimated 10.5 grams per capita consumption to 5 grams per capita by year 2022. The key activities to achieve these targets focus on improving the surveillance of salt consumption patterns, improve the monitoring of salt contents of the food, promote formulation of foods and meals to contain less salt, implement standards for effective and accurate labelling and marketing of foods, to educate and communicate with public for low salt consumption, support development of healthy eating promoting environments.[82] In year 2016, Ministry of Health published, National Multisectoral Action Plan for the NCDs from 2016-2020, which developed in parallel to the National NCD Policy targeted to improve the consumption of fruits and vegetables. This document outlines the strategies and activities to improve the fruits and vegetables more than five servings (>400 grams) per day per capita[75]. In addition Food Based Dietary Guidelines also communicate to the public about recommended serving sizes of different types of foods to meet energy and nutrition requirements[83].

LEAD3:

Ministry of Health Sri Lanka published food based dietary guidelines for Sri Lankans from year 2000. The first edition revised in year 2010 and based on the updated information 3rd edition published in year 2016.[83] Its 4th edition launched in Colombo during the month of December 2020. These guidelines are composed of information to make a healthy meal with different food groups and their serving sizes. The serving sizes are also presented with weight and amounts for practical use among the general public. However, compliance to the recommended dietary guidelines has not been assessed in Sri Lanka.

LEAD4:

The multisectoral action plan on Non Communicable Diseases for Sri Lanka 2016-2020, outline key strategies agreed upon for improving the food environments.[75] These include developing a mechanism to monitor food and beverage advertisements, develop national policies on food and beverage marketing to children, develop policies to promote and increase affordability of healthy foods, establish policies to tax subsidise fruits and vegetables, home gardening promotion, nutrition profiling and developing food composition tables for Sri Lanka, activities to increase the availability of foods low with salt, sugar and fat, develop a national strategy for reduction of trans fat, introduce traffic light system for food labels to highlight -salt, sugar and fat contents and introduction of taxes for unhealthy foods and beverages. In addition, this multisectoral plan also identified social marketing campaigns to improve the public awareness of impact of unhealthy food consumption as well. The need for continuation of promotion of exclusive breast-feeding practices and implementation of the Canteen Policy for school children identified as another two strategic activities within the multisectoral NCD action plan. National salt strategy also highlighted improving food environment related to salt reduction through improved food labelling, public awareness, promotion of low salt foods, regulations on marketing of high salt foods.[82]

LEAD5:

Special efforts mentioned in Annual National Budgets to allocate Monthly Allowance for Low Income families (Samurdhi program), Nutrition package for Pregnant mothers, Promotion of home gardening among rural/ urban low-income families.[84] In addition, provision of Multiple Micronutrient supplementation for the areas with

poverty, provision of Food Supplementation for the children and pregnant mothers with low weight gain irrespective of the income levels (Triposha Program). Nationwide free education and free healthcare services at the point of delivery also considered important in improving healthy food environment.

9 GOVERNANCE

GOVER1:

In Sri Lanka Technical Decisions related to food safety are taken by the Food Advisory Committee of the Ministry of Health, which is chaired by the Director General of Health Services. The members of the committee compromised from different divisions of public health of Sri Lanka[85]. Industry partners have not been involved in the development of regulations related to food safety in Sri Lanka. However, discussions usually conducted between food industry and the Ministry of Health in the implementation of approved industry regulations which sometimes required transition period.

GOVER2:

Sri Lanka has usually updated its guidelines based on the Codex standards. In addition, data available from local and international accredited organizations are constantly reviewed by public health experts for the adjustment of food policies related to different sectors e.g. adaptation of Guidelines for formula feeding and Breast Milk and the implementation of a Traffic Light system for products with added sugar.

GOVER3:

In Sri Lanka policy development process, not descriptive enough to identified as a totally transparent process compared to some other developed countries. Participation of the stakeholders from non- health sector sometimes not satisfactory. Development of policy goals/objectives are mostly based on the consensus reaching of the experts, but operational data for development of strategies are lacking in some of the sectors.

GOVER4:

There is a budget document is available for public to access. However, actual expenditure on nutrition must be requested through various government departments such as Ministry of Health, Ministry of Local Government, Ministry of Agriculture etc. on the different services provided. Annual performance review in relation to nutrition or any other ministries of Sri Lanka are not routinely conducted. Special reports related to data are available from Medical Research Institute of Ministry of Health, Annual Reports of Ministry of Health for public access.[86,87] In addition, key nutritional data surveys are conducted through the Department of Census and Statistics such as the Demographic and Health Survey, which are conducted every 5 years in Sri Lanka.[88]

10 MONITORING AND INTELLIGENCE

MONIT1:

Packaged food monitoring systems available in Sri Lanka through the Food Act of Sri Lanka and its updated regulations.[89] The implementation of the food act and its regulations are monitored by the Medical Officers of Health, Food and Drug Inspectors, and Public Health Inspectors who are distributed throughout the country. In addition, Consumer Affairs Authority of Sri Lanka conduct investigations and litigations against traders who do not oblige to fair pricing/ maximum retail pricing of any nominated food items.[90] However, island wide regular review of implementation of the food act regulations yet to conduct in national scale.

MONIT2:

In Sri Lanka Medical Research Institute of Ministry of Health conducts population level surveys to assess the micronutrient levels in regular time intervals and their survey reports published in the website for dissemination.[86] In year 2017 survey among school going adolescents for micronutrients and nutritional status monitor the

prevalence of anaemia, overweight, obesity, thinness, and other relevant micronutrient deficiencies for Sri Lanka for Sri Lanka. In addition, every 5 years Department of Census and Statistics of Government, also conducts national level Demographic and Health Survey which also monitor wasting, stunting and underweight among under 5 children, antenatal mothers as well[88]. In addition, routine collection of field health clinic data provides prevalence and incidence of anaemia, wasting, stunning, underweight children and pregnant mothers in the community. These data are annually published at National level.[87]

MONIT3:

In Sri Lanka growth and development of children is monitored through Child Health Development Records. The anthropometric measurements are taken at the field weighing clinics at village level and for the children below 5 years of age. For the children above 5 years, their growth monitored during the school health inspections of age 6, 9 and 12 years.[87] During the adulthood age 35 years are expected now to participate in healthy lifestyle clinics established at health facilities and monitor their Body Mass Index at least once a year. The information gathered from these processes together with special surveys conducted by Medical Research Institute and National NCD-STEPS survey provide the estimates of obesity for Sri Lanka.[91]

MONIT4:

National surveys – Demographic Health Survey for nutrition status among children and adults, STEPS survey to assess the prevalence of diet related NCD risk factors conducted in every five years' time. In addition, hospital Indoor Morbidity and Mortality data also provides hospital admissions/ hospital deaths/ OPD treatments and clinic follow ups of the NCD patients.

MONIT5:

No evidence of availability of related policy.

MONIT6:

All the major surveys of Nutrition, Census and Household Income and Expenditure Surveys disaggregate its data by Urban, Rural and Estate. All these surveys periodically conducted in Sri Lanka.

11 FUNDING AND RESOURCES

FUND1:

The interviewed expert's opinion is that budget for Nutrition related activities grossly inadequate as approximately <1% allocated from total health budget.

FUND2:

Research funds grossly inadequate as most of the surveys run with the support of the developmental partners.

FUND3:

There is a dedicated agency for health promotion- "Health Promotion Bureau". Specific budget line is also available, and funds are available for nutrition promotion within the population through the Health Promotion Bureau; however, this is limited.

12 PLATFORMS FOR INTERACTION

PLATF1:

Food Advisory Committee of the Ministry of Health and its sub committees represented by various experts representing non health fields too. National Nutrition Coordination Committee also compromised stakeholders from different sectors.

PLATF2:

The Directorate of Environmental and Occupational Health and the Food Advisory Committee is the platform for food safety regulations implementations together with the commercial food sector but there is no formal platform for the implementation of healthy food promotion policies together with commercial partners.

PLATF3:

No evidence of availability of related policy.

PLATF4:

Even though there was a National Nutrition Secretariat functioned under the Presidential office, currently no active coordination mechanism available.

13 HEALTH IN ALL POLICIES

HIAP1:

There is no formal process to capture the nutrition, health outcomes are considered during the development of government policies related to food. However, at the cabinet of minister’s level there could be an option of providing the observations for the any proposed policies. Additionally, time to time national level steering committee appointed by President/Prime-minister in development of health/food policies with participation of multisectoral-experts.

HIAP2:

There are no uniform mechanisms. Some of the trade policies are sent to the Ministry of Health for observations but no formal assessments are currently considered.

Supplementary material 10 – Recommended actions by stakeholders

Table S3: Full list of recommended actions by stakeholders South Asia

Country	Indicator	Action	Importance	Achievability
**Policies**				
Bangladesh	COMP1	Monitoring of the healthiness of processed foods.	Very high	Low-medium
Bangladesh	COMP1	To take program to sensitize food producers through training and support to produce healthy foods.	High	Medium-high
Bangladesh	COMP2	For Food composition of the raw and processed we have to depend on Food Composition Table of Bangladesh whereas, food composition of products available at market does not comply the composition facts labelled on packages.	Very high	Medium
Bangladesh	LABEL3	Implement traffic light labelling system in Bangladesh. Nutrient density score can be used for scoring of the healthy foods.	Very high	Medium
Bangladesh	LABEL3	To introduce front-of-pack labelling regulations to monitor and strengthen consumer’s food choices.	Very high	Very high
Bangladesh	LABEL4	Make it mandatory to inform the consumer about the nutritive value of each dish served within a restaurant.	Medium-high	Medium-high
Bangladesh	PRICES1	Tax policy to make healthy foods affordable (such as importation of fruits, nuts) and tax exemption for whole cereal products etc.	Very high	Low-medium
Bangladesh	PRICES1	Making unhealthy foods (such as sugar sweetened foods) more expensive through taxing them.	Very high	Low-medium
Bangladesh	PRICES2	To impose tax/restrictions on production/import/manufacturing of unhealthy foods and beverages.	Very high	Very high
Bangladesh	PRICES2	Impose high tax on unhealthy food.	High	Low-medium
Bangladesh	PRICES3	Fiscal incentives.	Very high	Low-medium
Bangladesh	PRICES3	Enhance the availability of nutrient dense foods by give subsidies.	High	Medium
Bangladesh	PROMO1	Development of marketing strategies to restrict marketing of unhealthy diet towards children (specially media coverage).	Very high	Low-medium
Bangladesh	PROMO1/PROMO2	Ban promotion of unhealthy foods in the print, mass and social media.	Very high	Medium
Bangladesh	PROMO1/PROMO2/PROMO3	Controlling the marketing/promotion of unhealthy ready to eat processed food which is now even available at very rural areas.	Very high	Medium-high
Bangladesh	PROMO1/PROMO2/PROMO3	Regulate unhealthy marketing and promote behaviours change communication.	Very high	Medium-high
Bangladesh	PROMO2	To ban lucrative food packaging, especially packaging and marketing which target children and adolescents.	Very high	Very high
Bangladesh	PROMO3	To implement health promotion in schools and adolescent clubs, creating more demand for healthy food.	Very high	Very high
Bangladesh	PROV2	public food procurement policies.	Very high	Low-medium
Bangladesh	PROV4	Actions towards motivation of private sector producers.	Very high	Low-medium
Bangladesh	RETAIL1	School surrounding should be controlled so that unhealthy foods cannot be available.	High	High

Country	Indicator	Action	Importance	Achievability
Bangladesh	RETAIL1	Control food environment in the school and nearby area.	Very high	Low-medium
Bangladesh	RETAIL4/PROMO1/PROMO2/PROMO3	Restrictions on marketing of foods and beverages high in saturated fats, sugars and/or salt, with consumer education.	Very high	Low-medium
**Infrastructure and support**				
Bangladesh	FUND1	Allocation of adequate resources (human and financing) to improve Food-EPI programs, implement programs related to NCD, and monitoring.	Very high	Low-medium
Bangladesh	FUND1	Increase funding for food policy and environment research	Very high	Medium-high
Bangladesh	FUND2	Integrate implementation research to generate evidence about the barriers preventing the implementation of policies	Very high	Low-medium
Bangladesh	FUND3	Establish the budget tracking system to monitor public and private sector allocation and expenditure related to NCD programs	Very high	Low-medium
Bangladesh	GOVER1	Responsible behaviour from private sector and govt. policies incentivising small healthy food producers and protect them against the big commercial players in the sectors.	High	Medium
Bangladesh	HIAP2	Effective coordination of policies.	Very high	Low-medium
Bangladesh	HIAP2	Include nutrition related indicators in all social protection programs	Medium	Medium
Bangladesh	LEAD1	Advocacy and create awareness among the policy makers regarding the various food policies related to NCD.	High	High
Bangladesh	LEAD3	Food-based dietary guidelines,	Very high	Low-medium
Bangladesh	LEAD4	Coordination among entities and ministries to combat NCD by having a common roadmap of implementation.<br>indicators to be included in their performance/ target plans.	Very high	Medium-high
Bangladesh	MONIT1	Address HR gaps specially in terms of increasing monitoring and supportive supervision. Policies should be followed by strong by-laws. Authorities to have enough power delegated to make implement the by-laws.	High	Medium
Bangladesh	MONIT1	Establish effective monitoring and regulation systems.	Very high	Very high
Bangladesh	MONIT1	Strengthen the monitoring to ensure the implementation of the existing policies	Very high	Low-medium
Bangladesh	MONIT2	Enough policies but poor implementation and enforcement of those.	Very high	Low-medium
Bangladesh	MONIT4	Monitoring burden to NCDs and intake of foods on a regular basis for population (including adults and elderly people)	Very high	Low-medium
Bangladesh	MONIT5	More investment in monitoring NCDs prevalence and of food policy implementation to benchmark and identify effective approaches to improve food environments in Bangladesh.	Very high	Very high
Bangladesh	PLATF1	Need to strengthen the interorganizational collaboration implementing the existing policies.	Very high	Very high
Bangladesh	PLATF1	Need coordination of all activities related to food environment	Very high	Medium
Bangladesh	PLATF2	Public private partnership for policy implementation	Very high	Medium

Country	Indicator	Action	Importance	Achievability
Bangladesh	PLATF2	Enforcement of existing policies, monitoring of implementation, and improved coordination among various stakeholders including the private sector.	Very high	Very high
**Policies**				
India	COMP1	Food composition: Add nutrients of concern positively like Proteins, Bioavailable minerals.	High	High
India	COMP2	Regulation of unhealthy ingredients used in unorganized sector has to be implemented.	High	High
India	COMP2	Implementation of the existing regulations must be improved across the organized and unorganized sectors	High	High
India	LABEL2	Food labelling regulations are in compliance with global standards but awareness about using the labelling information for food choices needs to be promoted through public education campaigns	High	High
India	LABEL3	Define Healthy Food-—We have defined unhealthy food, but it is not complete. Foods may have many more unhealthy things apart from fat, sugar, and salt	High	High
India	LABEL3	Front-of-pack labelling needs to be implemented.	Very high	Very high
India	LABEL4	Labelling of restaurant and street foods should also be included	Medium-high	Medium
India	MONIT1	Implementation of strict monitoring policies have to be implemented with respective to unhealthy foods being sold in academic institutions	High	High
India	MONIT1	Regular studies to assess the impact of these regulations	Medium-high	Medium
India	PRICES1	Tax levied on healthy food served at outlets needs to be waived off or reduced. This will motivate service providers to provide healthy options.	Medium-high	Medium-high
India	PRICES1	Prices of fruits and vegetables are extremely elastic and responsive to price inflation. How to create local markets and storage and transport facilities is to be stressed on	Medium-high	Medium-high
India	PRICES2	Institutional catering services have constraint in order to achieve profit, they may use unhealthy ingredients like vanaspathi, oils rich in saturated fat. It is therefore essential to implement policies on reduction of price and tax on healthy foods and increase price for unhealthy options so that the use will taper down.	Medium-high	Medium-high
India	PRICES3	Promote Millet based diets	Medium-high	Medium-high
India	PRICES3	Promote kitchen based on healthy and sustainable foods	Medium-high	Medium-high
India	PRICES3	promote kitchen-based prebiotics	Medium-high	Medium-high
India	PRICES3	Product linked incentives for millets	High	Medium
India	PROMO1	Food promotion - Measures to curb negative marketing campaign for healthy foods to be included. (For example, to promote plant-based extracts blended with sugar and other ingredients to make white fluid are marketed using the brand name of milk and calling milk as unhealthy!)	High	Medium-high
India	PROMO1	Health food promotion: Promote probiotics, functional foods, natural foods, organic foods, millets, honey, butter milk, dates, etc which are excellent sources of nutrients	Medium	Medium

Country	Indicator	Action	Importance	Achievability
India	PROMO1	Healthy Food promotion: Make one session on preventive health care, that deal with importance of health foods in diet in curriculum of all medical colleges offering MBBS program	Medium-high	Medium-high
India	PROMO1	Healthy Food Promotion: Academic institutions may be involved for monitoring of health policy implementation.	Medium-high	Medium-high
India	PROMO1/PROMO2	Marketing and ads of unhealthy foods for children must be discouraged across the media	Medium-high	Medium-high
India	PROMO1/PROMO2	Banning energy dense food Adds by celebrities	High	Low-medium
India	PROV1	Food provision: Promote traditional foods rich in bioavailable nutrients	High	High
India	PROV1	The food supplementation and all the food provision programmes should also have a provision to improve diet diversity, esp. with respect to the protein and F& V component	Medium-high	Very high
India	PROV1	Tweaking the Public Distribution System to improve the region-specific pulses and veg. oils and millets	Medium-high	Medium
India	PROV3	To implement school-based awareness programmes in which nutrition is taught as a skill set<br>*Ability to differentiate between healthy and unhealthy foods<br>*Ability to read and interpret labels for food choice<br>*Importance of diet diversity	Very high	Very high
India	RETAIL1	Sale of unhealthy foods around schools needs to be regulated.	Very high	High
India	RETAIL3	Small shops selling provisions also to comply with regulations -however achieving this is a challenge.	Medium	Low-medium
India	TRADE1	Food Trade & Investment: Never open market for basic foods like milk, oil, grains, etc that can adversely affect the livelihood and economy of producers.	High	High
India	TRADE1	Agricultural support systems to create local markets for what is produced locally to improve diversity.	Medium-high	Low
**Infrastructure and support**				
India	FUND2	Regular studies on food nudges will help understand what is prompting food choices.	High	Medium
India	HIAP1	Health in all policies: Promote one health program. Human health is dependent on basic sources of food like agriculture and livestock.	High	High
India	HIAP1	Agricultural policies to be made nutrition sensitive and inclusive of climate change factors.	Medium	Medium
India	LEAD2	Awareness creation needs to be intensified among all stakeholders to differentiate between healthy and unhealthy foods.	Very high	High
India	LEAD3	Inclusion of Nutritionist and Dieticians in policy decisions.	Medium-high	Medium-high
India	MONIT1	Taxation on unhealthy foods exists but its impact not assessed.	High	Medium
India	MONIT1	Monitoring systems for unregulated food environments.	High	High
India	MONIT4	DABS (Diet and Biomarker Survey) will give input for policy decisions.	High	High
India	MONIT4	Institute based Inherent Surveillance systems to identify risk factors for NCDs.	High	High

Country	Indicator	Action	Importance	Achievability
India	PLATF4	Multi-sectoral engagement is required across I&B ministry, WCD, MoHFW, Consumer affairs, Voluntary Orgs etc.	Medium-high	Medium-high
**Policies**				
Pakistan	COMP1	Reducing fat sugar and salt in manufactured products.	High	Medium-High
Pakistan	COMP1	Food manufacturing companies should be restricted to manufacture products below prescribed standards.	Very high	High
Pakistan	COMP2	Control of Catering Companies.	Medium	High
Pakistan	LABEL1	Adoption of the Codex Alimentarius for setting up food safety and security standards, with a stringent mechanism to track progress.	Very high	Very high
Pakistan	LABEL2	There should be proper team for labelling and checking the labels of food especially locally prepared food.	High	Low
Pakistan	LABEL2	Policy for increasing the rates of harmful and such food items which are against human health so that it cannot be in reach for everyone easily.	High	Medium-High
Pakistan	LABEL3	Adopt traffic light labelling system for packaged food so even illiterate population can be wave if a food is marked red or yellow to combat obesity.	Medium-high	Medium
Pakistan	LABEL4	Display of nutritional content at all food areas (restaurants, messes, hostels, canteens etc)	Very high	Very high
Pakistan	PRICES1	Improve accessibility to fruits and vegetables to all the people by lowering the prices and setting standards for prices of these healthy foods to community.	Medium-high	High
Pakistan	PRICES1	Affordable and accessible healthy food choices for everyone in Pakistan at every level. Cost of healthy foods, including natural foods such as nuts, fruits, vegetables, and herbs, should be low as possible for every individual of society.	Very high	High
Pakistan	PRICES1	Healthy lifestyle modifications should be encouraged and facilitated by providing subsidy to James and healthy food providing hubs.	Very high	Very low
Pakistan	PRICES2	Taxes on unhealthy foods and sugary beverages.	High	Medium
Pakistan	PRICES3	Give subsides to platforms that provide healthier food choices in comparison to junk food.	High	Medium
Pakistan	PRICES3	Eliminate subsidies on all sugar and oils.	Very high	Medium
Pakistan	PROMO1	Bring in entrepreneurs in marketing and promoting healthy food in attractive packages.	Very high	Medium-High
Pakistan	PROMO1	Media campaign for some interesting program like cartoon for kids should have awareness for healthy food and avoidance of unhealthy food should be given.	Very high	Very low
Pakistan	PROMO2	Online and cable campaigns should be conducted to regulate the promotion of the unhealthy foods.	Very high	Very high
Pakistan	PROMO3	Reduce commercial pressures (e.g. marketing of unhealthy foods and beverages) particularly strategies which target children and other populations to consume food products high in fat, salt, and sugar.	High	Very high
Pakistan	PROMO3	Food restrictions and promotion of healthy food on mass level and the restaurants with healthy recipes promotions should be highlighted and rewarded.	High	Medium-high

Country	Indicator	Action	Importance	Achievability
Pakistan	PROV1	Improve school health services. Encourage children to eat healthy food and make availability of healthy food at school cafeterias.	Medium-high	Medium-high
Pakistan	PROV1	Availability of healthy food at schools, colleges universities offices and home facility.	Very high	Medium-High
Pakistan	PROV1	For boarders specifically a menu low in carbohydrates and fats should be added as an additional to normal food and annual weight measurements records should be maintained at school colleges and universities level.	Very high	Low
Pakistan	PROV1	A school food healthy lunch programme should be initiated.	High	High
Pakistan	PROV1	Healthy lunch programs must be available especially in government schools, where populations are most vulnerable.	Low-medium	Low
Pakistan	PROV1/PROV2/PROV4	To provide midday healthy meals free of cost in government schools.	Very high	High
Pakistan	PROV3	To provide nutrition education training at educational settings (e.g. school, college, university) and at community level.	Very high	High
Pakistan	PROV3	People with obesity should be sent for rehabilitation as losing weight or setting a healthy lifestyle is a difficult thing and many require the consistency that is required for adapting to this lifestyle modification. Government should employ experts to have these individuals.	Very high	Very high
Pakistan	PROV3	Government should ensure proper teaching and training of vendors prior to providing them License to sell food in a particular area.	Very high	Very low
Pakistan	PROV3	Government should have measures for provision of healthy food at mass level and implement by special team for it.	High	Very low
Pakistan	PROV3	At school level there should be parent teacher meeting in the presence of nutrition supervisor for good guidance to parents and their children regarding healthy food.	Very high	Very low
Pakistan	PROV3	Development of nutrition specific train workforce and their utilisation at approachable service outlets and in the community.	High	High
Pakistan	PROV3	There should be hiring of a nutritionist or dietitians at every hospital word and in schools and workplaces also.	High	Medium-High
Pakistan	PROV3	Cooking Training at Domestic Level and Commercial Level for Cooking Healthy Foods.	Medium-high;	Medium
Pakistan	RETAIL1	Schools should be declared no junk zone and healthy food should be provided by the school administration to every indoor student a different can be made in diet provided on the basis of their weight and BMI.	Very high	Very low
Pakistan	RETAIL1	Food licensing and annual renewal should be mandatory for all level of food businesses. The procedure should be convenient, cost effective and online.	Very high	Medium-high
Pakistan	RETAIL2	Some drinks that are high in fats or carbohydrates should be replaced by attractive healthy options, so that banning and healthy stuff is not considered as a punishment (rather than being a favour).	Very high	Very low
Pakistan	RETAIL3	Since lots of children are not attending school so it is important to provide healthy food at supermarkets at low cost. Implementation of already existing policies on food safety and provision of healthy food items at supermarket level as well as schools.	Medium-high	High
Pakistan	RETAIL3	Make the healthy food more attractive.	Very high	Medium-High
Pakistan	RETAIL3	Recognising the local culture of availability of unhealthy food is unpackaged. Can be locally cooked or available on streets.	High	High

Country	Indicator	Action	Importance	Achievability
Pakistan	RETAIL3	Healthy foods should be marketed in an attractive way and the junk food should be improved in quality with the help of scientific experts by replacing sugars with xylulose, etc.	Very high	Medium
Pakistan	RETAIL3	Unhealthy foods rich in sugars, fat, and salt should be slowly removed from the market.	Very high	Very high
Pakistan	RETAIL3	Policy for decreasing the rates of nutritious food items as compared to harmful and health damaging food items so that people find it easy and economical to go for nutritious and healthy food choices.	High	Medium-High
Pakistan	RETAIL4	Discourage the food chains selling oily and fried unhealthy food at cheap prices using substandard oils and food ingredients.	Medium-high	High
Pakistan	RETAIL4	Encourage opening of food chains using more organic ingredients.	Medium	Medium-High
**Infrastructure and support**				
Pakistan	FUND1	Proper funding should be given for implementation of healthy food and government should encourage such individuals who are meeting those criteria for healthy food at low price and even at low taxes.	Very high	Very low
Pakistan	GOVER1	Governance and accountability mechanisms are required to sustain the interventions and ensure Pakistan is on track for the global commitments. Mapping of the stakeholders to ensure alignment of the National and Provincial priorities in lieu of 18th amendments.	Very high	Very high
Pakistan	HIAP	Nutrition sensitive interventions must be made compulsory whilst designing any policy or program in sectors other than health.	Very high	High
Pakistan	LEAD3	Adoption of the WHO Eastern Mediterranean Regional Framework for actions to minimize the burden of NCDs through health interventions and the promotion of healthy diets.	Very high	Very high
Pakistan	LEAD3	The Pakistan National Dietary Guidelines need to be analysed to establish the RDA criteria for Pakistan, required for administering nutritional labels and food processing firms and regulatory entities.	Very high	Very high
Pakistan	LEAD4	To create awareness in the community regarding obesity.	Very high	Very high
Pakistan	LEAD4	To implement robust preventive campaigns and programmes for public awareness about malnutrition and alternatives for healthy food.	Very high	High
Pakistan	LEAD4	Awareness of nutrition and healthy life choices is important to combat NCDs.	Very high	High
Pakistan	LEAD4	Nutrition specific legislation and strategic framework is required to engage multistakeholder response on the double burden of disease in Pakistan to reduce NCDs.	Very high	Very high
Pakistan	MONIT1	Implementation of heavy fine policy for any food chain selling substandard food. Government should sincerely deal with this issue not by just making rules but also making possible implementation and strict action who did not follow and permanently cancer licence of such restaurants or shops etc..	Medium-high	High
Pakistan	MONIT1	There should be Awareness of cook and serving person regarding calories and nutrition scales. Also, government should send health inspector for verification of the concerned person about awareness of food hygiene and nutrition and calories status.	High	Very low
Pakistan	MONIT1	There should be monitoring team who can monitor whether the industries of various food items are following the guidelines for healthy food.	Very high	Low

Country	Indicator	Action	Importance	Achievability
Pakistan	MONIT1	To monitor the continuity of the initiatives and policies for the prevention of NCDs.	High	Very high
Pakistan	MONIT1	To implement a robust monitoring and evaluation framework.	High	High
Pakistan	MONIT1	There are many policies for nutrition, but lake of enforcement so proper enforcement is needed. Centralised nutrition policy and restrict monitoring controls within anticipated timeline is dire need of the era. Food authorities' health department and other applied agencies are required to be engaged.	Very high	Medium-High
Pakistan	MONIT2	To enable the use of telemedicine and obesity prevention services to create awareness on the prevention of obesity and to have a transdisciplinary approach in the management and follow-up of patients regarding nutritional advice.	Very high	Very high
Pakistan	MONIT5	Gap between policy and implementation must be reduced by developing workable framework of action with clear rules and responsibilities.	High	High
Pakistan	MONIT4	Recognising vulnerable population and working on prevention or treatment for example pregnancy, comorbid conditions.	Very high	Very high
Pakistan	MONIT4	Other health issues which may lead to obesity should be considered and treated accordingly obesity should not be considered as a disability rather those people should be encouraged to adapt a healthy lifestyle.	Very high	Medium
Pakistan	PLATF1	Multi-sectoral approach.	High	High
Pakistan	PLATF1	There should be centralized policy for Nutrition awareness, because there are many organizations who are doing nutrition awareness like: Punjab food authority, UNICEF, SUN, Multi sectoral Nutrition Center, and many other Organizations.<br><br>To address the lack of coordination between different institutes which results in overlapping of Nutrition awareness programs.	High	Medium
**Policies**				
Sri Lanka	COMP1	To identify food groups and regulations to establish food composition targets for sugar, salt, and saturated fat.	Very high	High
Sri Lanka	LABEL1	To strengthen the implementation of existing regulation regarding ingredient lists and nutrient declarations for them to be in line with the Codex recommendations on the labels of all packaged foods.	Very high	High
Sri Lanka	LABEL2	To strengthen the implementation of existing regulation regarding the approval and revision of claims on foods so that consumers are protected against unsubstantiated and misleading nutrition and health claims.	Very high	High
Sri Lanka	LABEL3	To strengthen and expand the implementation of existing regulation in an innovative way for example using feasible logos.	Very high	Medium
Sri Lanka	LABEL4	To develop a menu board labelling system that is consistent, simple, and clearly visible in all quick-service restaurants which is applied by the government and allows consumers to interpret the nutrient quality and energy content of foods on sale.	Medium	Low
Sri Lanka	PRICES1	To reduce the tax on fruits and vegetables.	Very high	High
Sri Lanka	PRICES1	To consider inflation and effective tax rates on foods high in fat, sugar and salt and sugar-sweetened beverages.	Very high	High
Sri Lanka	PRICES2	Tax on unhealthy foods. Include other food groups as well (i.e. trans-fat containing foods).	Very high	Medium
Sri Lanka	PRICES3	To improve food storage and transport.	Very high	Medium

Country	Indicator	Action	Importance	Achievability
Sri Lanka	PRICES4	To streamline existing programmes.	Very high	High
Sri Lanka	PROMO1	To strengthen the implementation of existing regulation and speed up the legal mechanisms.	Very high	High
Sri Lanka	PROMO2	To formulate the regulation and mechanism for implementation.	Very high	Medium
Sri Lanka	PROMO3	To strengthen and expand the implementation of existing regulation, guidelines, policies and circular.	Very high	Medium
Sri Lanka	PROV1	Clear, consistent policies are there improve clarity and check feasibility of implementation	Very high	Medium
Sri Lanka	PROV2	Clear, consistent policies are there improve clarity and check feasibility of implementation	Very high	Medium
Sri Lanka	PROV3	To introduce regular training and awareness programmes.	Very high	Medium
Sri Lanka	PROV4	To introduce regular training and awareness programmes.	Very high	Medium
Sri Lanka	RETAIL1	Clear zoning laws (i.e. 100m around schools) for unhealthy foods.	Very high	Medium
Sri Lanka	RETAIL2	Incentive polices for outlets selling fresh fruit and vegetables.	Very high	Medium
Sri Lanka	RETAIL3	To implement support systems to encourage supermarkets to promote the in-store availability of healthy foods.	Very high	High
Sri Lanka	RETAIL4	A separate government body is needed to develop guidelines and protocols to implement it.	Low	Low
**Infrastructure and support**				
Sri Lanka	LEAD1	A re-establishment of national level nutrition leadership required.	Very high	High
Sri Lanka	LEAD2	Establish intake targets for all the nutrients of concern.	High	High
Sri Lanka	LEAD3	To strengthen the implementation of food-based dietary guidelines.	Very high	High
Sri Lanka	LEAD4	To revisit existing NCD prevention plans and identify the gaps.	Very high	High
Sri Lanka	LEAD5	Need to have sustainable (Evidence/ scientifically Informed) programs, policies for vulnerable populations and implementation plans.	Very high	Medium
Sri Lanka	LEAD5	Programmes and policies must be based on Evidence based scientific data and implementation of ad hoc programmes should not be allowed.	Very high	Medium
Sri Lanka	GOVER1	Strengthen existing procedures and make them more robust to restrict commercial influence. This may require legislation and implementation.	Very high	Medium
Sri Lanka	GOVER2	Establish a clear framework for using evidence in systematic manner for the development of food policies.	Very high	High
Sri Lanka	GOVER3	All relevant stakeholders should follow the accepted procedures and relevant information should be available in public domain.	High	Medium
Sri Lanka	GOVER3	Policies and procedures are implemented for ensuring transparency in the development of food policies.	High	Medium
Sri Lanka	GOVER4	Need to establish mechanisms to publish the documents/relevant information in a timely manner.	High	Medium

Country	Indicator	Action	Importance	Achievability
Sri Lanka	MONIT1	Gaps to be identified related to the M/E and develop mechanisms to address those.	Very high	Medium
Sri Lanka	MONIT2	Adult nutrition and intakes Monitoring mechanisms to be established/strengthened – esp. regarding adult nutrition status and nutrient intake.	High	High
Sri Lanka	MONIT3	A mechanism for adult OW /OB monitoring to be established through national surveys such as STEPS, as well as surveys conducted by universities and other organisations.	High	High
Sri Lanka	MONIT4	Monitoring mechanisms to be strengthened/established.	Very high	High
Sri Lanka	MONIT4	Continue national surveys (such as STEPS), establish efficient disease registries (ideally computerised), improve speed of death registration data releases, and analyse deaths data.	Very high	High
Sri Lanka	MONIT5	Mechanisms to be strengthened/ established for evaluations.	High	High
Sri Lanka	MONIT6	Establish a robust monitoring mechanism.	High	High
Sri Lanka	FUND1	The government to identify a source of dedicated funds that are ear marked for nutrition related interventions. Suggested is a proportion of taxes from SSBs, tobacco or alcohol.	Very high	Medium
Sri Lanka	FUND2	Government-funded research needs to target for improving food environments, reducing obesity, NCDs, and their related inequalities.	Very high	Medium
Sri Lanka	FUND2	University programs need to train researchers.	Very high	Medium
Sri Lanka	FUND3	Health Promotion Bureau needs to be strengthened.	Very high	Medium
Sri Lanka	PLATF1	To strengthen existing committees and councils.	Very high	High
Sri Lanka	PLATF1	To strengthen the Nutrition Steering Committee, chaired by the Secretary of Health, and the Technical Advisory Committees, chaired by the Director General.	Very high	High
Sri Lanka	PLATF1	To appoint a permanent authority focused on the prevention of NCDs which coordinates different stakeholders.	Very high	High
Sri Lanka	PLATF1	For the government to establish a Taskforce/Coordination to be Chaired by a President to facilitate coordination across different ministries.	Very high	High
Sri Lanka	PLATF2	To have a coordinating body which enables formal platforms between the government and the commercial food sector to implement healthy food policies.	Very high	High
Sri Lanka	PLATF3	To have a coordinating body which enables formal platforms for the regular interaction between government and civil society on food policies and other strategies to improve population nutrition.	Very high	High
Sri Lanka	PLATF4	To have a coordinating body which enables the government to lead a broad, coherent, effective, integrated, and sustainable systems-based approach with local organizations to improve the healthiness of food environments and a national level.	Very high	High
Sri Lanka	HIAP1	Need to implement a task force under the president to coordinate and guide the research, policy making, and advocacy activities for promoting healthy food environment.	Very high	Medium

Country	Indicator	Action	Importance	Achievability
Sri Lanka	HIAP2	Need to implement health impact assessments to assess and consider health impacts during the development of other non-food policies.	Very high	Medium

References

1. Raboy M, Padovani C. Mapping Global Media Policy: Concepts, Frameworks, Methods. *Communication, culture & critique* 2010; **3**(2): 150-69.
2. WHO. Global database on the Implementation of Nutrition Action (GINA) Geneva: World Health Organization 2012.
3. Vandevijvere S, Swinburn B. Pilot test of the Healthy Food Environment Policy Index (Food-EPI) to increase government actions for creating healthy food environments. *BMJ Open* 2015; **5**(1): e006194.
4. Phulkerd S, Lawrence M, Vandevijvere S, Sacks G, Worsley A, Tangcharoensathien V. A review of methods and tools to assess the implementation of government policies to create healthy food environments for preventing obesity and diet-related non-communicable diseases. *Implementation Science* 2016; **11**(1): 15.
5. Swinburn B, Vandevijvere, S. INFORMAS Protocol: Public Sector Module - Healthy Food Environment Policy Index (Food-EPI). . Online: The University of Auckland, 2017.
6. WHO. Global action plan for the prevention and control of noncommunicable diseases 2013-2020. 2013. https://www.who.int/publications/i/item/9789241506236 (accessed February 2024).
7. INFORMAS. Publications by Module. 2013. https://www.informas.org/publications/. (accessed February 2024).
8. Ng S, Swinburn B, Kelly B, et al. Extent of implementation of food environment policies by the Malaysian Government: gaps and priority recommendations. *Public Health Nutrition* 2018; **21**(18): 3395-406.
9. Vandevijvere S, Mackay S, Swinburn B. Measuring and stimulating progress on implementing widely recommended food environment policies: the New Zealand case study. *Health Research Policy and Systems* 2018; **16**(1): 3.
10. Phulkerd S, Vandevijvere S, Lawrence M, Tangcharoensathien V, Sacks G. Level of implementation of best practice policies for creating healthy food environments: assessment by state and non-state actors in Thailand. *Public Health Nutrition* 2017; **20**(3): 381-90.
11. Kwon J, Reeve E, Mann D, Swinburn B, Sacks G. Benchmarking for accountability on obesity prevention: evaluation of the Healthy Food Environment Policy Index (Food-EPI) in Australia (2016-2020). *Public Health Nutr* 2022; **25**(2): 488-97.
12. Vanderlee L, Goorang S, Karbasy K SA, L'Abbe M. C. Creating healthier food environments in Canada: Current policies and priority actions - Summary report. . Toronto: University of Toronto, 2017.
13. International Network for Food and Obesity / Non-communicable Diseases (NCDs) Research Monitoring and Action Support. INFORMAS Protocol: Public Sector Module - Healthy Food Environment Policy Index (Food-EPI). New Zealand: The University of Auckland, 2017.
14. Higgins JPT, Thomas J, Cumpston M, Li T, Page MJ, Welch VA. Cochrane Handbook for Systematic Reviews of Interventions version 6.4 (updated August 2023); 2023.
15. Nyanchoka L, Tudur-Smith C, Porcher R, Hren D. Key stakeholders' perspectives and experiences with defining, identifying and displaying gaps in health research: a qualitative study protocol. *BMJ Open* 2019; **9**(8): e027926.
16. Hans F, Day T, Röser F. Actor and Policy Mapping Tool. 2020. https://newclimate.org/2020/02/25/actor-and-policy-mapping-tool/.
17. World Health Organization. Limiting Trans Fatty Acids in Foodstuffs Regulations, 2021- Bangaladesh- Global database on the Implementation of Nutrition Action (GINA). 2022. https://extranet.who.int/nutrition/gina/en/node/737842023).
18. Ministry of Food- Bangaladesh. The Food Safety Act Dhaka, 2013.

19. Bangladesh Standards and Testing Institution (BSTI). e-Catalogue and Bangladesh Standards Sale. 2006. https://bstibds.com/single_product/2062022).
20. Bangladesh Standards and Testing Institution (BSTI). Bangladesh Standard for The Labelling of Prepackaged Foods. Dhaka, 2008.
21. Non-communicable Disease Control Programme-Directorate General of Health Services. Multi-sectoral action plan for prevention and control of non-communicable diseases 2018-2025 - Bangladesh Dhaka, 2018.
22. World Health Organization. Food Safety (Labelling) Regulations 2017- Bangladesh 2017. https://extranet.who.int/nutrition/gina/en/node/39319.
23. Ministry of Law Justice and Parlimentary Affairs - Bangladesh. The Pure Food Ordinance, 1959 (Amended 2005) Dhaka, 2005.
24. Planning Commission Government of Bangladesh. Social Security Policy Support (SSPS) Programme. Government's open market system (OMS). 2022. https://socialprotection.gov.bd/social-protection-pr/open-market-sales-oms/.
25. Food Planning and Monitoring Unit (FPMU)-Bangladesh. National Food Policy Plan of Action. Dhaka, 2008.
26. World Bank. Bangladesh - Income Support Program for the Poorest Project : Empowering Poor Mothers to Improve Child Nutrition (English). 2019. http://documents.worldbank.org/curated/en/378671568292184361/Bangladesh-Income-Support-Program-for-the-Poorest-Project-Empowering-Poor-Mothers-to-Improve-Child-Nutrition.
27. Ministry of Health and Family Welfare (MOHFW)-Bangladesh. National Nutrition Policy, 2015.
28. Nahar Q, Choudhury, S., Faruque, M. M., Saliheen Sultana, S. S., & Siddiquee, M. A. Dietary guidelines for Bangladesh, 2014.
29. Shahan A.M, Ferdous J. Opening the policy space: the dynamics of nutrition policy making in Bangladesh. 2017.
30. Md. M. Islam Bulbul, Iftekhar Rashid. Developing the second National Plan of Action for Nutrition in Bangladesh: Emergency Nutrition Network (ENN), 2018.
31. Bangladesh National Nutrition Council Office. Nutrition Related All Policy/Plan/Act/Strategy. 2022. https://bnnc.portal.gov.bd/site/page/04d70926-34d5-4e62-91d4-810bbeb162f0/-2023).
32. Food Safety and Standards Authority of India. Report of expert group on consumption of fat, sugar and salt and its health effects on Indian population: Food Safety and Standards Authority of India, 2017.
33. Food Safety and Standards Authority of India. Draft Guidelines for Making Available Wholesome, Nutritious, Safe and Hygienic Food to School Children in India, 2015.
34. Food Safety and Standards Authority of India. Eat Right Campus. 2019. https://www.eatrightindia.gov.in/EatRightCampus/home.
35. Food Safety and Standards Authority of India. The Orange Book for Campuses. New Delhi: Food Safety and Standards Authority of India, 2020.
36. Food Safety and Standards Authority of India. Food Safety and Standards (Labelling and Display) Regulations 2020. New Delhi: Food Safety and Standards Authority of India, 2020.
37. Food Safety and Standards Authority of India. Guidance note on Display of Information in Food Service Establishment, Menu Labelling. New Delhi, 2021.
38. Food Safety and Standards Authority of India. Food Safety and Standards (Advertising and Claims) Regulations, 2018. New Delhi, 2018.

39. Food Safety and Standards Authority of India. Guidelines for Promoting Wholesome and Nutritious Food and Restricting/ Limiting the Availability of Foods High in Fat, Sugar and Salt (HFSS Foods) among School Children. 2018.
40. Central Board of Indirect Taxes and Customs- India. Goods and Services Tax, 2022.
41. Ratna B, Rasul B, S.Sanandakumar. In a first, Kerala imposes 14.5% 'fat tax' on junk food. The Economic Times 2016.
42. Education Post. India applies Sin Taxes on Sugary Carbonated Drinks. 2018.
43. Department of food and public distribution. National Food Security Act (NFSA), 2013. 2013.
44. World Food Programme India. www1.wfp.org/countries/india.
45. Ananya Tewari. CBSE lists detailed measures to limit junk food in schools. 2016. https://www.downtoearth.org.in/news/health/cbse-lists-detailed-measures-to-limit-junk-food-in-schools-52428.
46. Snaxsmart. Healthy Vending Machine. 2019. https://snaxsmart.com/about-snaxsmart-snacks-vending-machine/.
47. Food Safety and Standards Authority of India. Food Safety Training and Certification (FoSTaC) https://www.fssai.gov.in/cms/fostac.php#:~:text=FSSAI&text=Food%20Safety%20Training%20%26%20Certification%20is,thereof%20on%20all%20their%20premises.
48. Food Standards and Standards Authority of India. Serve Safe Initiative. https://snfportal.in/jsp/servesafe.jsp.
49. Food Safety and Standards Authority of India. The Orange Book- Your Guide for Safe and Nutritious Food at the Workplace. New Delhi: Food Safety and Standards Authority of India,; 2018.
50. National Institute of Nutrition. Dietary Guidelines for Indians- A Manual. 2011.
51. Department of women & child development - India. National Nutrition Policy, 1993.
52. Food Safety and Standards Authority of India. Food Authority. 2022. https://www.fssai.gov.in/cms/food-authority.php.
53. National Institute of Nutrition. Publications. 2022. https://www.nin.res.in/index.html#.
54. Ministry of Health & Family Welfare-Government of India. National Family Health Survey (NFHS-5). New Delhi, 2021.
55. Ministry of Health & Family Welfare-Government of India. National Multisectoral Action Plan for Prevention and Control of Common Noncommunicable Diseases (2017-2022). New Delhi, 2017.
56. Avani Kapur, Shukla R. Saksham Anganwadi and POSHAN 2.0: Accountability Initiative, 2022.
57. Ministry of Health & Family Welfare-Government of India. The Department of Health Research. 2022. https://dhr.gov.in/about-us/about-department.
58. Ministry of Health & Family Welfare-Government of India. Department of Health and Family Welfare. 2022. https://main.mohfw.gov.in/.
59. U.S. Department of Agriculture. Pakistan’s New Labeling Requirements. 2019. https://www.fas.usda.gov/data/pakistan-pakistan-s-new-labeling-requirements.
60. Punjab Food Authority. Introduction to Punjab Food Authority. 2012. http://www.pfa.gop.pk/overview/.
61. Mkambula P, Mbuya MNN, Rowe LA, et al. The Unfinished Agenda for Food Fortification in Low- and Middle-Income Countries: Quantifying Progress, Gaps and Potential Opportunities. *Nutrients* 2020; **12**(2).
62. Dawn News. Tax for better health. 2018.

63. Pakistan Today. Some questions about the food subsidy- How to get buy-in from retailers? Pakistan Today. 2022.
64. Ary News. Sindh govt bans energy drinks and snacks at schools. . Ary News. 2018.
65. The News. Food authority teams inspect Peshawar schools. 2018.
66. Pakistan Today. Govt urged to ensure food quality at school canteens. Pakistan Today. 2015.
67. Raza A. PFA sets guidelines on food at school, college canteens. The News. 2017.
68. Ministry of National Food Security and Research. Introduction. 2022. http://www.mnfsr.gov.pk/Detail/ZGMxZmE5Y2EtN2MwMC00MTliLWIyMDAtZGNiNTQ3NmUyMzE32023).
69. Directorate of Non Communicable Diseases. National salt reduction strategy 2018-2022, 2018.
70. Ministry of Health Nutrition and Indigenous Medicine. National Multisectoral Action Plan for Prevention and Control of Non Communicable Diseases 2016.
71. Ministry of Health. Food (Labelling and Advertising) Regulations-2005 2005.
72. MInistry of Health Nutrition and Indigenous Medicine. Food (Colour Coding for Sugar levels) Regulations 2016. 2016.
73. Ministry of Health Nutrition and Indidegenous Medicine. Food (Colour coding for sugar, salt and fat) Regulations-2019 Colombo, 2019.
74. Ministry of Health Nutrition and Indigenous Medicine. Nutrient Profile Model for Sri Lanka to regulate marketing of Food and Non Alcoholic Beverages to children. Colombo, 2018.
75. Ministry of Health Nutriton and Indigenous Medicine. National Multisectoral Action Plan for The Prevention and Control of Non-Communicable Diseases 2016-2020. Colombo, 2016.
76. Ministry of Education-Sri Lanka. School Canteen Policy 2007.
77. Department of Fiscal Policy. The Gazette of the Democratic Socialist Republic of Sri Lanka (Extraordinary) 2019.
78. Department of the Commissioner General of Samurdhi-Sri Lanka. "Poshana Malla" Nutrition Stamp. 2010. https://www.gic.gov.lk/gic/index.php?option=com_info&id=477&task=info&lang=en.
79. Ministry of Women Child Affairs and Social Empowerment. Programme to Provide a Nutrition Allowance of Rs.20,000 to Pregnant and Lactating Mothers. 2015. http://www.childwomenmin.gov.lk/institutes/childrens-secretariat/main-development-programmes.
80. Ministry Education. Manual on School Nutrition Programme 2017. http://www.moe.gov.lk/english/images/publications/2018/health/sch_manual_e.pdf.
81. Ministry of Health Nutrition and Indigenous Medicine. Guidelines for a healthy canteen in work places. Colombo, 2013.
82. Ministry of Health Nutriton and Indigenous Medicine. National Salt Reduction Strategy 2018-2022: Sri Lanka. Colombo, 2018.
83. Ministry of Health Nutriton and Indigenous Medicine. Food Based Dietary Guidelines for Sri Lankans. Colombo, 2016.
84. Ministry of Finance. Budget Speech- 2021. Colombo, 2020.
85. Food Control Administration Unit. Food Advisory Commitee. 2020. http://eohfs.health.gov.lk/food/index.php?option=com_content&view=article&id=20&Itemid=161&lang=en.
86. Medical Research Institute. Nutriton Survey Reports. 2020. https://www.mri.gov.lk/units/nutrition/survey-reports/.

87. Family Health Bureau. Annual Report of the Family Health Bureau-2018. Colombo, 2020.
88. Department of Census and Statistics. Demographic Health Survey- 2016, Sri Lanka. Colombo, 2017.
89. Food Control Administration Unit. Food Act and Current Regulations, 2020.
90. Consumer Affairs Authority. Consumer Affairs Authority Act - No 09 of 2003. Colombo: Consumer Affairs Authority- Sri Lanka, 2020.
91. Ministry of Health Nutrition & Indigenous Medicine-Sri Lanka. Non-Communicable Disease Risk Factor Survey Colombo 2015.
